# *Physalis floridana CRABS CLAW* mediates neofunctionalization of *GLOBOSA* genes in carpel development

**DOI:** 10.1093/jxb/erab309

**Published:** 2021-06-28

**Authors:** Pichang Gong, Chunjing Song, Hongyan Liu, Peigang Li, Mingshu Zhang, Jisi Zhang, Shaohua Zhang, Chaoying He

**Affiliations:** 1 State Key Laboratory of Systematic and Evolutionary Botany, Institute of Botany, Chinese Academy of Sciences, Nanxincun 20, Xiangshan, Beijing, China; 2 University of Chinese Academy of Sciences, Beijing, China; 3 The Innovative Academy of Seed Design, Chinese Academy of Sciences, Beijing, China; 4 University of Nottingham, UK

**Keywords:** B-function MADS-box gene, carpel development, CRC transcription factor, floral organ identity and functionality, fruit evolution, *Physalis floridana*

## Abstract

Floral B-function MADS-box genes, such as *GLOBOSA* (*GLO*), function in corolla and stamen organ identity specification. The functions of these genes outside these floral whorls are rarely reported. *DOLL1* is a *GLO* gene controlling corolla and androecium organ identity. In this study we found that, in *Physalis floridana double-layered-lantern 1* (*doll1*) mutant pollinated with wild-type pollen, fruit set was extremely low, indicating that *doll1* females are dysfunctional. Stigma and style structure, stigma receptivity, pollen tube guidance, and embryo sac development were also impaired in *doll1*. *P. floridana CRABS CLAW* (*PFCRC*), predominantly expressed in carpels, was repressed in *doll1* native carpels. Loss-of-function of *PFCRC* altered carpel meristem determinacy, carpel closure, and ovule number, and the resultant ‘pistil’ consisted of multiple spirally-arranged dorsiventral carpels occasionally with 1–2 naked ovules on the margin and trichomes at each mutated carpel tip, implying an alteration of carpel organ identity. Regulatory and genetic interactions between B-class MADS-box genes and *PFCRC* were revealed in a context-dependent manner in floral development. Our work reveals a new role for the B-function genes in carpel and ovule development via regulating *PFCRC*, providing a new understanding of genetic regulatory networks between MADS-domain and CRC transcription factors in mediating carpel organ specification, functionality, and origin.

## Teaser

The regulatory and genetic interactions of *DOLL1*, a typical floral B-function gene with *PFCRC,* a key carpel regulator, reveal new functional roles for floral development of *P. floridana*.

## Introduction

Floral homeotic mutants have yielded the floral ABC and quartet models of floral development, with further implications for the origin of true flowers (Schwarz-Sommer *et al.*, 1990; Coen and Meyerowitz, 1991; Weigel and Meyerowitz, 1994; Ma and dePamphilis, 2000; Theissen and Saedler, 2001). Four basic types of genes (A, B, C, and E) have been characterized, and they are generally MADS-box genes. Floral A-function genes define the sepal organ identity (Mandel *et al.*, 1992; Zhao *et al.*, 2013); B-function genes specify the petal and stamen organ identity (Bowman *et al.*, 1989; 1991; Jack *et al.*, 1992; Tröbner *et al.*, 1992; Krizek and Meyerowitz, 1996), C-function genes determine the stamen and carpel organ identity (Yanofsky *et al.*, 1990; Bowman *et al.*, 1991; Coen and Meyerowitz, 1991; Lenhard *et al.*, 2001), while E-function genes usually act as cofactors or glues of various floral function genes (Honma and Goto, 2001; Ditta *et al.*, 2004). Duplication and subsequent divergence of floral B-function MADS-box genes have been demonstrated to play roles in diversification of floral morphology (Vandenbussche *et al.*, 2004; de Martino *et al.*, 2006; Rijpkema *et al.*, 2006; Geuten and Irish, 2010; Zhang *et al.*, 2015). However, the developmental functions of these genes are basically restricted to the corolla and stamens (Jack *et al.*, 1992; Tröbner *et al.*, 1992; van der Krol *et al.*, 1993). Gene duplication and sub-functionalization patterns of the paralogous genes are prevalent and differ among species, and gene function fluidity could occur between non-orthologous genes or different gene families (Panchy *et al.*, 2016; Scutt *et al.*, 2006; Rensing, 2014). Moreover, functions of substantial genes could have been diversified, i.e. neofunctionalization during evolution, thus becoming pleiotropic. For example, Arabidopsis *CRABS CLAW* (*CRC*) function is required for nectary development and elaboration of carpel morphology activity (Alvarez and Smyth, 1999; Bowman and Smyth, 1999; Lee *et al.*, 2005a), while its orthologs in other plant species either exert essential roles in nectary development (Lee *et al.*, 2005b; Morel *et al.*, 2018), or exert a relatively conserved role in carpel development (Fourquin *et al.*, 2005; Orashakova *et al.*, 2009), and even determine carpel organ identity only in rice (Yamaguchi *et al.*, 2004; Sugiyama *et al.*, 2019). The knowledge gained from model plants thus cannot be directly extrapolated to evolutionarily distant taxa. Therefore, additional evidence from various non-model species might be informative for understanding the functional evolution of orthologous genes in the origin and evolution of morphological traits.

*Physalis* is a genus in the Solanaceae family exhibiting a novel morphology of the fruiting calyx known as inflated calyx syndrome (ICS), or informally as ‘Chinese lantern’ (He *et al.*, 2004; He and Saedler, 2005; Zhao *et al.*, 2013). This nightshade lineage includes several popular vegetables as well as fruits, including cape gooseberry, husk tomato, or tomatillo (Wang *et al.*, 2014). Two *GLOBOSA* genes, *PFGLO1* and *PFGLO2*, have been found in *Physalis* (Zhang *et al.*, 2014a). Down-regulating *PFGLO2* only affects pollen maturation, while single mutation of *PFGLO1* leads to the *double-layered-lantern1* (*doll1*) mutant in which the corolla and androecium are transformed into the calyx and gynoecium (Zhang *et al.*, 2014a; 2015), indicating that *DOLL1* (*PFGLO1*) is a typical B-function gene. However, the difficulty in obtaining hybrid berries pollinated with wild-type (WT) pollen implies that the carpel functionality in *doll1* must be seriously dysfunctional, and genomic locus of *PFGLO1* has completely rescued the *doll1* abnormalities (Zhang *et al.*, 2014a), suggesting neofunctionalization of a floral B-function gene. To address this question, we performed a detailed dissection of the morphological and functional abnormalities of *doll1* carpels, and found that stigma and style structure, embryo sac development, stigma receptivity, pollen tube elongation, and guidance induced by ovules were impaired as a result of DOLL1 directly targeting *Physalis floridana CRABS CLAW* (*PFCRC*). Interestingly, genetic manipulations revealed that *PFCRC* was involved in specifying carpel organ identity, closure and functionality. Moreover, in the absence of *DOLL1*, *PFCRC* also contributed to petal and stamen organ identity specification via repressing *PFGLO2*. Furthermore, overexpressing *PFCRC* could increase organ size in wild type *P. floridana* and also improve the female functionality in *doll1*. Taken together, our study confirms a new role of B-function MADS-box genes in carpel development, demonstrates functional pleiotropy of *PFCRC*, and elucidates the regulatory and genetic interactions of *DOLL1* and *PFCRC*. We thus provide new insights into interactions of MADS-domain and CRC transcription factors in regulating floral organ identity specification and fertilization-associated functionalities, particularly in carpel origin and fruit diversity in angiosperms.

## Materials and methods

### Plant materials

*Physalis floridana* P106 (He and Saedler, 2005), the *doll1* mutant (Zhang *et al.*, 2014a), *Nicotiana benthamiana,* and all transgenic plants generated in this work were grown in a growth chamber under 16 h light (115-128 μmol m^-2^ s^-1^) and 8 h dark cycle conditions with a temperature cycle of 24 °C/22 °C at the Institute of Botany, Chinese Academy of Sciences (IBCAS, Beijing, China).

### Genotypic analysis of F_2_ genetic populations

Total genomic DNA isolation of each individual in the *pfcrc-cas9-1*-related F_2_ populations from young leaves, and PCR analyses were performed by using a *TransDirect* Plant Tissue PCR Kit (TransGen, Beijing, China). The PCR products amplified by using gene-specific primers (Supplementary Table S1) were separated on 1% agarose gels, and the band types of each individual were recorded for the corresponding genotype.

### Scanning electron microscopy (SEM) and micro-CT assays

Floral organs were immediately fixed in formaldehyde acetic acid (FAA) solution (37–40% formaldehyde: glacial acetic acid: 70% alcohol, 5:5:90, v/v/v) for 24 h after vacuum infiltration for a short-time, and then dehydrated in a graded ethanol series. The dehydrated materials were dried up to critical-point in liquid CO_2_, mounted on metallic stubs, and shadowed with gold. Images for SEM and micro-CT were captured with a scanning electron microscope Hitachi S-800 (Hitachi, Japan) and SkyScan micro-CT system SkyScan1172 (Bruker, Belgium), respectively.

### Morphological and histological analyses

Morphology of floral organs was dissected using a Greenough stereo microscope (Leica S9E; Leica, Wetzlar, Germany), and photographed by an encoded stereo microscope (Leica M205 C; Leica, Wetzlar, Germany). Pollen maturation was investigated using iodine-potassium iodide (I_2_-KI) staining and was photographed using an upright Leica DM6 B microscope (Leica, Wetzlar, Germany). Plant architectures were imaged with a Nikon single lens reflex camera D850 (Nikon, Japan). For paraffin sectioning, the subjected materials were fixed overnight in FAA solution, after which they were dehydrated in a graded ethanol series and embedded in paraffin wax (Sigma-Aldrich, St. Louis, USA). Tissue sections were cut with a microtome and stained with toluidine blue. The slides were examined and photographed using an upright Leica DM6 B microscope (Leica, Wetzlar, Germany).

### Microscopy of the embryo sac

Ovules of WT and *doll1* native carpels at different developmental stages were separated and lysed in enzymatic hydrolysate (0.6 M D-Mannitol, 10 mM MES, 1 mM CaCl_2_, pH = 5.7; 0.1% BSA, 1.5% cellulase RS, 0.75% macerozyme, and 5 mM β-mercaptoethanol) for at least 30 min. After that, they were vacuum infiltrated for 3 h in 4% glutaraldehyde solution and were substituted with benzyl benzoate and benzyl alcohol solution (3:1, v/v) three times after gradient dehydration with ethanol. The embryo sac morphology was photographed using an Olympus FV1000MPE confocal laser scanning microscope (Olympus, Japan).

### Stigma receptivity assay

Stigma receptivity of matured pistils was performed via the benzidine-hydrogen peroxide method (1% benzidine: 3% hydrogen peroxide: distilled H_2_O, 4:11:22, v/v/v; Galen and Plowright, 1987). The maximal reaction time and the stigma browning degree were the two indices for evaluating stigma receptivity. The reaction time was calculated upon the stigma being placed into the reaction buffer until no bubbles were produced, and at that time the stigma browning degree was measured by hue value using Photoshop software.

### Pollen tube growth observations

Pistils at 24 and 48 h after artificial pollination were fixed by Carnoy’s fixative (ethyl alcohol: acetic acid, 3:1, v/v) overnight at 22–25 °C when they were decolorized, and then transferred into NaOH solution (8.0 M) overnight. Images were captured by microscopy using a Carl Zeiss Axio Imager A1 (Carl Zeiss, Gottingen, Germany) in the ultraviolet spectrum after staining with 0.01% aniline blue solution.

### Semi-*in vitro* ovule-induced pollen tube growth

After artificial pollination, the WT stigma and style above the ovary were cut using a sharp blade. These were placed near to the mature ovules of the WT and the *doll1* mutant at equal distances, and then cultivated on solid medium (18% sucrose, 0.01% boric acid, 0.1 mM CaCl_2_, 1 mM MgSO_4_ and 0.5% agarose) for 10–14 h at 22–25 °C until growth of pollen tubes out of the style was observed. Images were captured by a Leica M165 FC fluorescent stereo microscope (Leica, Wetzlar, Germany).

### Differentially expressed gene (DEG) scanning

Total RNA of floral buds from wild type (WT) and *doll1* mutants were isolated using the SV Total RNA Isolation System (Promega, Madison, USA). RNA-seq was performed by the Beijing Genome Institute (Shenzhen, China). The absolute value of log_2_ ratio ≥2 and *P* value ≤0.01 was used as the threshold to identify DEGs between WT and *doll1* mutants.

### RT-PCR analyses

The involved plant organs or tissues were harvested and immediately frozen and stored in liquid nitrogen for total RNA isolation. About 1 μg of total RNA was used for first-strand cDNA synthesis with the oligo (dT)_18_ primer using an M-MLV cDNA synthesis kit (Invitrogen, China) in a 20 μl volume. For semi-quantitative RT-PCR, a 1 μl aliquot of the synthesized cDNA stock solution was used. PCR products were detected on 1% agarose gels and photographed with an ultraviolet imager, and typical results were presented. Quantitative RT–PCR (qRT–PCR) was performed on an Agilent Mx3000P qRT–PCR system (Agilent, Waldbronn, Germany) using SYBR^®^*Premix Ex Taq*^TM^ (TAKARA, Japan) via an amplification procedure consisting of 95 °C for 30 s, followed by 40 cycles of 95 °C for 5 s, 56 °C for 20 s, and 72 °C for 20 s, followed by a dissociation curve analysis. The *PFACTIN* and *PFTUBULIN* genes were used as internal reference genes. Gene-specific primers used are listed in Supplementary Table S1.

### Tissue *in situ* hybridization

The 333 bp *PFCRC*- and 259 bp *DOLL1*-specific sense and antisense probes were synthesized using the T_7_ RNA polymerase driven by a T_7_ promoter and labelled with digoxigenin using the DIG RNA Labelling Kit (SP6/T7; Roche, Mannheim, Germany). Hybridization was performed by using around 9 ng μl^-1^ RNA probes at 50 ℃ for 16 h. Images were captured under an upright Leica DM6 B microscope (Leica, Wetzlar, Germany).

### Virus-induced gene silencing (VIGS) analysis

A 211 bp coding sequence (CDS) of *PFCRC* was introduced into the TRV-mediated VIGS vectors, as described previously (Zhang *et al.*, 2014b). Briefly, the TRV mediated VIGS vectors were infiltrated into leaves of 2-week-old seedlings of *P. floridana* mediated by *Agrobacterium tumefaciens* strain LBA4404. The gene silencing in *PFCRC*-VIGS flowers was confirmed by qRT-PCR analyses.

### Sub-cellular localization analysis

The open reading frame (ORF) of *PFCRC* was cloned into the binary vector *Super1300*-green fluorescence protein (GFP) gene using the *Xba*I and *Kpn*I restriction sites to form a *Super::PFCRC-GFP* in-framed plasmid. After sequence confirmation, the resulting construct was transformed into *A. tumefaciens* strain LBA4404 and then injected into leaf epidermal cells of *Nicotiana benthamiana* or *P. floridana*. At 48 h after injection, the fluorescence signal of GFP was detected using an Olympus FV1000MPE confocal laser scanning microscope (Olympus, Japan).

### Generation of transgenic *Physalis* plants

For *PFCRC* overexpression, a *Super::PFCRC-GFP* in-frame construct was transformed into WT and *doll1* heterozygotes (*DOLL1*^+/-^) as explants. For gene editing, a PCR fragment harbouring two sgRNA expression cassettes targeting two specific 20 bp sequences in the first and the second exon of *PFCRC* under the control of each Arabidopsis U6 gene promoter was amplified from a pCBC-DT1T2 vector using two pairs of primers in one PCR reaction (Supplementary Table S1). This fragment was cloned into the plant binary vector PHSE401 using the restriction-ligation reactions (Xing *et al.*, 2014) to generate a *PFCRC*-CRISPR/Cas9 plasmid for WT *Physalis* transformation. The genetic transformation was mediated by *A. tumefaciens* strain LBA4404, and the procedure followed is described in He and Saedler, 2005).

### Yeast one-hybrid assay

The putative *PFCRC* promoter fragment was amplified directly using a constructed pEASY^®^-Blunt Cloning vector (TransGen Biotech, Beijing, China) as the template. Triple tandem repeats of each CArG-box motif were synthesized. All DNA fragments were cloned into the Y1H vector pAbAi (Clontech, Mountain View, USA) using suitable restriction sites (Supplementary Table S1). Then, each assembled pAbAi construct was linearized with *Bstb*I and transformed into *Saccharomyces cerevisiae* Y1HGold strain according to the Yeast Transformation System 2 Manual (Clontech, Mountain View, USA). Clones carrying the desired DNA fragment were screened for auto activation on synthetic uracil dropout medium supplemented with Aureobasidin A in the concentration of 100–900 ng ml^-1^, as indicated (Clontech, Mountain View, USA). Bait strain colonies that showed no auto activation were selected and transformed with prey plasmids *MPF3*- (*MADS-box gene 3* from *P. floridana*), *DOLL1* (*PFGLO1*, *P. floridana GLOBOSA* 1)-, *PFGLO2*-, *PFDEF*- (*P. floridana DEFICIENS*), *PFTM6*- (*P. floridana TOMATO MADS-BOX GENE* 6), and *PFAG* (*P. floridana AGAMOUS*)-pGADT7. Each mutated CArG-box-pAbAi fusion construct was generated using the same strategy.

### Electrophoretic mobility shift (EMSA) assay

The full-length ORFs of *DOLL1*, *PFGLO2*, and *PFCRC* were cloned into the pCold TF DNA vector (Takara, Japan) using *Nde*I and *Xba*I enzyme sites (Supplementary Table S1), and each in-frame construct was transformed into *E. coli* Transetta (DE3) chemically competent cells (TransGen, Beijing, China). HIS-tag fusion protein induction was done with 0.5 mM IPTG at 15 ℃ and shaken for 24 h following the pCold™ TF DNA product manual. HIS-tag fusion proteins were extracted by using the MagneHis^TM^ Protein Purification System according to the manufacturer’s procedures (Promega, Madison, USA).

Probes including WT sequences containing each of the CArG-box or YABBY binding motifs with 25 flanking bases in each end or the indicated mutated versions, were obtained using the same strategy as in the Y1H assay (Supplementary Table S1), and were labelled using a Biotin 3’ End DNA Labelling Kit (Pierce, Rockford, USA). Unlabelled oligonucleotides of the same sequence were used as competitors. A 50 ng aliquot of each purified HIS-binding protein and 0.2 μmol biotin-labelled probes were used for the binding reaction for each sample. Samples were transferred onto a Biodyne B nylon membrane (Pierce, Rockford, USA). Fluorescence of biotin-labelled DNA was detected using the LightShift^®^ Chemiluminescent EMSA Kit (Pierce, Rockford, USA). A hybrid nylon membrane was exposed to the CCD camera of a chemiluminescence imaging analyser Celvin S 420 (Biostep, Germany), and related images were captured and analysed by the Snap And Go software (Biostep, Germany).

### Transient expression assay using a dual-luciferase system

The dual-luciferase assay was performed following a previously described method (Hellens *et al.*, 2005). Each effector plasmid was constructed with the Super1300-GFP vector using *Xba*I and *Kpn*I enzyme sites (Supplementary Table S1). The promoter sequences of WT *PFCRC*, *DOLL1*, and *PFGLO2* were amplified and cloned into the pGreen II 0800-LUC vector. Mutated versions were amplified using a related WT vector as the template by multiple routine PCR. Each combination of obtained reporter and effector plasmids was co-transformed into protoplasts of one-month old tissue-cultured seedlings in half-strength MS medium. Protoplast preparation of *Physalis* seedlings and transient expression assays were performed as previously described, with a few minor modifications (Yoo *et al.*, 2007). Approximately 10 ml of enzyme solution, including 20 mM MES (pH 5.7), 1.3% (w/v) cellulase R10, 0.3% (w/v) macerozyme R10, 0.4 mM mannitol, and 20 mM KCl, and 40% (w/v) PEG4000 was used in the PEG-calcium transfection solution. The luciferase activity was measured using the Dual-luciferase Reporter Assay System according to the manufacturer’s instructions (Promega, Madison, USA). The relative luciferase activity was calculated as the ratio between the firefly luciferase and the control *Renilla* luciferase activity. At least three independent biological replicates were measured for each sample.

### Chromatin immunoprecipitation (ChIP)-qPCR assay

Young leaves from the Super::GFP and Super::PFCRC-GFP overexpression transgenic *Physalis* plants were collected and fixed in 1 × PBS buffer containing 1% formaldehyde under vacuum for 15 min. Approximately 1.5 g of tissues was ground in liquid nitrogen and nuclei were isolated by filtering with two layers of Miracloth (Merck, Billerica, USA); chromatin fragments were prepared by sonication. After sonication, a 1/20 sample was taken out as DNA input. The remaining samples underwent immunoprecipitation. GFP-tagged proteins together with the associated DNAs were immunoprecipitated by using Pierce Protein G Magnetic Beads (ThermoFisher, Rockford, USA) coated with monoclonal anti-GFP antibody (Roche, Mannheim, Germany) at 4 °C for 2 h. Beads were washed two times with the immunoprecipitation buffer followed by two washes with TE buffer. Reverse crosslinking was done by boiling the beads at 65 °C for 12 h in the presence of 1% SDS, followed by Proteinase K treatment at 45 °C for 1 h. DNA was ethanol precipitated following phenol/chloroform extraction. qPCR was performed using SYBR^®^ Premix Ex Taq^TM^ (TaKaRa, Japan) with the gene-specific primers (Supplementary Table S1).

### *Cis*-element prediction and phylogenetic analyses

Multiple sequence alignment (MSA) was performed by BioEdit software version 5.09. The maximum likelihood (ML) phylogenetic tree was constructed by MEGA X software (Kumar *et al.*, 2018) with parameters of the Hasegawa-Kishino-Yano model gamma distributed with invariant sites (G+I), using all sites and 1000 bootstrap replicates. C*is*-element predictions were performed using PlantPan 3.0 (The Plant Promoter Analysis Navigator) online program (Chow *et al.*, 2019).

### Molecular isolation, sequencing analyses, and primer synthesis

The genomic sequence of *PFCRC*, putative promoter sequences of *DOLL1*, *PFGLO2* and *PFCRC* were isolated from genomic DNA of leaves. The corresponding cDNAs were isolated by using RT-PCR in floral tissues. The amplified DNA fragments were cloned to the vector using a pEASY^®^-Blunt Cloning kit (TransGen Biotech, Beijing, China) and were used as the templates for sequencing and subsequent synthesis of constructs. All resultant constructs were commercially sequenced, and all primers (Supplementary Table S1) were synthesized by Taihe Biotech (Beijing, China).

### Statistical analysis

Unless specifically noted, statistical analysis was performed by using IBM SPSS Statistics for Windows, Version 24.0 (IBM Corp, NY, USA).

## Results

### Floral and carpel variations of *doll1* mutants

**Fig. 1.**
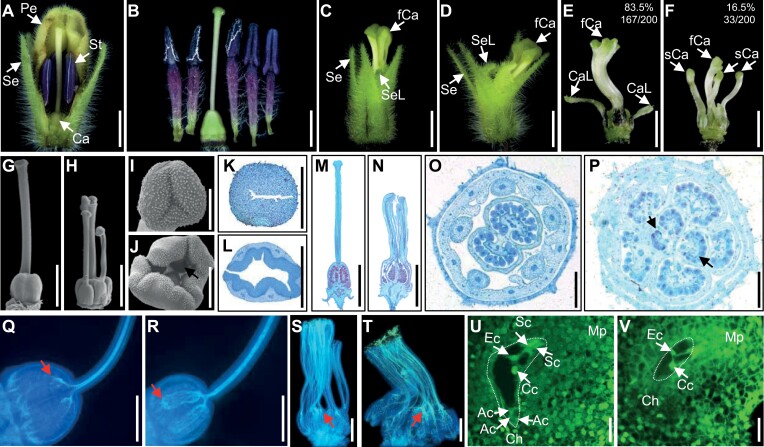
Floral and pistil morphology, structure, and functionalities of *doll1* mutants. (A) Floral bud of the wild type (WT). Partial sepal and petal tissues were removed to show the internal organs. (B) The separated internal organs (five stamens and one carpel) of WT. (C, D) Floral buds of the *doll1* mutant. No petals or stamens. (E, F) Various morphologies of the transformed carpels in the *doll1* mutant. The occurrence rate for each type was evaluated based on 200 flowers. Ca, carpel; CaL, carpel-like organs; fCa, fused carpel; Pe, petal; sCa, separated carpel; Se, sepal; SeL, sepal-like organs; St, stamen. (G, H) Pistil morphology between WT (G) and *doll1* mutant (H) in the SEM assay. (I, J) Stigma morphology between WT (I) and *doll1* (J) in the SEM assay. Black arrow, native stigma of *doll1*. (K, L) Stigma cross section between WT (K) and *doll1* (L) in the paraffin section assay. (M, N) Pistil longitudinal section between WT (M) and *doll1* (N) in the paraffin section assay. (O, P) Ovary cross section between WT (O) and *doll1* (P) by paraffin section assay. Black arrows, native ovaries of *doll1*. (Q, R) Pollen tube growth in WT pistils at 24 and 48 h after pollination. (S, T) Pollen tube growth in *doll1* pistils was retarded at 24 and 48 h after pollination with WT pollen. Red arrows indicate the positions of pollen tube elongation near the native ovaries at the time observed. (U) Mature embryo sac stage, including three antipodal cells, two synergids, one egg, and a central cell. (V) Abnormal native embryo sac development in *doll1* at the mature stage. Ac, antipodal cell; Cc, central cell; Ch, chalazal end; Ec, egg cell; Mp, micropyle end; Sc, synergid cell. Dotted lines indicate putative embryo sac profiles. White arrows indicate the positions of organs, cells or nuclei. Bars =2 mm in (A–F), (G), (H), (M) and (N); 500 μm in (I–L), and (O–T); 20 μm in (U) and (V).

Compared with the WT *Physalis floridana* flower (Fig. 1A, B), the *double-layered-lantern1* (*doll1*) mutant displays homeotic transformation of the corolla and androecium into the calyx and gynoecium, respectively (Fig. 1C, D; Zhang *et al.*, 2014a). The native pistil in *doll1* is tightly surrounded by five transformed carpels with different degrees of fusion (Fig. 1C–F), and the majority are fused to form a columnar structure (Fig. 1E). In addition, we found that some carpel-like structures existed outside of the fused pistil; they displayed a green stigma, long or short style, and a cystic structure at the bottom without ovules (Fig. 1E, F). We inferred that these structures might have originated from the stapet organ at the base of the filament.

We also observed that the *doll1* floral bud was small relative to the WT (Supplementary Fig. S1A–D), suggesting a role for *DOLL1* in controlling organ size. Another cause for reduction in floral size might be lack of corolla organs (Supplementary Fig. S1D, E). Moreover, unlike WT (Supplementary Fig. S1C), no self-fertilization occurred in *doll1*, and no fruit was obtained (Supplementary Fig. S1F). When pollinated with WT pollen, a few hybrid fruits could be harvested with small double-layered lanterns (Supplementary Fig. S1F). The abortive mutants were therefore preserved in the hybrid form, and the reason for the poor fertilization was further investigated.

### Extremely low fruit setting rate of *doll1* mutants

To precisely evaluate the fruit setting rate, normal and erected artificial pollination strategies were performed (Supplementary Fig. S2A, B). In the WT *P. floridana*, fruit setting rate was naturally about 75%, while it could be increased to 96% by artificial pollination with any of the used strategies (Supplementary Fig. S2C). However, in the *doll1* mutant, no self-pollinated fruit was found in 500 labelled flowers, whereas the fruit set was 1.37% and 10.31%, when pollinated with WT pollen using the normal strategy and the erected strategy, respectively (Supplementary Fig. S2B, C). These results further confirmed that the pistils in *doll1* were dysfunctional.

Compared with WT, the hybrid berry rescued in *doll1* was tightly surrounded by two layers of ICS (Supplementary Fig. S3A–E), apparently displayed irregular diameters, and was larger than the WT berry (Supplementary Fig. S3F–I). Transverse sections showed that the WT berry consisted of two locules, while seven locules were observed in *doll1* (Supplementary Fig. S3J, K). Relative to the WT, seed number seemed to be decreased, and most ovules were undeveloped in *doll1* (Supplementary Fig. S3J–O). Further statistical analysis showed that most ovules developed into seeds; each WT berry contained on average about 170 seeds, while only about 20 seeds from around 60 ovules were harvested from the two native locules in *doll1*, although the total number of ovules in *doll1* was similar to that in WT (Supplementary Table S2). These data indicate the direct regulation of ovule number, development, and functionality by DOLL1, or an indirect consequence, i.e. developmental constraints or placenta abnormalities. However, fertilization or post-fertilization processes of native carpels were defective in *doll1* mutants.

### Physical and physiological defects in *doll1* pistil organs

To understand the extremely low cross-fruit setting in *doll1*, we further dissected the structure and functionality of female organs. Various morphological anatomic analyses such as light and scanning electron microscopy (SEM) revealed that compared with the WT, the pistil in *doll1* was short and stocky (Fig. 1G, H), and the stigma became broad and invaginated, and had no proper organ apical fusion (Fig. 1I–L), leading to poor development of the receptive areas. The WT style was filled with guiding tissue cells (Fig. 1M), but the native style of *doll1* was hollow (Fig. 1N). The WT ovary had two locules, while the *doll1* ovary had about seven locules (Fig. 1O, P). These morphological and structural defects were further validated by micro-CT analyses (Supplementary Fig. S4). The observed developmental and structural abnormalities could result in a significant decrease in stigma receptivity and pollen tube growth in *doll1*. Stigma receptivity in terms of enzyme activity of the peroxidase genes was reduced in *doll1* relative to WT (Supplementary Fig. S5), thus affecting pollen-stigma perception. Moreover, pollen tubes in WT pistils could elongate to the bottom of the style and reach the upper part of the ovary at 24 h after pollination (Fig. 1Q), and the tubes continued to grow until they had filled the ovary to complete the fertilization processes at 48 h after pollination (Fig. 1R). However, after artificial pollination of *doll1*, only very few pollen tubes could reach the upper part of the native ovary after 24 h, and most were twisted and knotted (Fig. 1S), and the tubes remained in the upper part of the ovary after 48 h (Fig. 1T). Thus, we inferred that pollen tube guidance by ovules in *doll1* might also be dysfunctional. To corroborate this, semi*-in vitro* pollen tube guidance experiments revealed that most pollen tubes preferred elongating towards the WT ovules, instead of the ovules from the native carpels of *doll1* (Supplementary Fig. S6). Therefore, the defects in carpel morphological structure, stigma receptivity, and ovule functionality retarded pollen-stigma perception as well as pollen tube growth and guidance in *doll1* mutants.

### Developmental defects of embryo sac in *doll1*

To reveal the defects in the ovule, we further inspected embryo sac development. The *Physalis* embryo sac was of the polygonum-type, and the developmental process was recorded at several key steps (Supplementary Fig. S7A–I). A mature embryo sac was finally well developed and included seven cells and eight nuclei, with three antipodal cells at the chalaza end, two synergids and one egg at the micropyle end, and a central cell with two polar nuclei fused (Fig. 1U). In *doll1* mutants, the developmental process of the embryo sac from the megaspore mother cell to the tetrad stages was normal (Supplementary Fig. S7J, K). However, only a few megaspores could continue to normal mitosis and form a functional embryo sac (Supplementary Fig. S7L). Most megaspore cells were no longer divided and thus eventually withered (Supplementary Fig. S7M–O), and abnormal embryo sacs were developed (Fig. 1V), demonstrating that the impaired ovule functionality in *doll1* might largely be due to developmental defects of the embryo sac.

### *PFCRC* is a putative target gene of DOLL1 in carpel development

To understand the molecular basis of the floral defects in *doll1*, a preliminary comparative transcriptomics analysis of floral buds was performed, and a total of 6421 differentially expressed genes (DEGs) were revealed (Supplementary Table S3), including 465 up-regulated and 5776 down-regulated unigenes [absolute value of log_2_ ratio (*doll1*/WT) ≥ 2.0, *P* ≤ 0.01]. In *doll1*, several *Physalis* homologs of Arabidopsis genes related to pollen tube growth and embryo sac development were mostly down-regulated (Supplementary Fig. S8A; Table S4), and the expression of stigma receptivity indicator genes encoding the class III peroxidases were also significantly reduced (*P* ≤0.01; Supplementary Fig. S8B). Notably, a YABBY transcription factor gene *PFCRC* (unigene77456, *CRC* ortholog in *A. thaliana*) was up-regulated in *doll1* floral buds (Supplementary Table S3), while quantitative reverse transcription-polymerase chain reaction (qRT–PCR) data indicated that this gene was significantly down-regulated in the developing native pistils (the fourth floral whorl) compared with the WT (*P* ≤0.01; Supplementary Fig. S8C, D). To obtain clues on the function of this gene, we performed VIGS analysis. The *PFCRC*-VIGS plant architecture and floral buds were apparently similar to those of the WT phenotype (Supplementary Figs S9; S10A, B). However, the *PFCRC*-VIGS pistils were much stockier than those of the WT, which was mainly due to the reduction in style length (Supplementary Fig. S10H). Multiple styles were fused together or were only tip separated in *PFCRC*-VIGS pistils (Supplementary Fig. S10C–G), and the mutated gynoecium was formed by multiple carpel-like structures, and ovules were gestated in each carpel (Supplementary Fig. S10E, G), suggesting a variation in the carpel meristem determinacy. The pollen tube growth was disabled to reach the ovule while being twisted and spiralled at the upper part of the ovary (Supplementary Fig. S10I–L). Corroborating these observations, *PFCRC* expression was down-regulated in *PFCRC*-VIGS flowers (Supplementary Fig. S10M). The stamens were normal (Supplementary Fig. S10B); however, the self-fruit setting rate of these *PFCRC*-VIGS flowers decreased to zero (Supplementary Fig. S10N). Except for the carpel meristem determinacy, the gynoecium variations in a way resembled the observations in *doll1*, suggesting that *PFCRC* might be a target gene of DOLL1 in the carpel development of *P. floridana*.

### Molecular characterization of the *PFCRC* gene

**Fig. 2.**
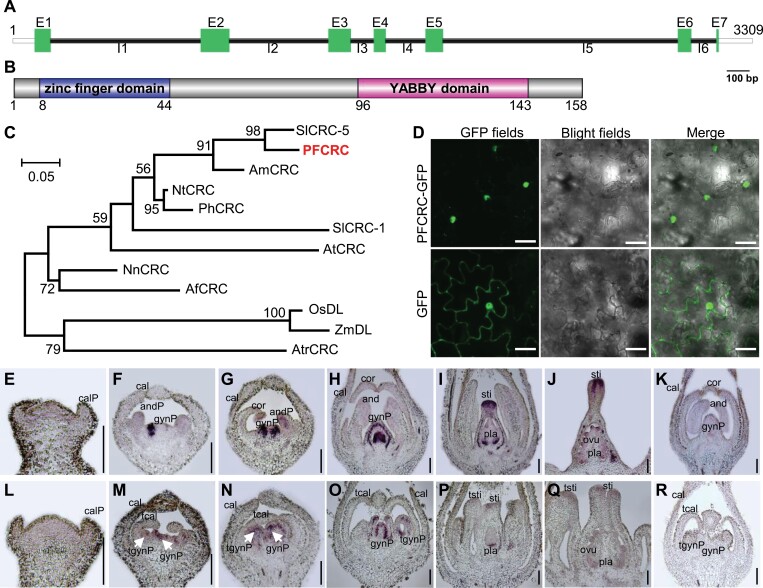
Molecular characterizations of the *PFCRC* gene. (A) Gene structure. An approximate 3.3 kb genomic sequence was amplified from genomic DNA by PCR, including seven exons (E1 to E7) and six introns (I1–I6). White rectangles at the 5’ and 3’ ends are putative 5’UTR and 3’UTR sequences; green rectangles are exons; black lines are introns. Bar =100 bp. (B) Putative PFCRC protein domains. The coding sequence encoded about 158 amino acids (aa), including a zinc finger domain (8–44 aa) and a YABBY domain (96–143 aa). (C) Phylogenetic reconstruction of CRC orthologs in various plant species. Sl, *Solanum lycopersicon*; PF, *Physalis floridana*; Am, *Antirrhinum majus*; Nt, *Nicotiana tabacum*; Ph, *Petunia x hybrida*; At, *Arabidopsis thaliana*; Nn, *Nelumbo nucifera*; Af, *Aquilegia formosa*; Os, *Oryza sativa*; Zm, *Zea mays*; Atr, *Amborella trichopoda*. OsDL or ZmDL, DROPPING LEAF in *Oryza sativa* or *Zea mays* species. The accession numbers of those genes are included in Supplementary Table S6. (D) Sub-cellular localization of PFCRC-GFP in *P. floridana* leaf epidermal cells. GFP indicates the GFP signals from empty vector controls. Bars =200 μm. (E–R) Fine floral expression of *PFCRC* revealed by *in situ* hybridization. cal, calyx; calP, calyx primordia; cor, corolla; and, androecium; andP, androecium primordia; gyn, gynoecium; gynP, gynoecium primordia; sti, stigma; ovu, ovule; pla, placenta; tcal, transformed calyx; tgyn, transformed gynoecium; tgynP, transformed gynoecium primordia; tsti, transformed stigma. Antisense nucleotide probe in WT (E–J) and *doll1* mutants (L-Q); sense nucleotide probe (K) and (R). Bars =100 μm.

To characterize *PFCRC*, PCRs were first performed based on the unigene77456 sequence. The obtained 3309 bp genomic DNA included seven exons and six introns, while its open reading frame (ORF) was 474 bp encoding 158 amino acids (Fig. 2A, B). An additional copy was found but this was apparently a pseudogene (Supplementary Fig. S11), suggesting *PFCRC* is the unique functional *CRC* ortholog. The putative PFCRC protein shared high sequence identity with CRC orthologs that have been well defined in other species (Fig. 2C; Supplementary Fig. S12). PFCRC is a putative transcription factor defined by the presence of a conserved zinc finger domain and a YABBY domain (Fig. 2B; Supplementary Fig. S12). When a PFCRC-GFP fusion construct was expressed into tobacco or *Physalis* leaf epidermal cells, expression signals mainly gathered in the nuclei (Fig. 2D; Supplementary Fig. S13A). qRT–PCR showed that *PFCRC* seemed to be specifically expressed in floral organs and was predominant in the pistils, increasing in expression along with their development; however, the expression rapidly disappeared after fertilization (Supplementary Fig. S13B, C). In accordance with this, *in situ* hybridizations demonstrated that this gene was predominantly expressed in the carpel meristem at first and then later in the ovaries, while much less expression was detected in other floral organs (Fig. 2E–J). Moreover, *PFCRC* expression was obviously down-regulated in the *doll1* pistils but detected in the transformed carpel organs (Fig. 2L–Q). No expression signal was detected, even in the tissues with a predominant *PFCRC* expression, when using sense probes (Fig. 2K, R). These observations support the hypothesis of a major role of *PFCRC* in carpel development.

### *PFCRC* is essential for carpel formation

**Fig. 3.**
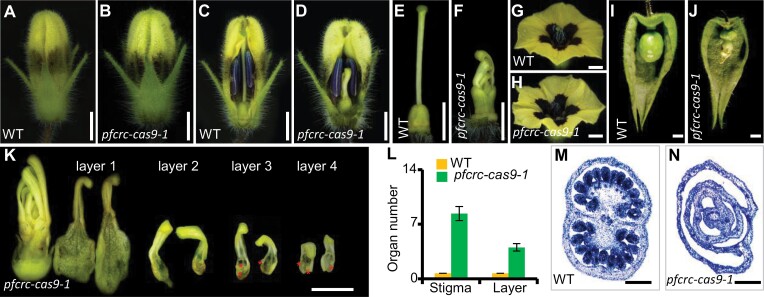
Floral variation of *PFCRC*-CRISPR/Cas9 transgenic *P. floridana* plants. (A, B) Whole floral buds of WT and *pfcrc-cas9-1*. (C, D) Floral buds of WT and *pfcrc-cas9-1*. Partial sepal and petal tissues were removed to show the internal organs. (E, F) Pistil morphology of WT and *pfcrc-cas9-1*. (G, H) Flowers in the blooming stage of WT and *pfcrc-cas9-1*. (I, J) Fruit stage of WT and *pfcrc-cas9-1*. (K) Anatomy of *pfcrc-cas9-1* gynoecium; the opened and dorsiventral carpels were separated from the outside to the inside, indicated as layer 1, 2, 3, 4 . Red stars indicate naked ovules. (L) Statistics of stigma numbers and carpel layers in WT and *pfcrc-ca9-1*. (M, N) Cross-section of ovaries of WT and *pfcrc-cas9-1*. Bars =2 mm in (A–K); 200 μm in (M) and (N).

To better reveal its developmental role, we exploited the clustered regularly interspaced short palindromic repeats/CRISPR-associated protein 9 (CRISPR/Cas9) technology to generate *PFCRC*-edited transgenic *Physalis* plants, and eight independent transgenic lines abolishing PFCRC function were obtained (Supplementary Fig. S14). We used *pfcrc-cas9-1* as a representative line in the following analyses. The morphology of floral buds of *pfcrc-cas9-1* was apparently similar to the WT (Fig. 3A, B). However, pistil alterations were found when the calyx and corolla were removed (Fig. 3C–F). Unlike the WT (Fig. 3E), pistil organs in *pfcrc-cas9-1* mutants displayed the phenotypes of stocky carpel and determinacy loss (Fig. 3F). The long WT style made the stigma parallel with the stamens (Fig. 3G), likely facilitating pollination and fertilization processes. However, pistils and stigmas in *pfcrc-cas9-1* were hidden behind stamens and were invisible (Fig. 3H). The calyx of a few *pfcrc-cas9-1* flowers could be occasionally enlarged to form an ICS, but no berry was developed (Fig. 3I, J). The berry was not produced even after artificial pollination with self-pollen that appeared normal (Supplementary Fig. S15). Corroborating this result, placing *pfcrc-cas9-1* pollen on WT stigmas generally resulted in normal fruits (Supplementary Table S5). Detailed anatomy of the pistil organ revealed that more than 11 opened and dorsiventral carpels in terms of visible stigmas grew spirally along an elongated central axis in *pfcrc-cas9-1* flowers (Fig. 3K–N), suggesting indeterminate growth. Only a few ovules (0–3) resided in the adaxial margins of these opened carpels (Fig. 3K). These floral variations in loss-of-function mutants indicated that *PFCRC* exerted roles in determining the carpel meristem determinacy, carpel closure, functionality, and ovule number.

There were no detectable phenotypic changes in the F_1_ generation after cross-pollinating WT with *pfcrc-cas9-1* pollen. In 209 self-F_2_ plants obtained, 158 plants showed WT floral morphology, and 51 plants displayed *pfcrc* phenotypes, conforming to a 3:1 segregation ratio (Supplementary Fig. S16), suggesting that the floral variation in *pfcrc-cas9-1* resulted from a single and recessive gene mutation. In line with this, the three *PFCRC*-related genotypes conformed to a 1:2:1 segregation ratio and were linked with the phenotypic variations (Supplementary Fig. S16). Single mutation in the *PFCRC* gene could significantly change the pistil morphology and functionality, and the resultant gynoecium structure was fully opened, thus implicating a role of the *CRC* gene in the origin of the carpel.

### Genetic interactions of *PFCRC* and *DOLL1* in carpel development

**Fig. 4.**
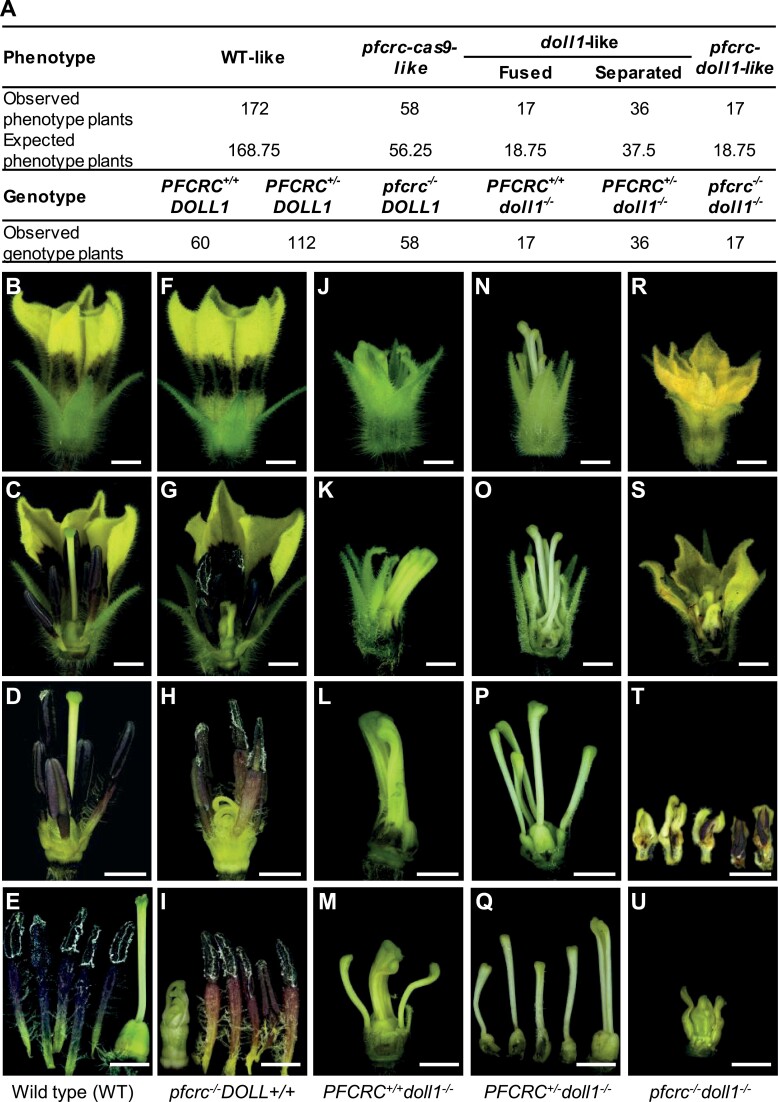
Segregation in F_2_ populations of *pfcrc-cas9-1* ♂ × *doll1* ♀. (A) Statistics of phenotypic and genetic variation. (B-U) Floral variation. (B–E) WT (wild type, *PFCRC*^*+/+*^*DOLL1*^*+/+*^) floral organ morphology. (F–I) *pfcrc*^*-/-*^*DOLL1*^*+/+*^ floral organ morphology. (J-M) *PFCRC*^*+/+*^*doll1*^*-/-*^ floral organ morphology. (N–Q) *PFCRC*^*+/-*^*doll1*^*-/-*^ mutant floral organ morphology. (R–U) *pfcrc*^*-/-*^*doll1*^*-/-*^ mutant floral organ morphology. Bars =2 mm.

To reveal the genetic interaction between *PFCRC* and *DOLL1*, cross-pollination was performed using *pfcrc-cas9-1* as the donor and *doll1* as the recipient. Ten hybrid fruits were ultimately obtained (Supplementary Table S5). After F_1_ self-crossing, we performed genotypic and phenotypic analyses of the 300 F_2_ plants (Fig. 4A; Supplementary Fig. S17). Four floral phenotypes were produced, WT-like (Fig. 4B–E), *pfcrc-cas9*-like (Fig. 4F–I), *doll1*-like (Fig. 4J–Q), and the expected *pfcrc-doll1* double mutant-like plants (Fig. 4R–U). The separation of phenotypic variation basically conformed to a 9:3:3:1 ratio (Fig. 4A). However, there were genetic interactions in carpel development. First, 17 *doll1*-like plants displayed a fused pistil phenotype (Fig. 4J–M), and these were homozygous *doll1* mutants without *PFCRC* being edited, named *PFCRC*^+/+^*doll1*^-/-^ (Supplementary Fig. S17). However, the remaining 36 *doll1*-like plants showed that the transformed pistil organs were separated from the original pistil (Fig. 4N–Q), and they were homozygous for the *doll1* locus and heterozygous in the edited *pfcrc* locus, named *PFCRC*^*+/-*^*doll1*^*-/-*^ (Supplementary Fig. S17). These data suggest that a possible dosage effect of *PFCRC* in *doll1* mutant background might exist for carpel organ development, or *DOLL1* was negatively epistatic to *PFCRC* in determining the fusion of the inner two transformed pistils. However, the transformed pistil structure from the original pistil in the *pfcrc*^*-/-*^*doll1*^*-/-*^ flowers resembled that in *pfcrc-cas9-1* flowers, but it was smaller in size, suggesting an additive effect of these two genes in carpel identity and organ size control.

**Fig. 5.**
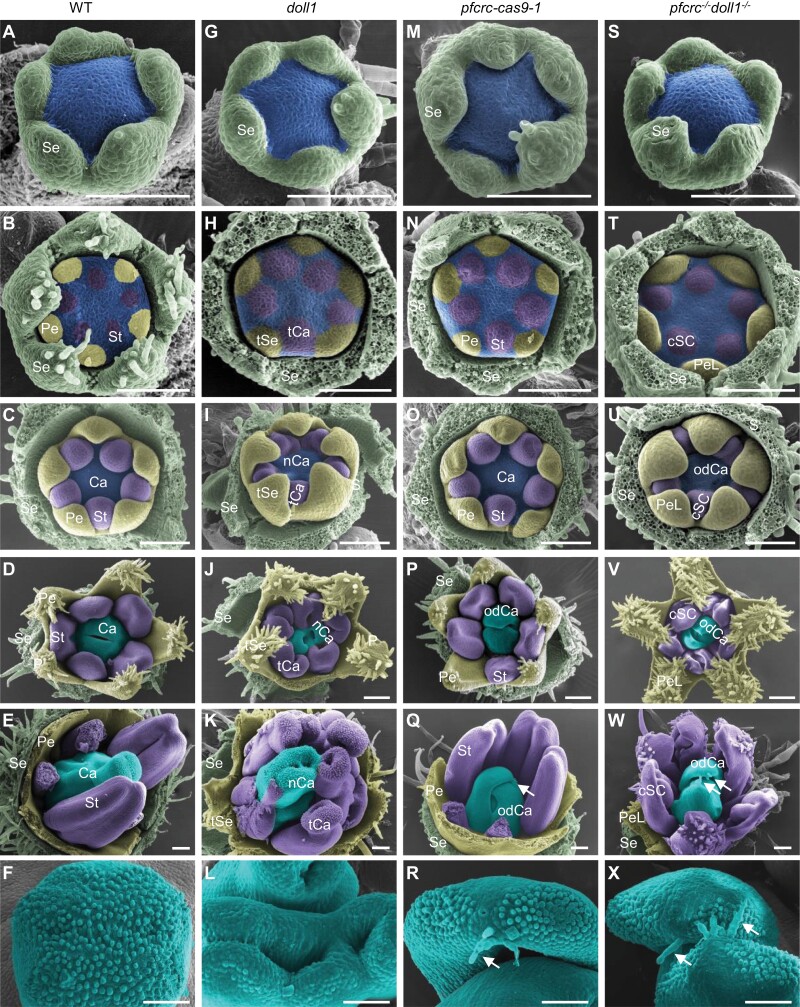
SEM analysis of *Physalis* floral organogenesis. (A–F) Floral organogenesis of the WT. *Physalis* floral organogenesis includes stages of sepal (A), petal (B), stamen (C), carpel (D, E) primordia initiation and organ formation. (F) WT stigma cell morphology. Ca, carpel primordia; Pe, petal primordia; Se, sepal primordia; St, stamen primordia. (G–L) Floral organogenesis of *doll1* mutant. tSe, transformed sepal from the second whorl; tCa, transformed carpel from the third whorl; nCa, the native carpel in the fourth whorl. (M–R) Floral organogenesis of *pfcrc-cas9-1* mutant. odCa, opened and dorsiventral carpel. White arrows, epidermal hair structure on the odCa tip. (S–X) Floral organogenesis of *pfcrc*^*-/-*^*doll1*^*-/-*^ double mutant. PeL, petal-like organ in the second whorl; cSC, chimera of stamen- and carpel-like organ in the third whorl. White arrows, epidermal hair structure on the tip of odCa in *pfcrc*^*-/-*^*doll1*^*-/-*^ mutants. Some organs were removed to better show the carpels. Bars =100 μm.

To address the organ identity alteration, we compared the early floral organogenesis of WT, *doll1*, *pfcrc-cas9-1*, and *pfcrc*^-/-^*doll1*^-/-^ via SEM analyses. Six stages were investigated from floral meristem (FM) initiation to organ primordium formation of four floral whorls; meristem morphology and early floral development were similar among these plants (Fig. 5; Supplementary Fig. S18). In WT plants, floral organ differentiation started with the development of five sepal primordia from the flank of the FM (Fig. 5A; Supplementary Fig. S18), and other floral organ primordia were developed successively from the inner meristematic cells, for five petals, five stamens, and two carpels (Fig. 5B–E). A fully developed pistil was formed with an enlarged ovary, a slender style, and a stigma covered with mastoid cells (Fig. 5F). Compared with WT, primordia of floral whorl 3 in the *doll1* mutant became carpel-like organs surrounding the native carpel primordia (Fig. 5G–L). The *doll1* stigma cell morphology of all the carpel-like organs was the same as that in WT (Fig. 5L). In *pfcrc-cas9-1* plants, primordia of the outer three floral whorls were developed the same as the WT (Fig. 5M–R). However, carpel primordia were developed into opened carpels that had undergone differential growth rate; thus, the smaller one was covered by the larger one (Fig. 5R). In contrast to WT and *doll1*, the epidermal hair cells (trichomes) were initiated on the apex of the opened carpels in the *pfcrc* mutants (Fig. 5R). Meristem morphology and early floral development were however similar to WT (Fig. 5S-V) , and the chimera of corolla and stamen were observed in the floral whorl 3 of *pfcrc*^*-/-*^*doll1*^*-/-*^ mutants (Fig. 5W). Opened carpels of *pfcrc*^*-/-*^*doll1*^*-/-*^ mutants also had trichomes at their tips (Fig. 5X). These characteristics resembled the vegetative growth observed on sepals/petals, a possible indicator of organ identity alteration. Nonetheless, the opened carpels of *pfcrc*^*-/-*^-related mutants had adaxial surfaces different from the abaxial surface, but the abaxial surface of the opened carpels of the *pfcrc*^*-/-*^*doll1*^*-/-*^ mutants was similar to that of *pfcrc-cas9-1*, *doll1*, and WT (Supplementary Fig. S19). Therefore, *PFCRC* may be involved in carpel organ identity specification.

### Negative epistasis of *PFCRC* and *DOLL1* for corolla and stamen organ identity

**Fig. 6.**
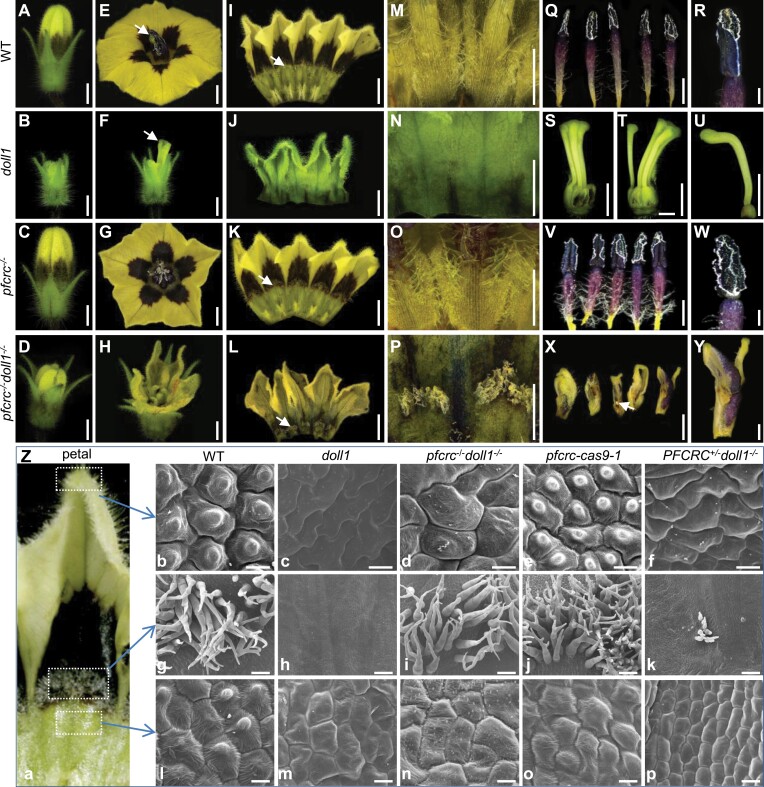
Phenotypic variation of *pfcrc*^*-/-*^*doll1*^*-/-*^ in the second and third floral whorls. (A–D) Floral buds of the WT, *doll1*, *pfcrc*^*-/-*^, and *pfcrc*^*-/-*^*doll1*^*-/-*^ double mutant. (E–H) Flowers of the WT, *doll1*, *pfcrc*^*-/-*^, and *pfcrc*^*-/-*^*doll1*^*-/-*^ double mutant. White arrows in (E) and (F) indicate stigmas. (I–L) Floral whorl 2 organ morphology of the WT (petal), *doll1* (sepaloid organ), *pfcrc*^*-/-*^ (petal), and *pfcrc*^*-/-*^*doll1*^*-/-*^ double mutant (petal-like organ). White arrows show epidermal hair-like structures. (M–P) Morphology of floral whorl 2 base area of the WT, *doll1*, *pfcrc*^*-/-*^, and *pfcrc*^*-/-*^*doll1*^*-/-*^ double mutant. (Q–Y) Morphology of floral whorl 3 of the WT, *doll1*, *pfcrc*^*-/-*^, and *pfcrc*^*-/-*^*doll1*^*-/-*^ double mutant. The white arrow in (X) indicates the ovule. Bars =2 mm. (Z) Cytological morphology of petal epidermal cells. (a) A petal. The sections for SEM are boxed. (b–f) Cell morphology in the tip area of sepals. (g–k) Cytological morphology of epidermal hair-like structures in the middle area of the sepals. (l–p) Cell morphology near to the hair-like areas. The genetic background is indicated. Bars =10 μm in (b–f); 100 μm in (g–k); 20 μm in (l–p).

Unlike *doll1*, normal corolla and stamen-like structures were also seen in the *pfcrc*^*-/-*^*doll1*^*-/-*^ double mutant plants (Fig. 4). To better understand this, we further compared the floral variations of the mentioned floral whorls among these genotypes. Floral buds in the *pfcrc*^*-/-*^*doll1*^*-/-*^ and *pfcrc*^*-/-*^ mutants were larger than those in *doll1*, and they displayed some corolla characteristics (Fig. 6A–D). Like WT and *pfcrc*^*-/-*^ flowers, a yellow and corolla-like structure was observed in the double mutants at the blooming stage (Fig. 6E–L). Five purple spots were present, and five stapets adhered to the bottom of WT and *pfcrc*^*-/-*^ corollas; epidermal hair structures were scattered at the adaxial surface (Fig. 6M–P), while the second floral whorl was a calyx in *doll1*, and the adaxial surface was smooth (Fig. 6B, F, J, N). However, in *pfcrc*^*-/-*^*doll1*^*-/-*^ double mutants, blurry purple spots, and epidermal hair structures on the adaxial surface of the second floral whorl were restored to WT form (Fig. 6L, P). In addition, the third floral whorl of *pfcrc*^*-/-*^*doll1*^*-/-*^ double mutants displayed huge divergence from WT (stamens), *doll1* (carpel-like), and *pfcrc*^*-/-*^ (Fig. 6R–Y). Few ovules could be seen at the base of these transformed organs, and they tended to be morphologically restored into WT-like stamens in terms of the deep purple colour (Fig. 6X, Y), further implying that PFCRC has a C-function to determine carpel identity. SEM analyses revealed that epidermal cell morphology of the corolla-like structure in *pfcrc*^*-/-*^*doll1*^*-/-*^ was similar to that of WT corolla (Fig. 6Z), while the restored stamen-like structures were chimeras of pistils and stamens in the double mutants (Supplementary Fig. S19). These results suggest negative epistasis of *PFCRC* and *DOLL1,* and that such genetic interaction is involved in specifying the second and third whorls of floral organ identity.

### Overexpressing *PFCRC* partially restores *doll1*

To obtain further functional clues, *PFCRC*-overexpression (*PFCRC*-OE) transgenic plants were generated. Three independent *PFCRC*-OE lines (L1, L2, and L3) were produced (Supplementary Fig. S20A, B). Visible floral organs in these *PFCRC*-OE plants became larger than those of WT (Supplementary Fig. S20C–P), and ovules and seeds were also enlarged (Supplementary Fig. S20Q–V), further indicating that PFCRC could regulate floral organ size. However, mature berry weight was reduced and was correlated with the reduction in seed number (Supplementary Fig. S20W, X). Around 150 ovules per ovary were gestated on average, and nearly all were fertilized to become mature seeds in WT (Supplementary Fig. S20S, W), while in *PFCRC*-OE plants, there were around 130 ovules in one ovary, but only about 30% could be fertilized and develop to mature seeds (Supplementary Fig. S20T, W). The self-fruit setting rate of *PFCRC*-OE transgenic lines was 33%, which was lower than that of the WT (Supplementary Table S5), while the pollen maturation was normal, albeit larger than WT pollen (Supplementary Fig. S15), hinting that PFCRC might also be involved in establishment of cross-compatibility, or overexpressing *PFCRC* might affect pollen development, thus leading to uncharacterized pollen defects. These hypotheses were further verified by enormous efforts putting *PFCRC*-OE pollen on stigmas of various genotypes, and fewer or no hybrid seeds were obtained under all such conditions (Supplementary Table S5).

To observe the effect of overexpressing *PFCRC* in a *doll1* background, we therefore generated three independent transgenic lines via overexpressing *PFCRC* cDNA into *doll1* heterozygous plants. In the T_2_ generations, *doll1*-like plants were isolated, and genotypic analyses indicated high *PFCRC* and null *DOLL1* expression in their flowers. The plants were designated *PFCRC*-OE-*doll1* (Supplementary Fig. S21A). Compared with *doll1*, floral organs were larger in these *PFCRC*-OE-*doll1* flowers (Supplementary Fig. S21B–G). Moreover, the ovule number in the native carpels of *PFCRC*-OE-*doll1* was significantly increased (*P* =0.0012; Supplementary Fig. S21H–K). After being cross-pollinated with WT pollen, fruit/seed setting rate and seed number of the native carpels were also increased compared with *doll1* (Supplementary Fig. S21K). Therefore, overexpressing *PFCRC* did enlarge organ size and was able to partially complement *doll1* female fertility defects, thus further supporting the regulatory and genetic interactions of *DOLL1* and *PFCRC* in carpel development.

### Molecular clues for genetic regulation between *DOLL1* and *PFCRC*

**Fig. 7.**
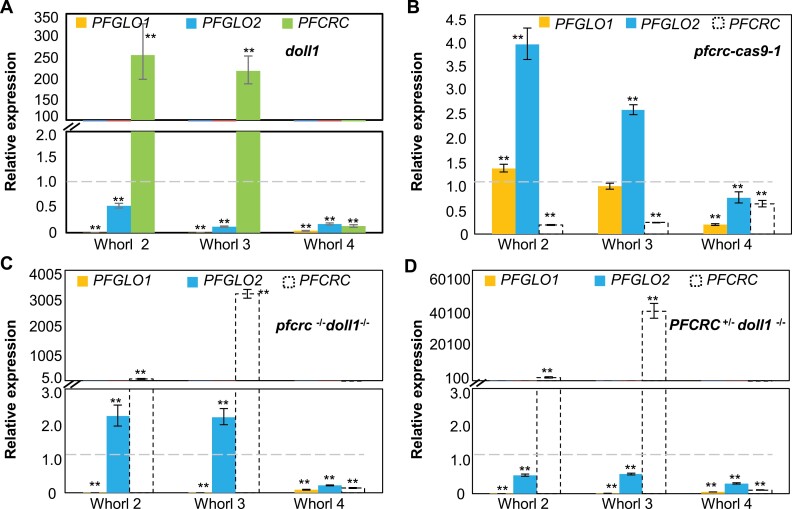
Floral expression of *DOLL1 (PFGLO1)*, *PFGLO2* and *PFCRC* in different genetic backgrounds of *P. floridana*. (A) Gene expression in the *doll1* mutant. (B) Gene expression in *pfcrc-cas9-1* plants. (C) Gene expression in the *pfcrc*^*-/-*^*doll1*^*-/-*^ double mutant. (D) Gene expression in the *PFCRC*^*+/-*^*doll1*^*-/-*^ double mutant. Relative expression is indicated by fold changes relative to the WT petal, stamen, and carpel organs. White dotted columns in (B-D), the edited *pfcrc* mRNA; dotted lines in (A-D), relative expression of these three genes in WT organs set as 1.0. The *PFACTIN* was used as the internal reference gene. **, Student’s *t*-test, *P*<0.01.

To understand the molecular basis of *DOLL1* and *PFCRC* interactions, we first examined their gene expression in various genetic backgrounds (Fig. 7; Supplementary Figs S22, S23). In WT, both *DOLL1* (also named *PFGLO1*) and its paralog *PFGLO2* are highly expressed in the corolla and androecium (Zhang *et al.*, 2014a), and expressed in the carpel primordia and gynoecium (Supplementary Fig. S22), supporting their roles in carpel development. *PFCRC* expression was down-regulated in *doll1* carpels (whorl 4) but predominantly up-regulated in the second and third floral whorls (Fig. 7A). In *pfcrc-cas9-1* plants, only *PFGLO2* expression was obviously up-regulated in the second and third floral whorls, while both *DOLL1* and *PFGLO2* were seemingly down-regulated in carpels (Fig. 7B). Expression of the edited *pfcrc* mRNA was also down-regulated in all floral whorls detected (Fig. 7B), and was significantly up-regulated in the second and third floral whorls, but was seriously repressed in the fourth floral whorl in the double mutants *pfcrc*^*-/-*^*doll1*^*-/-*^ and *PFCRC*^*+/-*^*doll1*^*-/-*^(Fig. 7C, D). Furthermore, *PFGLO2* expression was up-regulated in the *pfcrc*^*-/-*^*doll1*^*-/-*^ background (Fig. 7C), while it was repressed in the *PFCRC*^*+/-*^*doll1*^*-/-*^ mutants (Fig. 7D). Similar variations of gene expression were observed when an independent reference gene was used (Supplementary Fig. S23A–D). However, ectopic expression of *PFCRC* could repress *PFGLO2* in any of the floral whorls, and this did not affect *DOLL1* expression (Supplementary Fig. S23E, F). These results suggested that *PFCRC* and *P. floridana GLOBOSA*-like genes (*PFGLOs*) might be mutually regulated.

### Regulation of *PFCRC* by PFGLOs

**Fig. 8.**
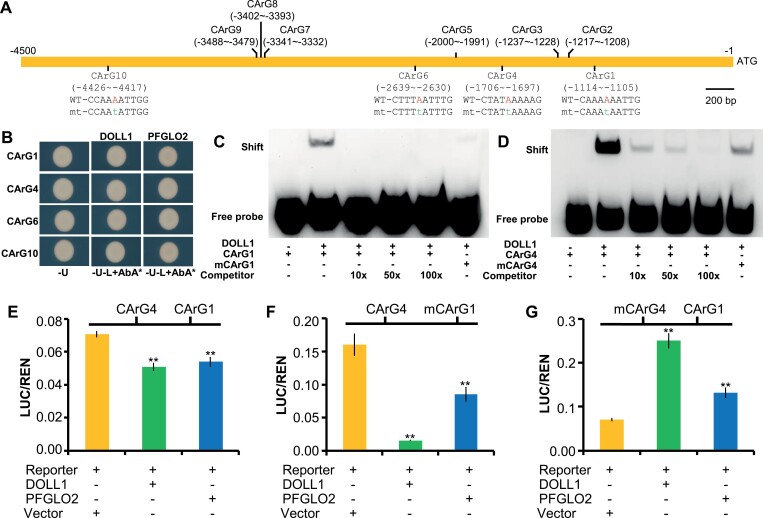
DOLL1 regulates *PFCRC* by binding to its CArG-box motifs. (A) Schematic diagram showing 10 putative CArG-box motifs in the *PFCRC* promoter upstream of the start codon (ATG). Numbers represent the position of each CArG-box. WT, wild type; mt, mutated CArG-box, in which the fifth nucleotide acid A was substituted with t, highlighted in red and green, respectively. (B) DOLL1 and PFGLO2 bind to the CArG1, CArG4, CArG6 and CArG10 in the Y1H assay. -U, -U-L, SD/-Ura and SD/-Ura-Leu solid medium; *, Aureobasidin A concentration used in Y1H for CArG1, CArG4, CArG6, and CArG10 was 400 ng ml^-1^, 800 ng ml^-1^, 300 ng ml^-1^, and 700 ng ml^-1^, respectively. (C, D) HIS-DOLL1 fusion proteins bind to CArG1 and CArG4 in the EMSA assay. + indicates the presence; –, the absence of corresponding components as indicated; mCArG1 and mCArG4, mutated probes; competitor, unlabelled WT CArG-box probes. (E) DOLL1 or PFGLO2 repress *LUC* expression in the dual-luciferase assay. (F) DOLL1 or PFGLO2 repress *LUC* expression when CArG1 is mutated in the dual-luciferase assay. (G) DOLL1 or PFGLO2 activates *LUC* expression when CArG4 is mutated. The relative LUC activities were normalized to the REN activity. Vector in (E-G), the empty effector vector Super1300-GFP was added as the control. Values are means ± SD (*n*=3). **, *P*<0.01.

To find the mechanism by which DOLL1 regulated *PFCRC* expression, 6048 bp of the *PFCRC* putative promoter sequence was isolated. A yeast one-hybrid (Y1H) assay showed that DOLL1 and PFGLO2 could bind to the tested fragments of the putative *PFCRC* promoter to activate reporter gene expression (Supplementary Fig. S24). The *cis*-element prediction revealed 10 putative CArG-box motifs on the *PFCRC* promoter, named CArG1 to CArG10 (Fig. 8A). Six CArG-boxes (CArG2, CArG3, CArG5, CArG7, CArG8, and CArG9) belonged to the variant C(A/T)_8_G type (Supplementary Fig. S25A), while the other four (CArG1, CArG4, CArG6, and CArG10) shared the consensus of CHW_2_AAW_2_DG (Supplementary Fig. S25B). Only CArG10 was a classical CC(A/T)_6_GG type CArG-box (Fig. 8A). Y1H assays further showed that both DOLL1 and PFGLO2 did not activate the reporter gene expression when the reporter gene was driven by the triple tandem repeats (TTR) of each C(A/T)_8_G type motif (Supplementary Fig. S24). However, they could activate expression when the TTR of each CHW_2_AAW_2_DG type was used (Fig. 8B). These motifs were apparently required for PFGLOs binding, and the mutations in the conserved sites could abolish the transcriptional activity (Supplementary Fig. S24). In particular, the fifth and sixth adenines (A) were conserved in these functional CArG-boxes (Supplementary Fig. S25B). We then focused on functional verification of the fifth A via site-directed mutagenesis, and substituting it with a thymine (T) completely abolished the activation activity in the yeast system (Supplementary Fig. S26), suggesting that the fifth A was necessary for DOLL1 or PFGLO2 binding. These results were further confirmed in an electrophoretic mobility shift (EMSA) assay by using the HIS-DOLL1 or HIS-PFGLO2 fusion proteins. Both fusion proteins were able to bind to CArG1, CArG4, CArG6, and CArG10 (Supplementary Fig. S27A, B). Site-directed mutagenesis (the same strategy as in Y1H) and competition analyses revealed that DOLL1 and PFGLO2 failed to bind or were extremely weakly bound to the mutated versions of these CArG-boxes (Fig. 8C, D; Supplementary Figs S27C, S28), while increasing the concentration of the unlabelled WT probes resulted in completely absent or much weaker, retarded bands (Fig. 8C, D; Supplementary Fig. S28), indicating that PFGLO proteins specifically and competitively bind to the *PFCRC* promoter via these CArG-boxes.

The regulatory effect on gene expression was next evaluated using the dual-luciferase system in *P. floridana* leaf protoplasts. In this assay, the effector plasmids expressing DOLL1 or PFGLO2 full-length proteins driven by a *Super* promoter were constructed (Supplementary Fig. S25C, D). We first generated three reporter constructs, one containing the WT CArG-boxes (CArG1 and CArG4) in the 2014 bp *PFCRC* promoter fragment, and the other two containing one mutated CArG-box (mCArG1 or mCArG4; Supplementary Fig. S29). Co-expression of each effector construct with the LUC reporter construct containing the WT *PFCRC* promoter showed that *LUC* expression was significantly repressed (*P* ≤ 0.00143; Fig. 8E). However, when the reporter construct carried mCArG1, *LUC* expression was considerably down-regulated (Fig. 8F), while the opposite results were obtained when CArG4 was mutated (Fig. 8G). Therefore, CArG1 was an activator, while CArG4 was a repressor in regulating *PFCRC* via PFGLO proteins.

We next expanded the putative *PFCRC* promoter fragment to 4549 bp, covering all functional CArG-boxes. In this experiment, they were all mutated, and then each CArG-box was restored to evaluate its role, thus generating five mutated reporter constructs (Supplementary Figs S29; S30A). When either DOLL1 or PFGLO2 was co-expressed with LUC reporter driven by the native WT *PFCRC* promoter, both DOLL1 and PFGLO2 repressed *LUC* expression (Supplementary Fig. S30B). In contrast, *LUC* expression was unchanged once all four CArG-box motifs were mutated in the *PFCRC* promoter (Supplementary Fig. S30C). For the reporter construct in which CArG1 was restored to the WT, the LUC reporter gene was significantly activated (*P*≤0.0003; Supplementary Fig. S30D). However, after restoring each of the other three motifs (CArG4, CArG6, or CArG10), *LUC* expression was significantly repressed (*P*≤0.00037; Supplementary Fig. S30E–G). These results reinforced the suggestions that CArG1 exerted an activator effect and that CArG4 acted as a repressor in regulating *PFCRC* expression. In addition, CArG6 and CArG10 might also play repressor roles.

### Regulation of *PFGLOs* by PFCRC

The regulation of *PFGLO* gene expression by PFCRC was investigated using the same systems. Around 2 kb of the promoter sequences of *DOLL1* (1838 bp) and *PFGLO2* (2194 bp) were isolated, and *cis*-element prediction revealed three and two putative YABBY transcription factor binding motifs (YBM), respectively (Supplementary Fig. S31A, B). Constructs expressing full-length PFCRC proteins were used as effectors, and the isolated *DOLL1* or *PFGLO2* promoter was tested to drive the reporter gene (Supplementary Fig. S31C). Five *DOLL1*- and four *PFGLO2*-related reporter constructs were generated. Each gene-related construct included WT and mutated versions (the consensuses C or G in each YBM motif was substituted to A or T), and the mutated versions were subjected to single, double or triple motifs of the characterized YBMs (Supplementary Fig. S31A, B, D, E). Co-expressed with the effector PFCRC construct, *LUC* expression in each *DOLL1*-related reporter was unaltered (Supplementary Fig. S31D), while it was repressed in each *PFGLO2*-related reporter (Supplementary Fig. S31E). The specificity of the binding sites of PFCRC on the *PFGLO2* promoter was tested by EMSA assays. We found that HIS-PFCRC fusion proteins were able to bind to DNA probes via both YBM1 and YBM2 on the *PFGLO2* promoter, but this failed once the conserved sites were mutated (Supplementary Fig. S32A-D). To confirm these results, ChIP-qPCR assay was performed by using *Super::PFCRC-GFP* overexpression transgenic *Physalis* plants. The result showed that PFCRC proteins could bind to each putative YBM motif in the *PFGLO2* promoter *in vivo*, but failed to bind to the putative YBMs in the *DOLL1* promoter (Supplementary Fig. S32E, F). Therefore, PFCRC could directly regulate *PFGLO2* instead of *DOLL1*.

## Discussion

Functional inference of a gene in a few model species, while useful, is insufficient to understand the role of the gene in development and evolution. The functions of orthologous genes have diversified since their origin during speciation, and paralogs resulting from gene duplication have often undergone subsequent divergence during evolution. These evolutionary processes usually accompany the origin of new morphological, biochemical, and physiological traits and related diversification (He and Saedler, 2005; Sakakibara *et al.*, 2013; Rensing, 2014; Vlad *et al.*, 2014; Panchy *et al.*, 2016; Meng *et al.*, 2020). In this study, we demonstrated the functional diversification and pleiotropy of both *DOLL1* and *PFCRC* and investigated their interactions in the floral development in *Physalis*. The novel discoveries herein have implications for understanding flower and fruit evolution, particularly the origin of the carpel.

### Recruiting B-class *MADS*-box genes to target *PFCRC* for carpel development

B-class *MADS*-box genes have been demonstrated to play roles in corolla and stamen organ identity specification. A mutation in either of these genes, such as *GLOBOSA* (*GLO*)/*PISTILATA* (*PI*) or *DEFICIENS* (*DEF*)/*APETALA3* (*AP3*), produces flowers with petals transformed into sepals, and stamens converted into carpels in *Antirrhinum* and Arabidopsis (Bowman *et al.*, 1989; 1991; Sommer *et al.*, 1990; Jack *et al.*, 1992; Tröbner *et al.*, 1992). However, in some species, such as plants in the Solanaceae, these genes were duplicated, and the duplicates display functional redundancy with variable sub-functionalization processes that play roles in floral organ diversity (Vandenbussche *et al.*, 2004; de Martino *et al.*, 2006; Rijpkema *et al.*, 2006; Geuten and Irish, 2010; Zhang *et al.*, 2014a; 2015). In *Physalis*, *DOLL1* (*PFGLO1*) performs a complete B-function in petal and stamen organ specification, while *PFGLO2* mainly exerts a role in male fertility with the capacity to partially compensate the *doll1* defects (Zhang *et al.*, 2014a; 2015). The defect in the transformed carpels is easily understandable in *doll1*. However, the native carpel structure was deformed, and functionality was aborted, leading to limited fruit set in *doll1* mutants, thus reflecting the new role of DOLL1 outside of the petals and stamens in *Physalis*. This was further supported by extending the expression of both *DOLL1* and *PFGLO2* in carpel primordia and carpels, thus conferring a role for B-function genes in carpel development.

**Fig. 9.**
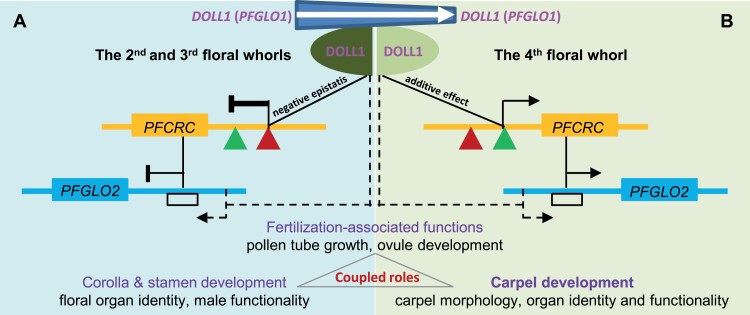
*DOLL1* and *PFCRC* interactions for floral development. (A, B) Neofunctionalization of *DOLL1*, and regulatory and genetic interactions of *PFGLOs* and *PFCRC* in a context-dependent manner for floral development. Such a neofunctionalization is fulfilled by extending the expression of *DOLL1* in the carpel following the establishment of interactions with *PFCRC*. (A) DOLL1 (also PFGLO1) acts as a repressor in the second and third floral whorls. DOLL1 represses *PFCRC* expression by binding to the CArG-box repressor (red triangle), which further represses *PFGLO2* expression. The direct activation of *PFGLO2* by DOLL1 cannot be excluded. (B) DOLL1 acts as an activator in the fourth floral whorl. DOLL1 activates *PFCRC* expression by binding to the CArG-box activator (green triangle), which could activate *PFGLO2* expression. The solid lines indicate the confirmed relations, while the dashed lines need verification.

*DOLL1* mutation affected carpel development and functionality in three coupled aspects: (i) native ovule (embryo sac) development; (ii) carpel structure, particularly stigma and style tissues, thus leading to (iii) defects in stigma receptivity and in pollen tube growth and guidance. Our preliminary transcriptome comparison between *doll1* and WT floral buds revealed substantial DEGs. Defects in fertilization-associated functionalities such as pollen-stigma perception, pollen tube growth, and pollen tube guidance were affected by many genes (Supplementary Table S4), i.e. MADS-box gene *AGL80* and *BUDDHA’S PAPER SEAL* genes in Arabidopsis (Ge *et al.*, 2017; Zhang *et al.*, 2020), and the peroxidase genes for stigma receptivity (Galen and Plowright, 1987; McInnis *et al.*, 2006). We found that their homologous genes in *doll1* mutants were mostly down-regulated, basically accounting for the related defects in *doll1*. We further found a DEG encoding *PFCRC*, the *CRC* ortholog that was responsible for carpel and nectary development in Arabidopsis (Alvarez and Smyth, 1999; Bowman and Smyth, 1999; Eshed *et al.*, 1999). *PFCRC* was predominantly expressed in developing carpels but was null in petals and stamens. In the *doll1* mutant, *PFCRC* expression was decreased in the native carpels, but was significantly activated in the transformed second and third floral whorls. Our molecular analysis confirmed that DOLL1 could directly bind to the CArG-box motifs on the *PFCRC* promoter and regulate its expression in a context-dependent manner (Fig, 9A, B). Furthermore, VIGS- and CRISPR/Cas9-mediated *PFCRC* knockdowns and gene-edited transgenic plants showed partial similarities in carpel defects, including the stocky carpels, reduced ovule numbers, and retarded pollen tube guidance of *doll1* without affecting other floral organ development, while overexpressing *PFCRC* could improve the female fertility defects in *doll1*. These results suggested that *PFCRC* confers carpel-specific traits, similar to observations in rice (Yamaguchi *et al.*, 2004; Sugiyama *et al.*, 2019). Moreover, the B-class *MADS* box gene *SUPERWOMAN 1* (*SPW1*, also known as *OSMADS16*) could be extended to the carpels when *DROOPING LEAF* (*DL*), the *CRC* orthologous gene, was deleted in rice (Nagasawa *et al.*, 2003). However, *DOLL1* floral expression was not altered in *PFCRC*-edited *Physalis* mutants, supporting its position upstream of *PFCRC* (Fig. 9A, B). Therefore, the role of DOLL1 in carpel development and functionality is partially fulfilled via recruiting *PFCRC* as one downstream target gene. In Arabidopsis, *CRC* was also demonstrated to be a direct target of AP3 and PI (Bowman and Smyth, 1999; Lee *et al.*, 2005a; Wuest *et al.*, 2012), the third/fourth whorl organs are not properly fused and deformed in *ap3* or *pi* mutants but less evident than *doll1* in *Physalis*, which is possibly due to the shortness of the style (Bowman and Smyth, 1999).

*PFGLO2*, the paralog of *DOLL1*, was also down-regulated in *doll1* mutants, and this down-regulation is partially due to loss of DOLL1 regulation (Zhang *et al.*, 2014a). However, PFGLO2 was uniquely and significantly up-regulated in the second and third floral whorls when *PFCRC* was dysfunctional, such as in *pfcrc-cas9-1* and *pfcrc*^*-/-*^*doll1*^*-/-*^ double mutants. We further showed that PFCRC could directly regulate *PFGLO2* expression, as PFCRC could bind to its binding site on the *PFGLO2* promoter to regulate gene expression (Fig. 9A, B). Moreover, compared with *doll1*, the homeotically transformed sepals and carpels tended to be restored in *pfcrc-doll1* mutants, similar to the effects observed when overexpressing *PFGLO2* in a *doll1* background (Zhang *et al.*, 2014a). However, *PFGLO2* regulation by PFCRC in flower development is dependent on DOLL1. We also found that PFGLO2 was able to bind to the *PFCRC* promoter, implying that *PFCRC* is regulated by PFGLO2. However, recruiting *PFGLO2* and its regulatory roles between *DOLL1* and *PFCRC*, particularly in carpels, needs further investigation.

Floral B-function genes during floral organogenesis are usually restricted to the second and third floral whorls (Jack *et al.*, 1992; Tröbner *et al.*, 1992; van der Krol *et al.*, 1993). However, their expression can extend to carpels in some species (Goto and Meyerowitz, 1994; Kim *et al.*, 2005; Bartlett and Specht, 2010; Mondragón-Palomino and Theissen, 2011), implying their roles outside of petals and stamens. A few examples have revealed this: for example, Arabidopsis *AP3* and *PI* confer floral determinacy (Krizek and Meyerowitz, 1996), grapevine *VvPI* represses normal fleshy fruit development (Fernandez *et al.*, 2013), rice *SPW1* involved in carpel specification (Nagasawa *et al.*, 2003; Sugiyama *et al.*, 2019), and maize *ZMM16* participating in carpel abortion and floral asymmetry processes (Bartlett *et al.*, 2015). A very recent report showed that the *Primula forbesii GLO2* gene only determines anther position by promoting growth of petals and stamen filaments, and that such a neofunctionalization is unlikely to have occurred at the level of gene expression, but is rather based on changes to the encoded protein (Huu *et al.*, 2020).

In the present study, we found that extending expression (also called heterotopic expression) of *DOLL1* into the carpel, and establishing regulation and genetic interactions with the carpel-expressed gene *PFCRC*, are essential for *DOLL1*, as a typical B-function gene, to have new roles in carpel and ovule development in *Physalis*. Our observations in *Physalis* provide new evidence relevant to understanding the neofunctionalization of B-function *MADS*-box genes in plants.

### *PFCRC* is a pleiotropic gene but underpins carpel formation

*CRC* genes are mainly expressed in the abaxial domain of the gynoecium starting at its inception, with obvious diversification (Yamaguchi *et al.*, 2004; Fourquin *et al.*, 2005; Orashakova *et al.*, 2009), supporting the suggestion that their roles differ within and among plants. The genes are involved in various processes affecting carpel morphogenesis and development, such as nectary development, floral meristem termination, and carpel fusion (Bowman and Smyth, 1999; Eshed *et al.*, 1999; Alvarez and Smyth, 2002; Morel *et al.*, 2018; Strable and Vollbrecht, 2019), albeit in carpel organ identity only in rice (Yamaguchi *et al.*, 2004; Sugiyama *et al.*, 2019). Arabidopsis *CRC* shows a dynamic expression pattern in the abaxial part of the valves, in four internal files of cells, and in the nectary glands; *crc* mutants had a gynoecium that was shorter and wider than the wild type gynoecium, and carpel fusion at the apex was impaired (Bowman and Smyth, 1999). The down-regulation of the *Eschscholzia californica CRC* ortholog (*EcCRC*) results in a reiteration of the fourth whorl, reminiscent of a matryoshka doll structure, and defects in carpel polarity and ovule initiation (Orashakova *et al.*, 2009). In most Solanaceae species tested, two copies of *CRC* paralogous genes exist, and they are involved in carpel development with potential redundant functions (Phukela *et al.*, 2020). The additional copy was detected in the *Physalis* genome but it apparently underwent pseudogenization; thus *PFCRC* is the unique *CRC* orthologous gene in *Physalis*. We found that *PFCRC* was predominantly expressed in carpel meristem and in the developing carpels, and that it exerted functions as an organ size regulator, possibly affecting fertilization–associated functionalities such as ovule development. These functions need further investigation; however, it was found that *PFCRC* was involved in floral organ identity specification, for example, in corolla and stamens, through genetic interactions with DOLL1. This is similar to the observations in Arabidopsis *crc-1 pi-1* double mutants, where the homeotic transformed third whorl organ resulting from *pi* mutation displayed reduced numbers of stigmatic papillae and ovules, but was still carpel-shaped (Alvarez and Smyth, 1999). However, we found that the *PFCRC* regulation by DOLL1 depended on the floral whorl context (Fig. 9A, B). The *PFCRC* activation released from *doll1* could repress *PFGLO2* that was ectopically expressed by the additional *PFCRC* mutation, thus restoring the homeotic transformation of floral whorl 2 and 3 in the *doll1* background, expressing negative epistasis. This also suggests that PFCRC may have a C-function, specifying carpels. However, in the fourth floral whorl, we revealed that *PFCRC* was activated by DOLL1, and that it had an additive effect, being primarily involved in the carpel developmental processes of carpel meristem determinacy, and functionality.

The *Physalis* gynoecium is composed of three distinct regions: the basal ovary consisting of two carpels, a style, and an apical stigma. When *PFCRC* was severely down-regulated or mutated, carpel meristem termination and carpel closure were disordered, thus producing multiple layers of opened and dorsiventral carpels, and each lamellar carpel pair appeared to be spirally arranged (Fig. 3). One or two exposed ovules occasionally resided at the base margin of the lamellar carpel structure of *pfcrc* mutants. No fruit was obtained after vigorous artificial pollination, hinting that fertilization-associated systems may likely be impaired in these mutants; this needs further investigation. The resultant gynoecium structure in *Physalis* shared certain similarities in a few aspects with those in Arabidopsis, *E. californica*, *Petunia*, and other plant species when the *CRC* function had been disturbed (Bowman and Smyth, 1999; Orashakova *et al.*, 2009; Yamaguchi *et al.*, 2017; Morel *et al.*, 2018), supporting conserved roles of *CRC* genes in carpel development. Nonetheless, it is striking to observe that loss-of-function of the single gene specifically changed the *Physalis* gynoecium morphology. Moreover, the transformed opened carpels in *pfcrc*-related mutants showed a vegetative growth property of trichome appearance at the transformed carpel tip. Thus, carpel organ identity was likely altered in *pfcrc* mutants, but how *PFCRC* determines carpel organ identity and development needs further investigation.

The dual roles of PFCRC between floral whorls 2/3 and whorl 4 might rely on two factors. Firstly, in the evolution of multiple CArG-boxes in its promoter, the *cis*-motifs bound by MADS-domain proteins had two distinct consequences: activator and repressor. Secondly, selective binding by putative DOLL1-associated protein complexes could regulate *PFCRC* expression in a context-dependent manner (Fig. 9A, B). Floral whorl 2/3 DOLL1-complex apparently repressed the *PFCRC* expression, and this regulation was also found in Arabidopsis and *Eschscholzia* (Alvarez and Smyth, 1999; Bowman and Smyth, 1999; Orashakova *et al.*, 2009). However, we found that floral whorl 4 DOLL1-complex normally activated *PFCRC* expression. The expression patterns of B-function genes *DOLL1* and *PFGLO2* were also differentially regulated by PFCRC in different contexts; for example, *DOLL1* itself was repressed in carpels (Fig. 9A, B). The mutual regulation mechanisms and the composition of the putative DOLL1-associated complexes in different contexts need to be clarified. Other MADS-box genes like *P. floridana AGAMOUS* (*PFAG*), a putative C-function gene (He *et al.*, 2007), could not be excluded from this complex. Nonetheless, *PFCRC* has apparently evolved into a pleiotropic gene in floral development and functionality in *Physalis*, as its accurate expression is primarily required for regulating carpel organ size, organ identity, meristem determinacy and functionality. These observations imply the association of the origin of carpels with that of *CRC*.

### Evolutionary implications for carpel origin

Both MADS-box and *CRC* genes are essential for carpel development. Elegant evolutionary analyses suggested that B-, C-, and E-function MADS-box genes originated in the most recent common ancestor (MRCA) of seed plants (Pfannebecker *et al.*, 2017a). *YABBY* genes are specific to seed plants (Yamada *et al.*, 2011; Bartholmes *et al.*, 2012; Finet *et al.*, 2016), while the *CRC* ortholog originated in the MRCA of angiosperms (Pfannebecker *et al.*, 2017b; Becker, 2020). Our phylogenetic analysis (Supplementary Fig. S33) and loss-of-function of *PFCRC* alone without affecting other floral organs, seemed to support these assumptions. However, the origin of the carpel was at least somewhat dependent on the establishment of CRC and MADS-box interactions. PFCRC could directly regulate *PFGLO2* via the predicted CRC-binding motifs, but it did not bind to the *DOLL1* promoter where the CRC binding motifs were also predicted. The CRC binding motifs were studied in a few species (Shamimuzzaman and Vodkin, 2013; Yamaguchi *et al.*, 2017), and the conservation of the consensus needs further investigations. However, MADS-domain transcription factors are known to bind to the CArG-box motif, the consensus sequence of CC(A/T)_6_GG and its variants (de Folter and Angenent, 2006; Wuest *et al.*, 2012; Pajoro *et al.*, 2014; Yan *et al.*, 2016; Aerts *et al.*, 2018). Moreover, multiple CArG-boxes occur on the promoters of *CRC* orthologs and are bound by functional MADS-box proteins in various plants (Lee *et al.*, 2005a; Wuest *et al.*, 2012; Ó’Maoiléidigh *et al.*, 2013; Morel *et al.*, 2018). In *P. floridana*, we found multiple putative CArG-box motifs in the *PFCRC* promoter, and we showed that DOLL1 and PFGLO2 were able to specifically bind the CArG-box motifs with the consensus sequence of CHW_2_AAW_2_DG, which could be an activator or a repressor. Our preliminary results showed that other *P. floridana* MADS-domain proteins such as A-function MPF3 (Zhao *et al.*, 2013), B-class PFDEF and PFTM6 (Zhang *et al.*, 2015), and the putative C-function PFAG (He *et al.*, 2007) also had the capability to bind to these motifs in a Y1H assay (Supplementary Fig. S34A), but their regulatory effects on the gene expression need evaluation. Furthermore, the characterized CArG-box motifs existed in all the promoters of *CRC* orthologs in the examined Solanaceae species (Supplementary Fig. S34B). Therefore, the genetic and regulatory interactions between MADS-domain proteins and the *CRC* promoters have been established. Unlike in most eudicots, both *GLO* and *CRC* orthologous genes are co-expressed in the inner two floral whorls in the basal angiosperms (Fourquin *et al.*, 2005; Kim *et al.*, 2005; Zhang *et al.*, 2020), hinting that these interactions and their roles in carpel specification might be ancestral.

In conclusion, we have shown that *DOLL1*, a classical floral-organ identity gene of floral whorls 2/3, gained a novel function in carpel development, and this neofunctionalization involves *PFCRC*, a predominant carpel regulator. Moreover, *PFCRC* is also involved in floral whorls 2/3 organ identity specification via genetic interaction with *DOLL1*, suggesting a new function of CRC orthologs outside of carpel development. Our findings further reveal the regulatory and genetic interactions between B-class MADS-box genes and *PFCRC* in a context-dependent manner. These findings in *Physalis* should facilitate comparative studies into the genetic regulatory networks regulating flower development to determine the relevance of the link between *DOLL1* and *PFCRC* for other plant species, and how such stepwise origin of key regulators and their interactions evolve to govern the morphological evolution of flowers and fruits.

## Supplementary data

The following supplementary data are available at *JXB* online.

Fig. S1. Floral development in WT *Physalis floridana* and *doll1* mutants.

Fig. S2. Fruit setting rate in *doll1* mutants under different conditions.

Fig. S3. Comparison of fruit morphologies between WT and *doll1* mutants.

Fig. S4. micro-CT analyses of pistils and fruits in *Physalis*.

Fig. S5. Stigma receptivity assays.

Fig. S6. Ovule-induced pollen tube guidance *in vitro*.

Fig. S7. Comparison of embryo sac development between WT and *doll1*.

Fig. S8. Differential expression of genes related to fertilization processes and embryo sac development between WT and *doll1* mutants.

Fig. S9. Vegetative variation after VIGS treatments in *P. floridana*.

Fig. S10. Floral variations of *PFCRC*-VIGS transgenic plants.

Fig. S11. Pseudogenization of the additional *PFCRC* homolog in *P. floridana*.

Fig. S12. Multiple sequence alignment (MSA) of CRC orthologs in various species.

Fig. S13. PFCRC sub-cellular localization and *PFCRC* expression.

Fig. S14. Generation and genotypic analysis of *PFCRC*-CRISPR/Cas9 transgenic *P. floridana* plants.

Fig. S15. Pollen morphology and maturation in various flowers.

Fig. S16. Phenotypic and genetic variation in F_2_ populations of *pfcrc-cas9-1* ♂ *×* WT *♀*.

Fig. S17. Phenotypic and genetic variation in F_2_ populations of *pfcrc-cas9-1* ♂ × *doll1 ♀*.

Fig. S18. Meristem morphology in *Physalis.*

Fig. S19. Epidermal cells of floral whorl 3 and carpels in the *pfcrc*^*-/-*^-related mutants.

Fig. S20. Analyses of *PFCRC* overexpressing (OE) transgenic plants.

Fig. S21. Genotypic and phenotypic analyses of *PFCRC-*OE-*doll1* double mutant.

Fig. S22. Floral expression of *DOLL1*, *PFGLO2* and *PFCRC* in *P. floridana*.

Fig. S23. Floral expression of *DOLL1* (*PFGLO1*), *PFGLO2* and *PFCRC* in different genetic backgrounds of *P. floridana*.

Fig. S24. Functional test of CArG-boxes in the *PFCRC* promoter by Y1H assays.

Fig. S25. The CArG-box motifs in the *PFCRC* promoter and constructs to detect DOLL1-*PFCRC* regulation.

Fig. S26. Site-directed mutagenesis of the CHW_2_AAW_2_DG CArG-boxes in yeast.

Fig. S27. Binding detection of HIS-DOLL1 and HIS-PFGLO2 fusion proteins to all CArG-box motifs of the *PFCRC* promoter in EMSA assays.

Fig. S28. Competition and site-directed mutagenesis of HIS-DOLL1 and HIS-PFGLO2 fusion proteins binding to the functional CArG-box motifs in the *PFCRC* promoter.

Fig. S29. Schematic diagram of *PFCRC* promoter pGreenII 0800-LUC related plasmid construction.

Fig. S30. PFGLOs regulate *PFCRC* expression in a dual-luciferase assay.

Fig. S31. PFCRC represses *PFGLO2* expression.

Fig. S32. Binding ability of PFCRC to the YBM motifs in the *PFGLO2* promoter.

Fig. S33. Phylogeny of *YABBY* genes in seed plants.

Fig. S34. Conservation of CArG-box motifs in Solanaceous species.

Table S1. Primers used in the present work.

Table S2. Statistics of ovule/seed number per flower/fruit of WT and *doll1*.

Table S3. DEGs from floral transcriptomic comparison between WT and *doll1*.

Table S4. Genes related to pollen-stigma perception and fertilization processes in Arabidopsis.

Table S5. Hybrid fruit setting rates of *PFCRC*-related transgenic plants.

Table S6. Seed plant *YABBY* genes used in the phylogenetic assay.

erab309_suppl_Supplementary_Figures

erab309_suppl_Supplementary_Tables

## Data Availability

All data supporting the findings of this study are available within the paper and within its supplementary materials published online.

## Abbreviations

CRC: *CRABS CLAW*
DOLL1: *double-layered-lantern 1*
DEG: differentially expressed genes
GLO: *GLOBOSA*
