# Supplementary material for: Physalis floridana CRABS CLAW mediates neofunctionalization of GLOBOSA genes in carpel development
Source: J Exp Bot. 2021 Jun 28;72(20):6882–903. doi: 10.1093/jxb/erab309 (PMC8547157; doi:10.1093/jxb/erab309)
Supplement: erab309_suppl_Supplementary_Figures [file erab309_suppl_supplementary_figures.pdf]

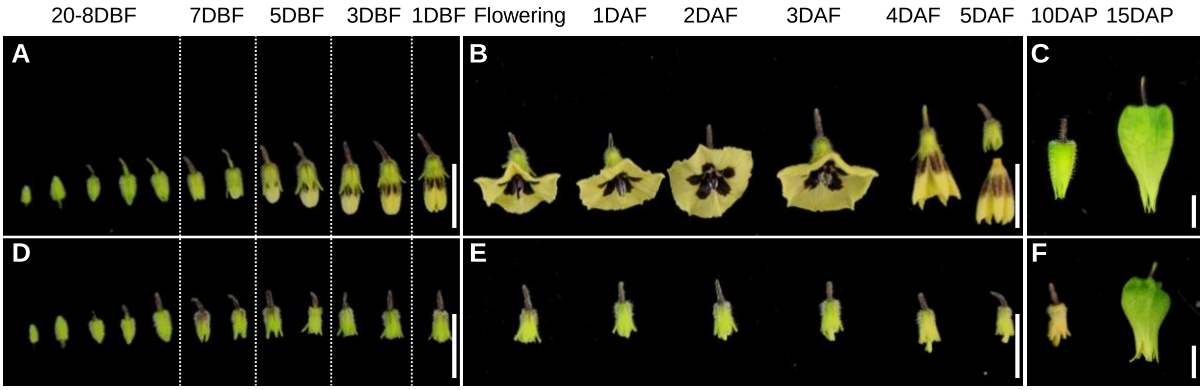

Fig. S1. Floral development in WT *Physalis floridana* and *doll1* mutants. (A–C) Morphology of floral buds (A), blooming flowers (B), and developing fruits (C) of WT *P. floridana*. (D–F) Floral and hybrid fruit morphologies of *doll1* mutants at the corresponding developmental stages as in the WT. DBF, days before flowering; DAF, days after flowering; DAP, days after pollination. Bars = 1.0 cm.

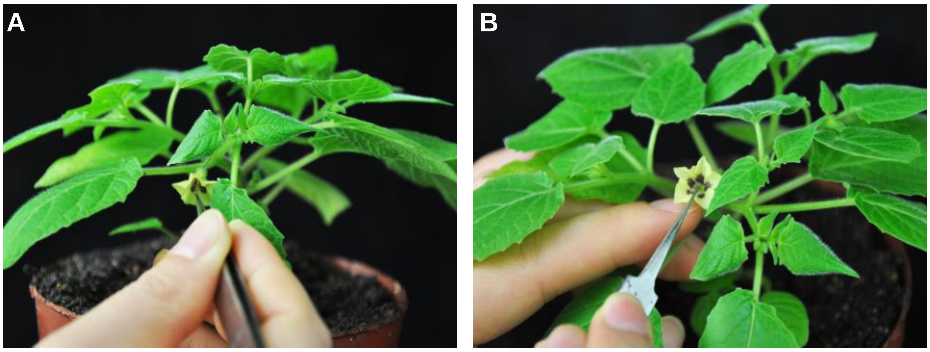

C Plants	Self-pollination		Normal artificial pollination		Erected artificial pollination	
	WT	*doll1*	WT	*doll1*	WT	*doll1*
No. of pollination	133	500	50	291	50	194
No. of bearing fruits	100	0	48	4	48	20
Fruit setting rate (%)	75.19	0**	96	1.37**	96	10.31**

Fig. S2. Fruit setting rate in *doll1* mutants under different conditions. (A) Normal artificial pollination. (B) Erected artificial pollination. (C) Statistics of fruit setting rate in WT and *doll1* mutants. **, Student's *t*-test, $P < 0.01$.

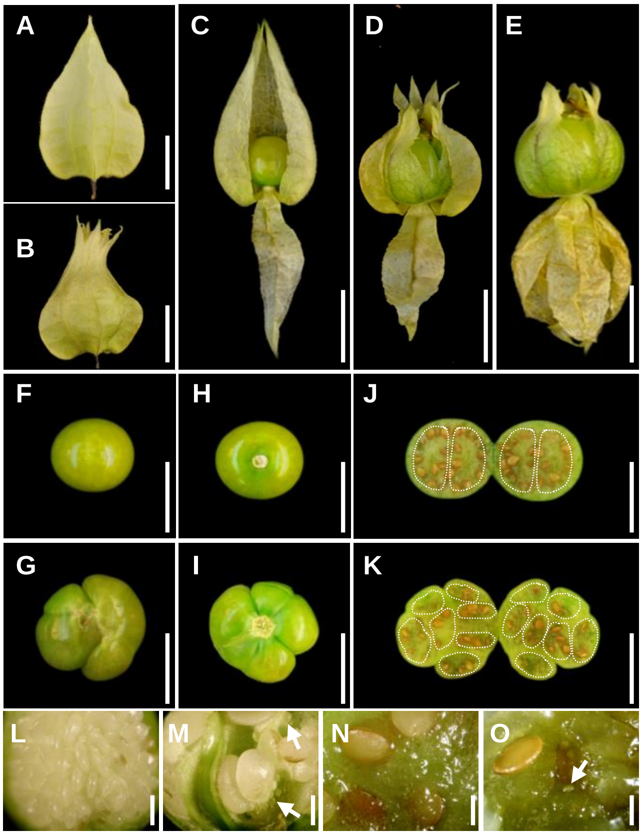

Fig. S3. Comparison of fruit morphologies between WT and *doll1* mutants. (A) A WT fruit. (B) A *doll1* fruit. (C) A WT fruit; part of the ICS was torn to show the berry inside. (D, E) A *doll1* fruit; the outside ICS was torn to show the second ICS and the berry inside. Longitudinal profile of ICS. (F, H) Front and antapical view of a WT berry. (G, I) Front and antapical view of a *doll1* berry. (J) The transverse section of a WT fruit. (K) The transverse section of a *doll1* berry. (L) WT seeds at two weeks after fertilization. (M) Seeds of *doll1* at two weeks after fertilization. (N) WT mature seeds. (O) *doll1* mature seeds. WT, wild type. Dotted curves define putative carpels. White arrows indicate the undeveloped ovules in *doll1*. Bars = 1.0 cm in (A) to (K); 1 mm in (L) to (O).

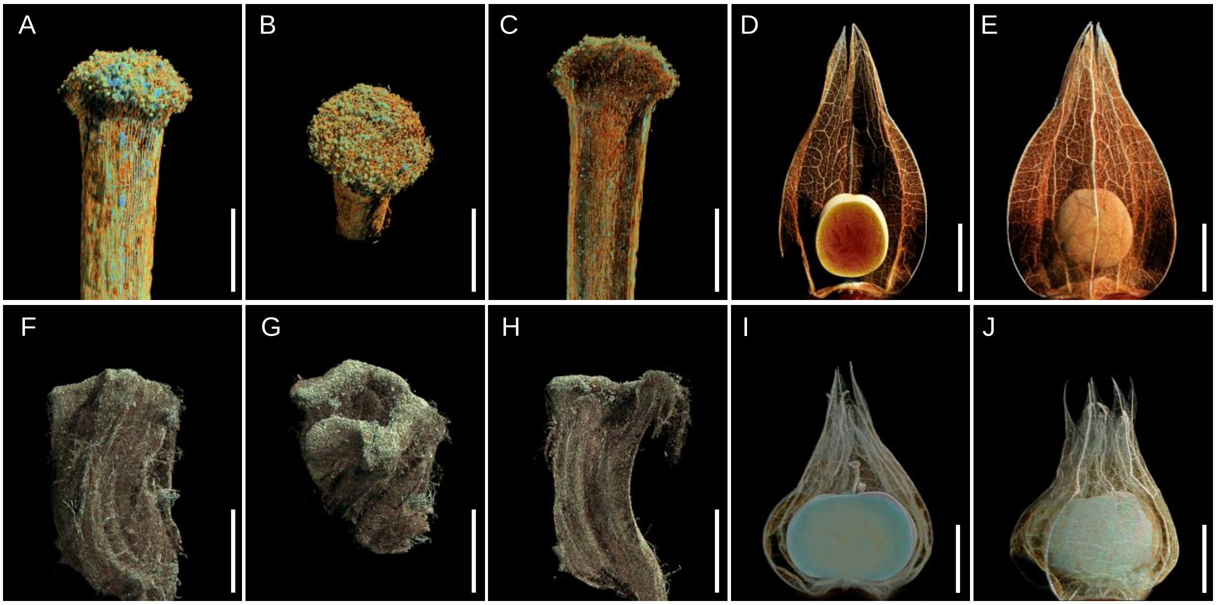

Fig. S4. micro-CT analyses of pistils and fruits in *Physalis.* (A–C) Pistil and stigma morphology and structure of WT. (D, E) Berry and Chinese lantern in the WT. (F–H) Native pistil and stigma morphology and structure of *doll1* (I, J) Berry and double Chinese lanterns in *doll1*. Bars = 500 µm in (A–C, F-H); 1.0 cm in (D), (E), (I), and (J). The complete video of the micro-CT assay is available upon request.

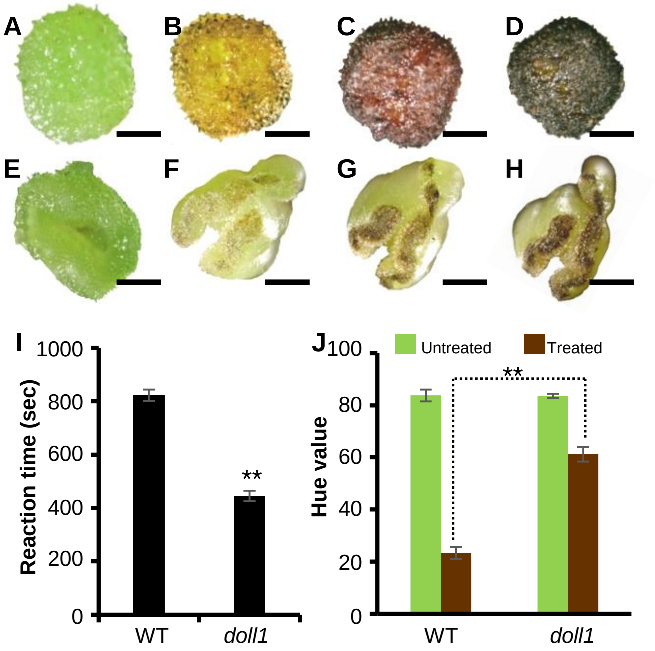

Fig. S5. Stigma receptivity assays. (A–D) Stigma receptivity in WT. (E–H) Stigma receptivity in *doll1*. Untreated control (A, E); treated after 1 sec (B, F); treated after 1 min (C, G); treated after 2 min (D, H). Bars = 1 mm. (I) Statistical analysis of maximum reaction time in WT and *doll1*. (J) Statistical analysis of browning degree of stigma after treating for 1 min in WT and *doll1* mutants measured by hue value with Photoshop software. ** in (I, J), Student's *t*-test, $P < 0.01$.

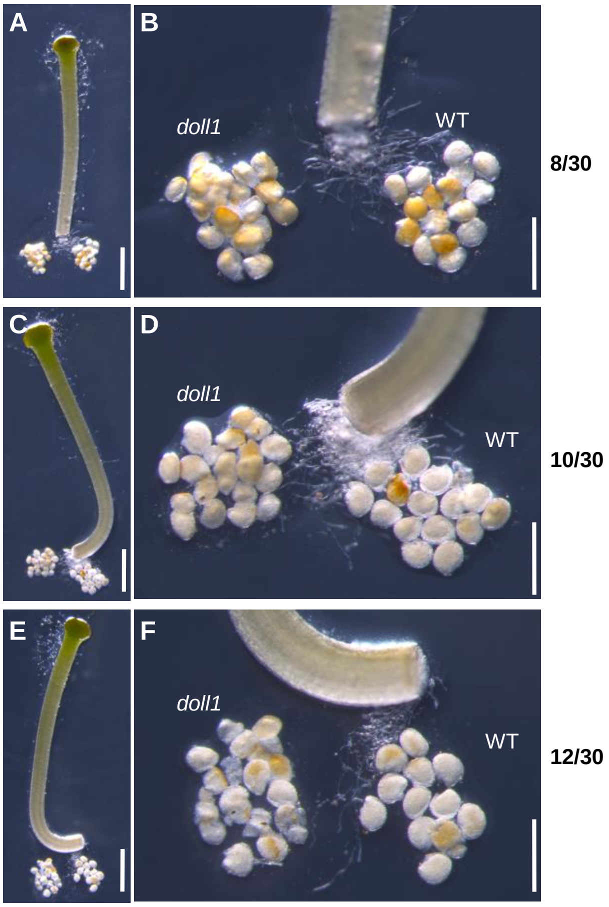

Fig. S6. Ovule-induced pollen tube guidance *in vitro.* The WT stigma was pollinated with WT pollen and then placed between the WT ovules and *doll1* native ovules. The style growth by chance had three possibilities. (A, B) The style grew straight. (C, D) Style bent toward the *doll1* ovules. (E, F) Style bent toward the WT ovules. However, the pollen tubes overall bent to the WT ovules. The adjacent ratios are the proportions of the three cases in 30 treatments. The images were taken 12 h after artificial pollination on the solid medium. Bars = 1 µm.

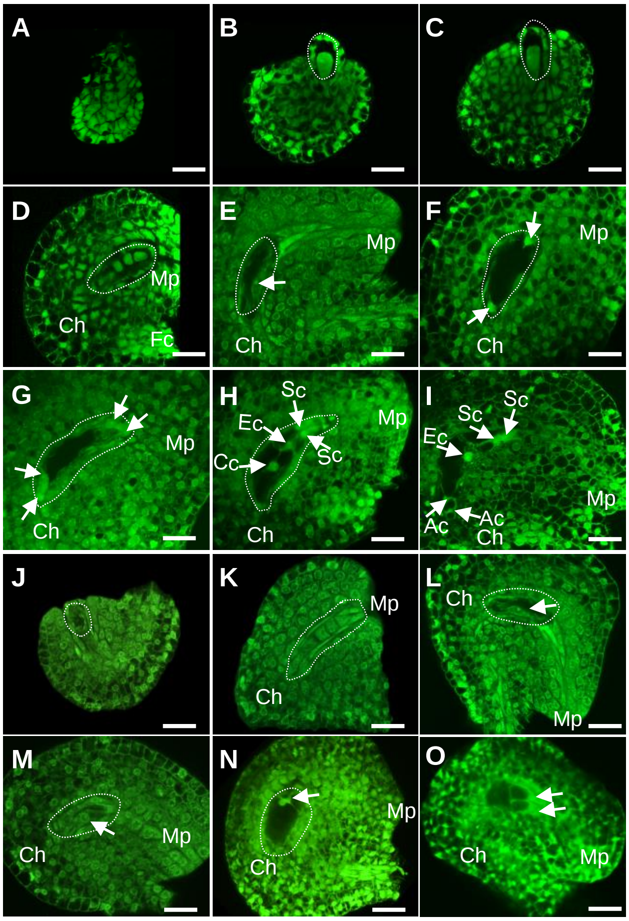

Fig. S7. Comparison of embryo sac development between WT and *doll1.* (A–I) Embryo sac developmental processes of the WT. (A) Ovule primordia stage. Nucellus cells near the micropyle were differentiated into sporogenous cells. (B, C) Megasporocyte stage. The sporogenous cells underwent an anticlinal division, and the inner cells developed into megaspore mother cells. (D) Tetrad megaspore stage. A megaspore mother cell underwent a meiotic division to form four linear megaspores, also called tetrad embryo sac stage. (E) Mononuclear embryo sac stage. Three of the four megaspore cells rapidly disappeared, and only a functional megaspore continued to develop into a mononuclear embryo sac. (F) Double nucleate embryo sac stage. The functional megaspore was divided into two separate cells, and these two nuclei were pushed to the two poles of the embryo sac by a large central vacuole. One nucleus was placed at the chalaza end, and the other was at the micropyle end, the so-called two-nucleate embryo sac stage. (G) Four-nucleate embryo sac stage. Cells at each end underwent mitosis to form a four-nucleate embryo sac. (H, I) Mature embryo sac stage, including three antipodal cells, two synergids, one egg, and a central cell. After the third mitosis, four cells were at each end, and an eight-nucleate embryo sac was formed. Then, one cell at each end was moved to the center and fused into the central cell. (J–O) Abnormal native embryo sac development in *doll1*. (J) Megasporocyte stage. (K) Tetrad megaspore stage. (L) Mononuclear embryo sac stage. (M) Double nucleate embryo sac stage. (N, O) Mature embryo sac stage. Ac, antipodal cell; Ch, chalazal end; Cc, central cell; Ec, egg cell; Mp, micropyle; Sc, synergid cell. Dotted lines, putative embryo sac profiles; white arrows, cells or nuclei. Bars = 20 μm.

A

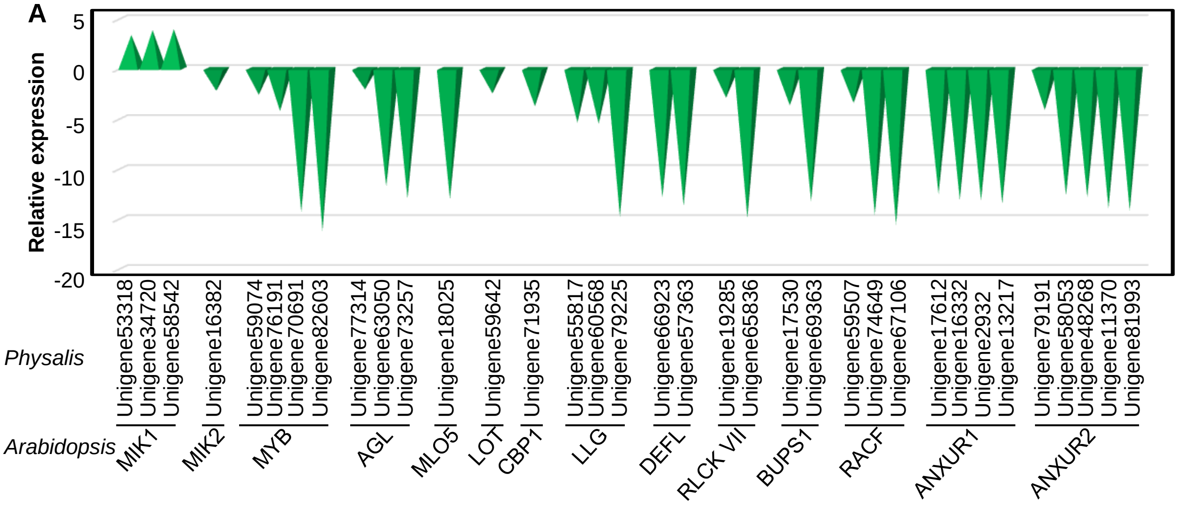

B

Order	Unigene in *P. floridana*	|log₂ Ratio| > 2	*P*- value	Gene locus in *Solanum lycopersicum*	expression level in carpel
1	Unigene61136	-18.53186046	2.93E-277	Solyc01g058520.2.1	L
2	Unigene24853	-18.4585367	9.99E-130	Solyc08g007150.1.1	H
3	Unigene69647	-15.6221737	2.17E-50	Solyc01g007950.2.1	H
4	Unigene1308	-11.89502388	3.54E-07	Solyc10g078890.1.1	L
5	Unigene37714	-7.564454437	3.60E-53	Solyc01g058530.2.1	L
6	Unigene15945	-6.573387562	7.47E-52	Solyc05g013350.2.1	H
7	Unigene16556	-5.867743579	4.30E-46	Solyc03g033690.1.1	M
8	Unigene75158	-4.481465072	4.18E-06	Solyc08g013930.2.1	H
9	Unigene16084	-3.692969178	3.23E-06	Solyc02g094180.2.1	L
10	Unigene73866	-3.555465654	1.18E-25	Solyc05g052280.2.1	L
11	Unigene3691	-3.513645478	3.26E-16	Solyc02g064970.2.1	H
12	Unigene39770	-3.108006677	1.57E-05	Solyc06g050440.2.1	M
13	Unigene32955	-2.81850006	1.68E-05	Solyc02g085930.2.1	M
14	Unigene64539	-2.692969178	5.95E-05	Solyc03g006700.2.1	M

C

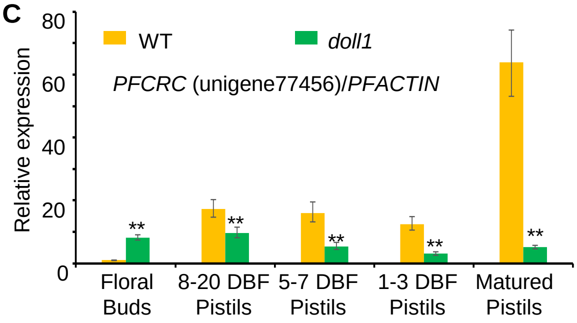

D

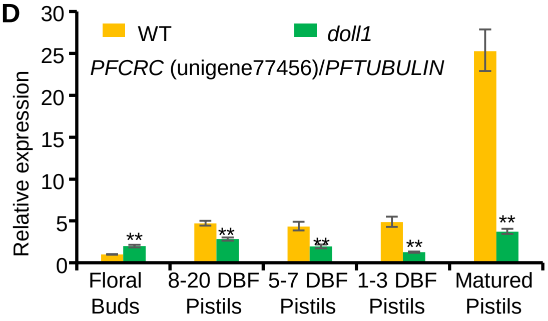

Fig. S8. Differential expression of genes related to fertilization processes and embryo sac development between WT and *doll1* mutants. (A) The differential expression of the homologous genes indicated. Relative expression was based on as *doll1*/WT in transcriptome comparison. DEGs were identified as $|\log_2 (doll1/WT)| \geq 2$ ($P \leq 0.01$). The functional information for *Arabidopsis* genes is available in Supplementary Table S4. (B) Down-regulation of the class III peroxidase family genes in *doll1* revealed by transcriptome data. Carpel expression was revealed as high (H), medium (M) and low (L) in WT by RT-PCR. (C) *PFCRC* expression between WT and *doll1* revealed by qRT-PCR. DBF, days before flowering. The expression level in WT floral buds was set as 1.0. *PFACTIN* and *PFTUBULIN* were used as the internal reference gene in (C) and (D), respectively. Three independent biological samples were used, and SD is presented. **， Student's *t*-test, $P < 0.001$. A full list of DEGs (unigenes) between WT and *doll1* is available in Supplementary Table S3.

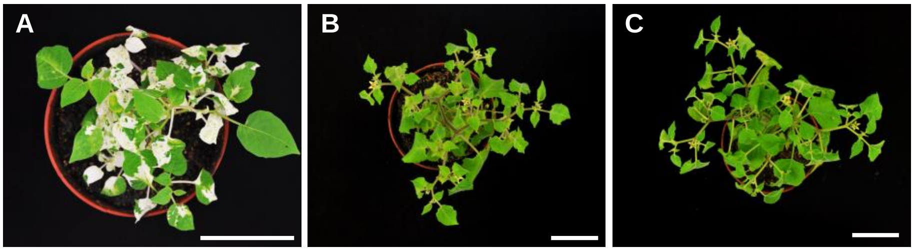

Fig. S9. Vegetative variation after VIGS treatments in *P. floridana.* (A) Phytoene desaturase gene (*PDS*)-VIGS transgenic plants. The occurrence of a photobleached leaf phenotype serves as a positive control of the VIGS system. (B) *TRV2* vector alone transgenic plants as the negative control. (C) *PFCRC*-VIGS transgenic plants. Bars = 10 cm. These indicate the specificity of floral phenotypic variation in *PFCRC*-VIGS transgenic plants.

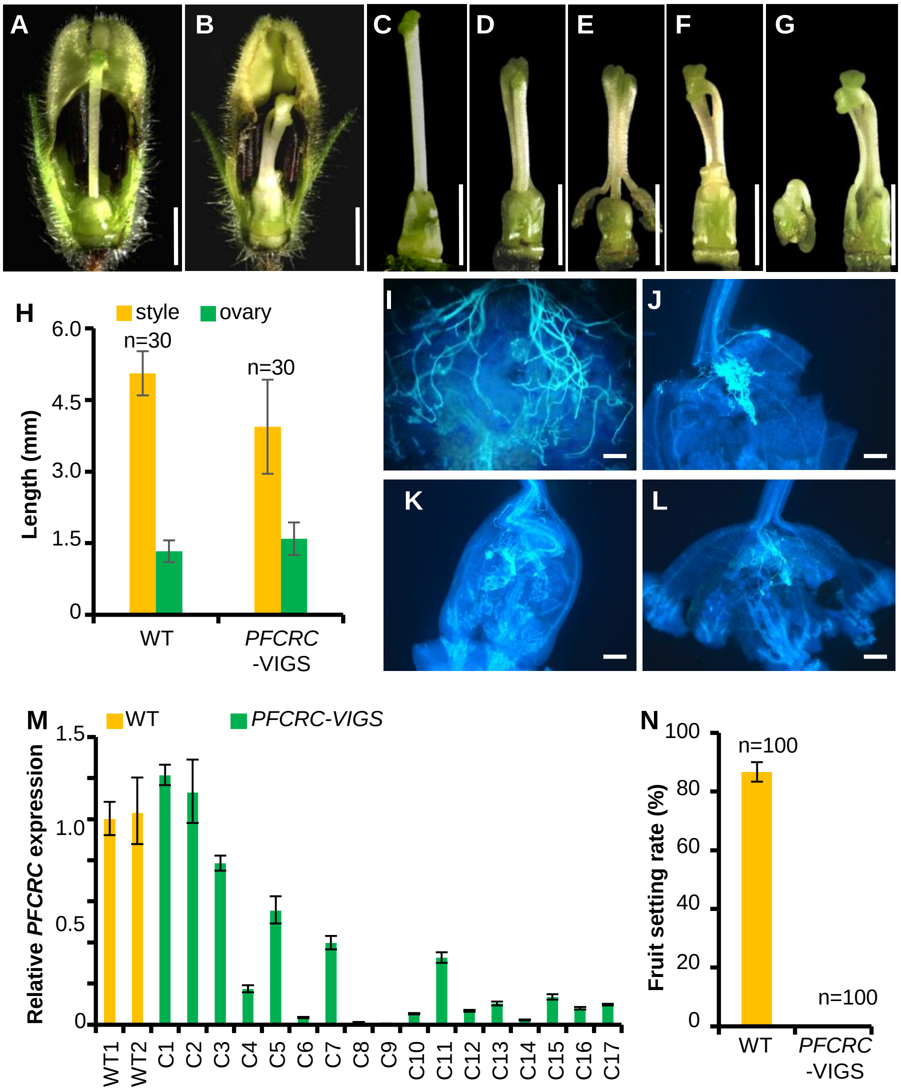

Fig. S10. Floral variations of *PFCRC*-VIGS transgenic plants. (A) Floral bud of WT. (B) Floral bud of *PFCRC*-VIGS mutant. Partial sepal and petal were peeled off. (C) Pistil morphology of WT. (D–G) Variants of pistil morphology in *PFCRC*-VIGS. (H) Style and ovary length between WT and *PFCRC*-VIGS carpels. Thirty samples in each background were measured. (I) Pollen tube growth in the WT ovary. (J–L) Pollen tube growth in the *PFCRC*-VIGS ovaries. (M) Genotypic analyses of *PFCRC*-VIGS mutants. The *PFCRC* expression in mature pistils was measured (n = 3). The *PFCRC* expression in one WT pistil was set as 1.0. The *PFACTIN* was used as the internal reference gene. (N) Fruit setting rate of *PFCRC*-VIGS mutants. Bars = 2 mm in (A–G); 200 µm in (I–L).

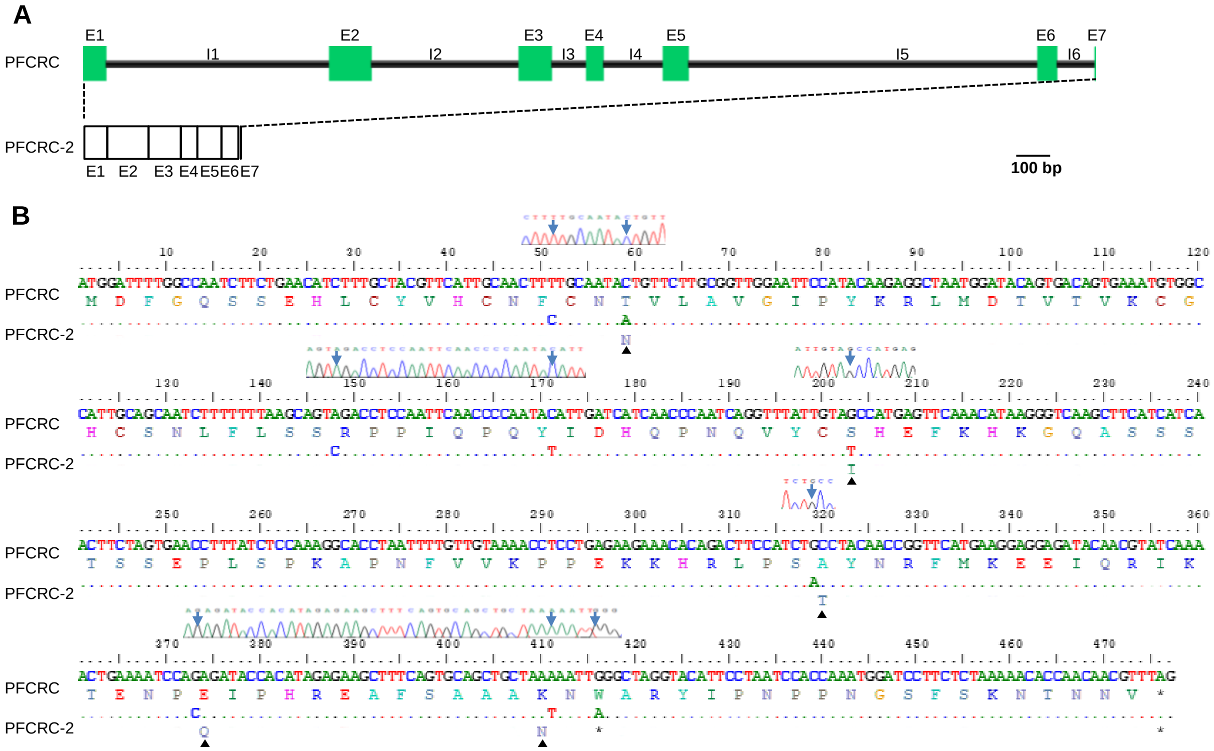

Fig. S11. Pseudogenization of the additional *PFCRC* homolog in *P. floridana.* (A) Gene structure of *PFCRC* and its close homolog *PFCRC-2*. Compared to *PFCRC* genome sequence, *PFCRC*-2 was intronless and its putative genomic sequence was 477 bp. E, exon; I, intron. (B) Putative *PFCRC-2* coding sequence (CDS) compared to *PFCRC*. Nine SNPs between *PFCRC-2* and *PFCRC*, resulting in substitution of five amino acids and a premature stop codon in *PFCRC-2*. No transcript of *PFCRC-2* was obtained either in transcripotimic data or by RT-PCR. 477 bp RT-PCR fragment was amplified using *P. floridana* floral organ cDNA as the template with a pair of universal primer, and only *PFCRC* transcript was found by PCR product sequencing. Sequencing maps on the SNPs were shown, and only *PFCRC* loci was found. Blue arrows, putative polymorphic sites. Color dots, identical nucleotide acids; black rectangles, diverged amino acids; black stars, stop codons.

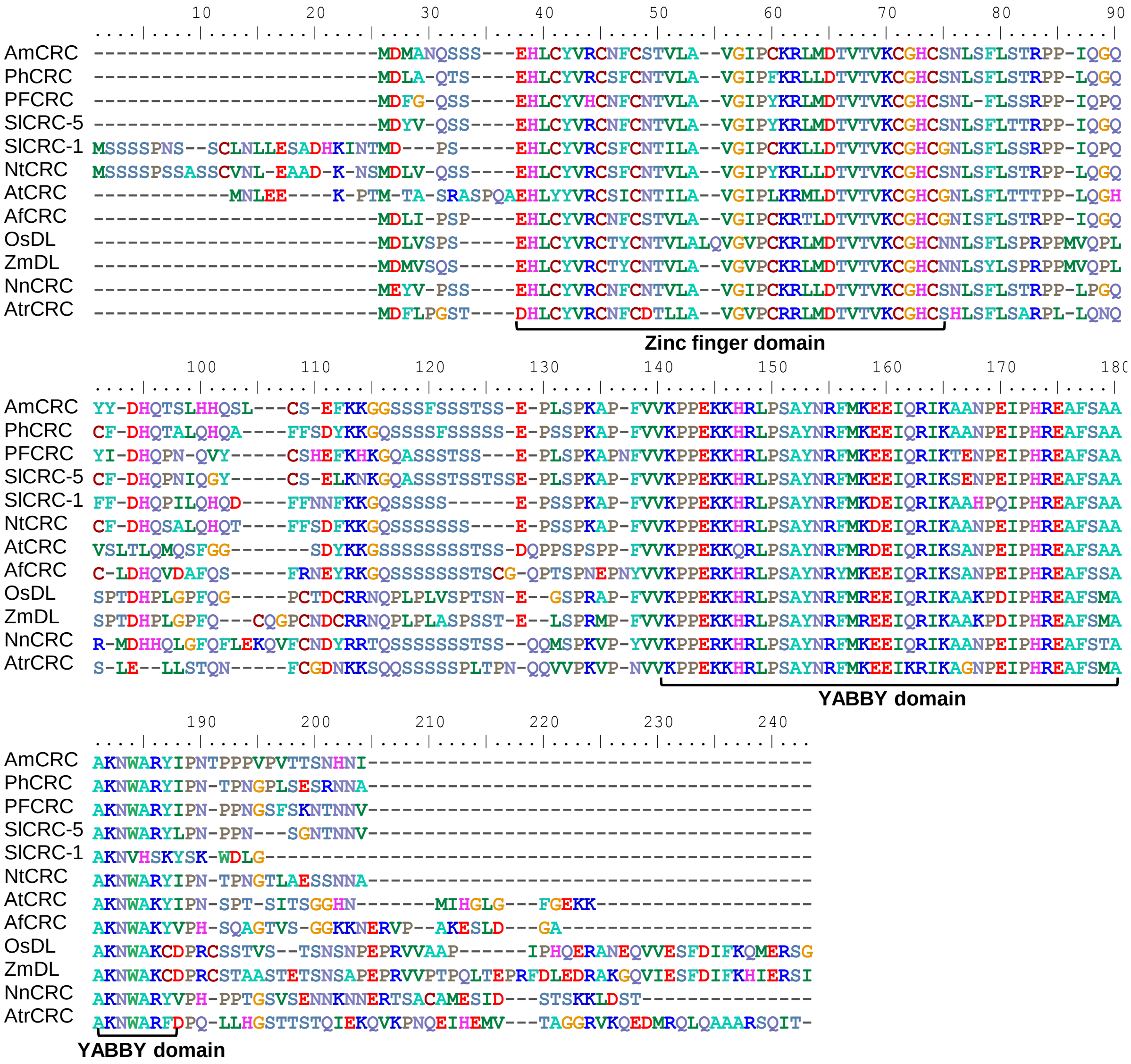

Fig. S12. Multiple sequence alignment (MSA) of CRC orthologs in various species. Two domains are labeled by black cross braces. Sl, *Solanum lycopersicon*; PF, *Physalis floridana*; Am, *Antirrhinum majus*; Nt, *Nicotiana tabacum*; Ph, *Petunia x hybrida*; At, *Arabidopsis thaliana*; Nn, *Nelumbo nucifera*; Af, *Aquilegia formosa*; Os, *Oryza sativa*; Zm, *Zea mays*; Atr, *Amborella trichopoda*.

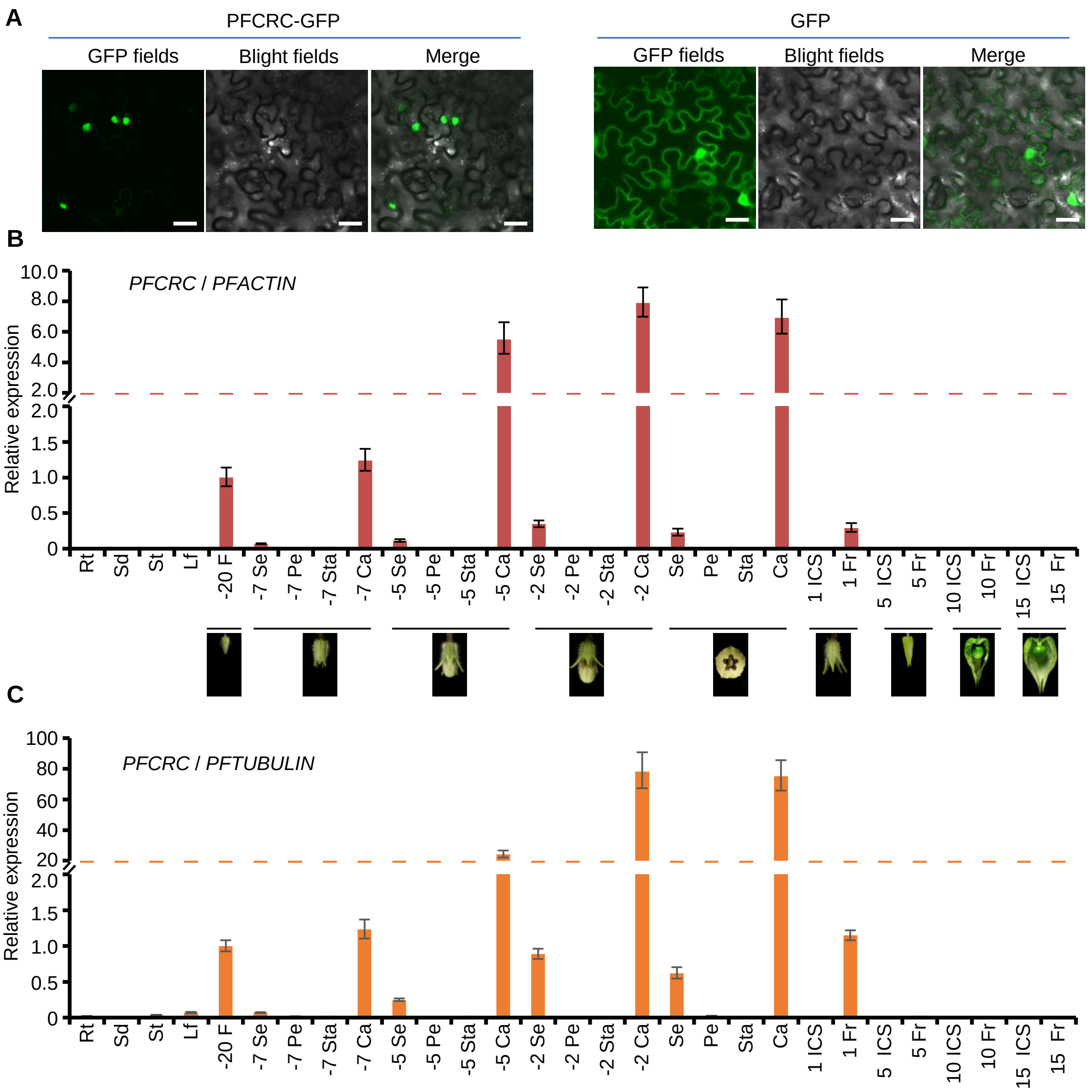

Fig. S13. PFCRC sub-cellular localization and *PFCRC* expression. (A) Subcellular localization of PFCRC in tobacco (*Nicotiana benthamiana*) leaf epidermal cells. GFP signals were empty vector controls. Bars = 200 µm. (B) and (C)Tissue-specific expression pattern of *PFCRC* by qRT-PCR assays. *PFACTIN* and *PFTUBULIN* were served as an internal controls in (B) and (C), respectively. Error bars indicate SD. Each reaction was performed in three biological repeats. Rt, root; Sd, seedlings; St, stem; Lf, leaf; -20 F, floral buds of 20 days before flowering; -7 (-5, -2) Se, sepal of 7 (5, 2) days before flowering; -7 (-5, -2) Pe, petal of 7 (5, 2) days before flowering; -7 (-5, -2) Sta, stamen of 7 (5, 2) days before flowering; -7 (-5, -2) Ca, carpel of 7 (5, 2) days before flowering. 1-, 5-, 10-, and 15-ICS, Chinese lantern of 1, 5, 10, and 15 days after fertilization; 1-, 5-, 10-, and 15-Fr, berry of 1, 5, 10, and 15 days after fertilization.

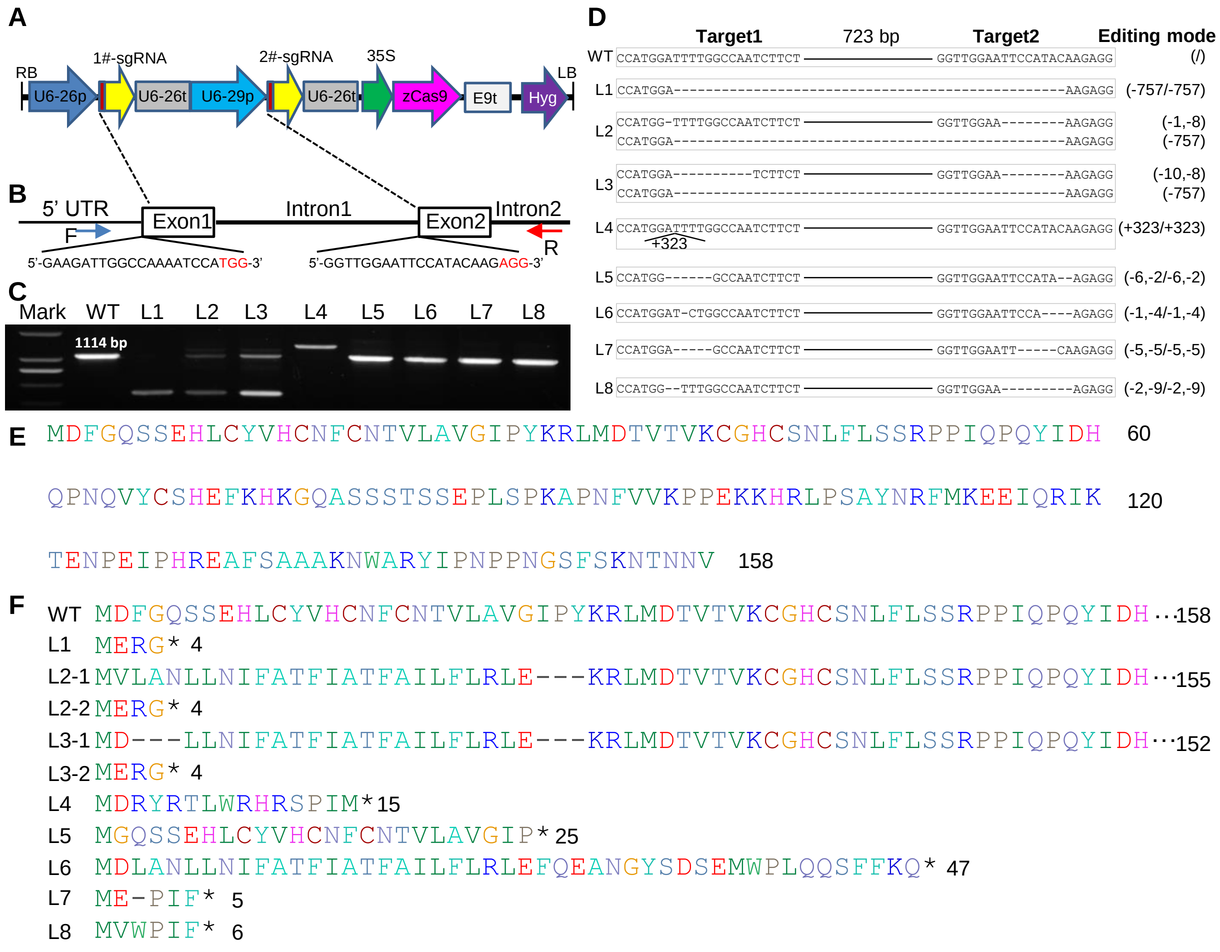

Fig. S14. Generation and genotypic analysis of *PFCRC*-CRISPR/Cas9 transgenic *P. floridana* plants. (A) Main structure of the CRISPR/Cas9 binary vector harboring Cas9 driven by the 35S promoter, and two sgRNA genes driven by *Arabidopsis* U6 gene promoters U6-26p and U6-29p, respectively. RB/LB, T-DNA right/left border; E9t, E9 gene terminator; zCas9, *Zea mays* codon-optimized Cas9; U6-26p and U6-29p, two *Arabidopsis* U6 gene promoter; U6-26t, U6-26 terminator; Hyg, hygromycin-resistance gene. (B) Two guide RNA targets used in gene editing vector construction. F and R stand for forward and reverse primers, respectively, that were used for genotypic analyses of *PFCRC* editing transgenic plants. (C) Gene editing patterns in the indicated transgenic plants detected by genomic PCR. Mark, Marker of DL2000 bp DNA ladder. (D) Gene editing patterns in the obtained transgenic plants detected by sequencing. A 723-bp fragment was flanked between these two targeting sites. +, nucleotide acids insertion; -, nucleotide acids deletion. (E) WT PFCRC protein sequence. (F) PFCRC protein alterations in gene editing of transgenic plants compared to WT. L2-1, L2-2 and L3-1, L3-2 are different editing forms of heterozygous lines L2 and L3, respectively. *, premature stop codons; -, gaps. Amino acid number is given at the right side.

These transgenic plants displayed a variety of *PFCRC* gene editing modes, including five homozygous deletion lines (L1, L5, L6, L7, and L8), one homozygous insertion line (L4), and two heterozygous mutant lines (L2 and L3). These gene editing events led to various abnormalities in the putative encoding protein, including premature stop codons, amino acid deletions, and frame-shift mutations, thus abolishing the PFCRC function. All homozygous mutants displayed similar floral phenotypic alterations. In L1, named *pfcrc-cas9-1*, a 757-bp genomic fragment was deleted, resulting in a 79 bp deletion of CDS in exon1 and exon2 causing a frame-shift mutation.

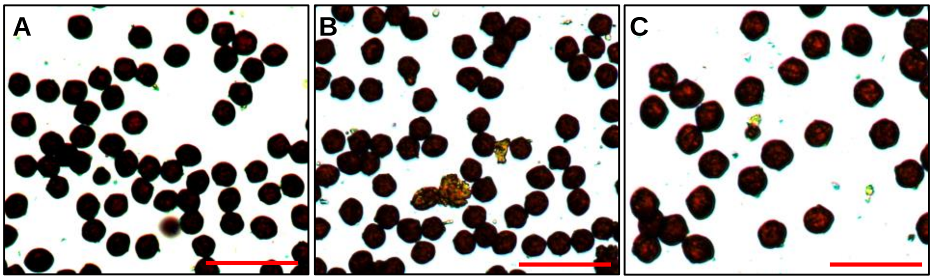

Fig. S15. Pollen morphology and maturation in various flowers. (A) Pollen maturation in WT flowers. (B) Pollen maturation in *pfcrc-cas9-1* transgenic flowers. (C) Pollen maturation in *PFCRC*-OE transgenic flowers. Pollen maturation was evaluated by using I_2-KI staining. Bars = 100 µm.

A

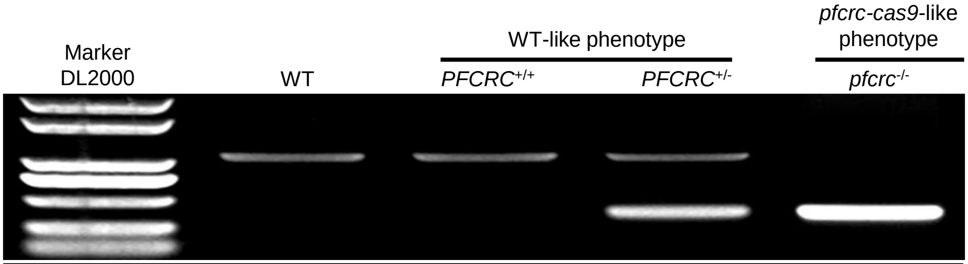

B

**Phenotype**	**F_2**	**WT-like**		***pfcrc-cas9-like***	**Separation ratio**	**χ2**	***p*-value**
**Genotype**		***PFCRC*$^{+/+}$**	***PFCRC*$^{+/-}$**	***pfcrc*$^{-/-}$**	1:2:1		
Observed genotype plants		50	108	51			
Observed phenotype plants	209	158		51	≈ 3 : 1	0.04	0.84
Expected phenotype plants		156.75		52.25			

Fig. S16. Phenotypic and genetic variation in F_2 populations of *pfcrc-cas9-1* ♂ × WT ♀. (A) Genetic identification. (B) Phenotypic and genetic variation. The genotype and phenotype, and the individual numbers of each type are given.

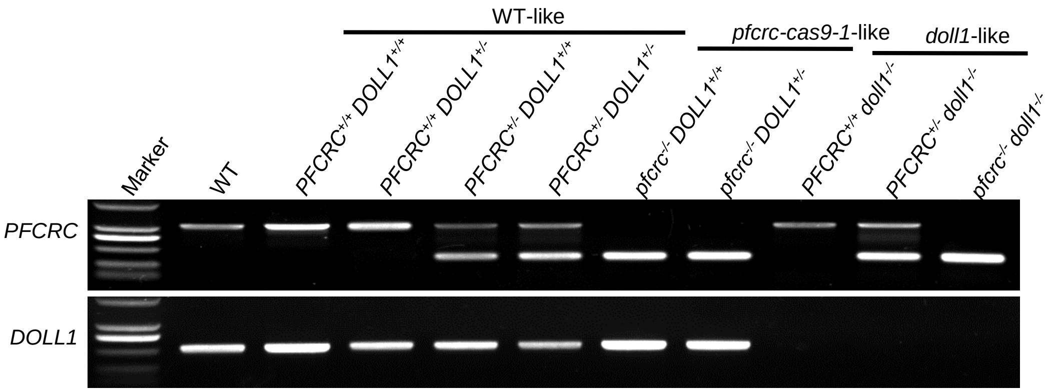

Fig. S17. Phenotypic and genetic variation in F_2 populations of *pfcrc-cas9-1* ♂ × *doll1* ♀. The same PCR type could have different phenotypes. Molecular markers of the heterozygous *DOLL1*$^{+/-}$ have not yet been developed, thus are currently indistinguishable from the WT genotype. Each individual could be identified by the combination of phenotype and genotype, and the numbers of each genotype or phenotype in the F_2 population are given.

The F_1 plants, named *PFCRC*$^{+/-}$*DOLL1*$^{+/-}$, grew as in the normal WT, again indicating that both mutations were recessive.

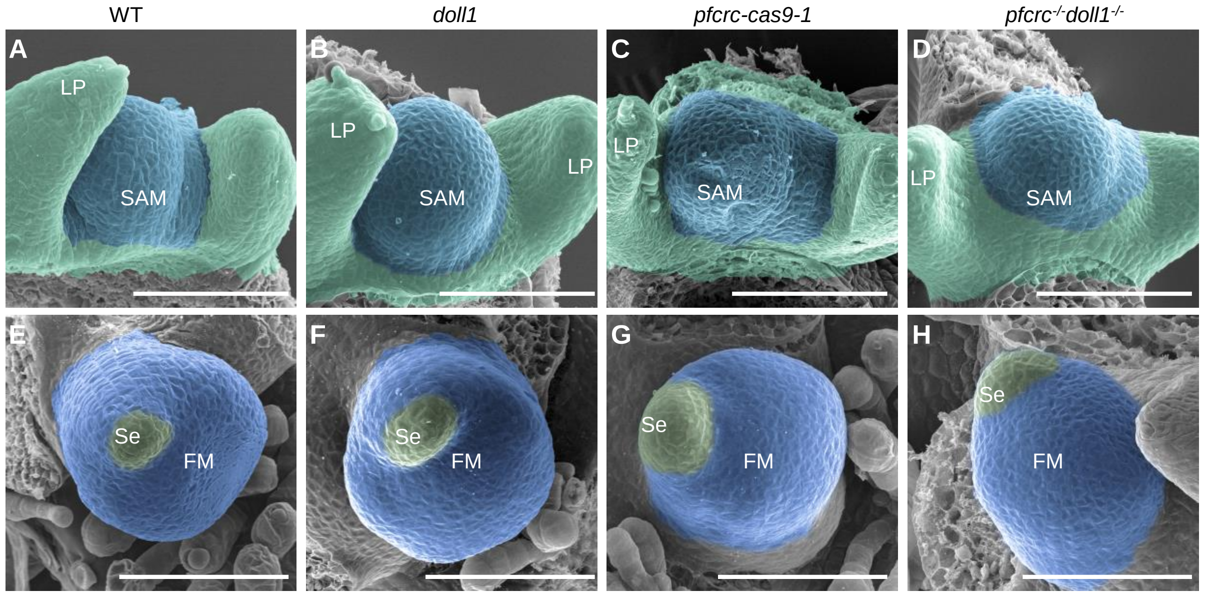

Fig. S18. Meristem morphology in *Physalis.* (A-D) Stem apex meristem (SAM) stage of indicated genetic backgrounds. LP, leaf primordia. (E-H) Floral meristem (FM) stage of indicated genetic backgrounds. The first sepal primordium (Se) was initiated. Bars = 100 µm.

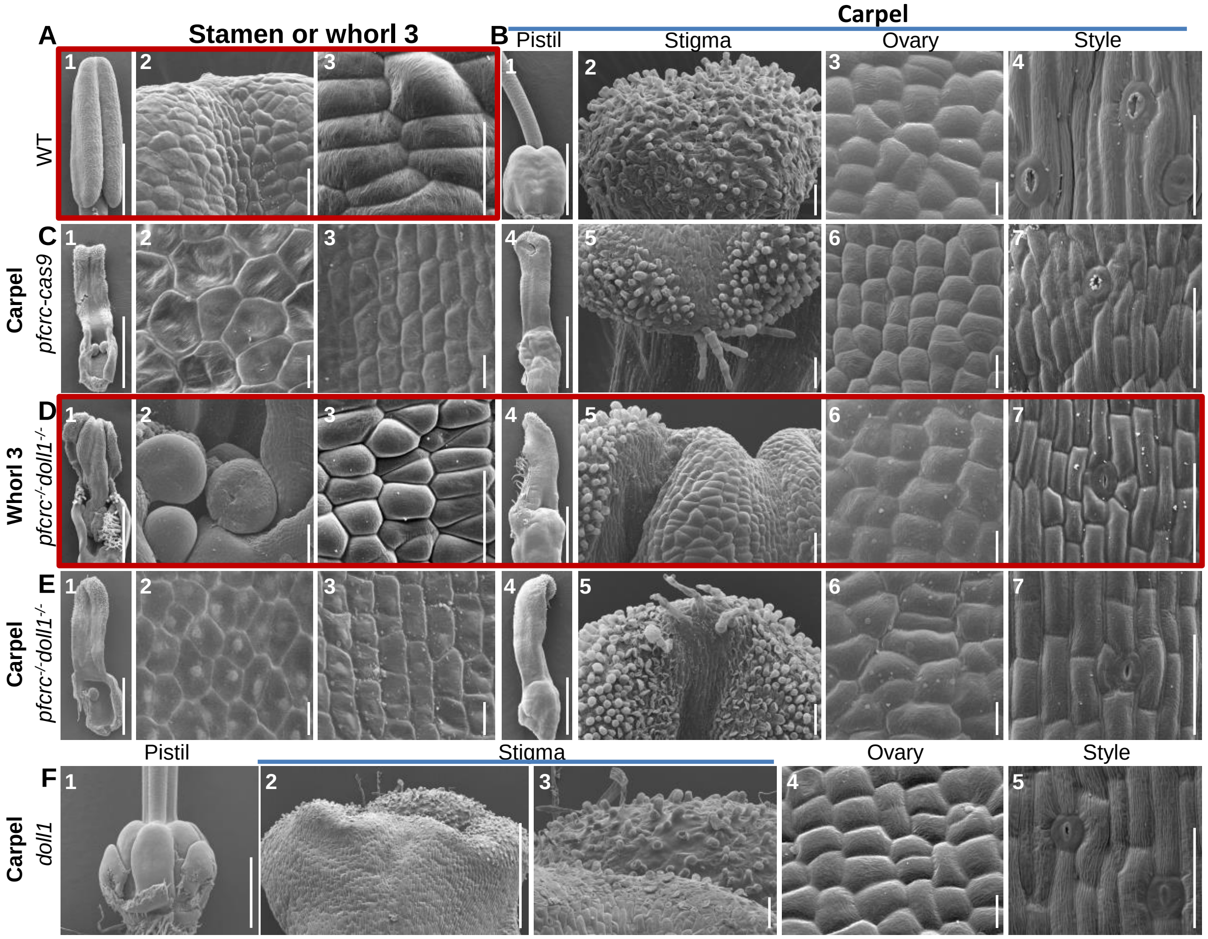

Fig. S19. Epidermal cells of floral whorl 3 and carpels in the *pfcrc*$^{-/-}$-related mutants. (A) Stamen cell morphology of the wild type (WT). (A1–A3) Adaxial side cell morphology of stamen organ. (B) Carpel cell morphology of WT. (B1) Ovary with partial style. (B2) Stigma. (B3) Epidermal cell morphology of ovary. (B4) Epidermal cell morphology of style. More than 100 stomatal apparatus cells were scattered in one whole style. (C) Cell morphology of the scale-like carpel organ of *pfcrc-cas9* mutants. This scale-like organ could be divided into three parts, the inflated ovary, applanate style and stigma. (C1) Adaxial surface of the scale organs. In each organ, one or two abortive ovules were located at the basal part. (C2, C3) Adaxial cell morphology of ovary and style. (C4) Abaxial surface of the scale organs. (C5) Epidermal cell morphology of stigma organs. (C6, C7) Abaxial cell morphology of ovary and style organ. Less than 10 stomatal apparatus cells were detected in one style organ (C7). (D) Cell morphology of the third whorl floral organ of the *pfcrc*$^{-/-}$*doll1*$^{-/-}$ double mutant. (D1) A chimera was formed in the *pfcrc*$^{-/-}$*doll1*$^{-/-}$ double mutant, including carpel- and stamen-like structures. (D2) Few ovule-like organs were located at the base of stamen-like organ. (D3) Adaxial cell morphology of a stamen-like organ. (D4) Abaxial cell morphology of the chimera organs. (D5) Cell morphology of the top area of the chimera. The outer section resembled the stigma organ of the WT, whereas the middle part was similar to WT stamen structure. (D6) Abaxial side cell morphology of the ovary-like section. (D7) Abaxial side cell morphology of the style-like section. Morphology of epidermal cells was similar to the WT, and no more than 10 stomatal apparatus cells were present, indicating carpel-like identity of the outline of this chimera structure. (E) Cell morphology of a scale-like carpel organ of the *pfcrc*$^{-/-}$*doll1*$^{-/-}$ double mutant. (E1) Adaxial morphology of a carpel organ, also including an inflated ovary, applanate style and stigma. One or two ovules were located in the bottom of each scale-like carpel organ. (E2, E3) Adaxial cell morphology of ovary- and style-sections. (E4) Abaxial morphology of the carpel organ. (E5) The surface of stigma-like organs. (E6) Abaxial epidermal cell morphology of ovary-like organ. (E7) Abaxial epidermal cell morphology of style-like organ. Around 30 stomatal apparatus cells are seen. (F) Fused carpel morphology of the *doll1* mutant. (F1) Fused ovary and partial style organ. (F2, F3) Stigma morphology. (F4) Ovary epidermal cell morphology. (F5) Style epidermal cell morphology. Bars = 1 mm in (A1), (B1), (C1), (C4), (D1), (D4), (E1), (E4) and (F1); 50 μm in (A2) (A3) (B2), (B4), (C5), (C7), (D3), (D5), (D7), (E5), (E7), (F2), (F3) and (F5); 10 μm in (B3), (C2), (C3), (C6), (D6), (E2), (E3), (E6), and (F4); 100 μm in (D2).

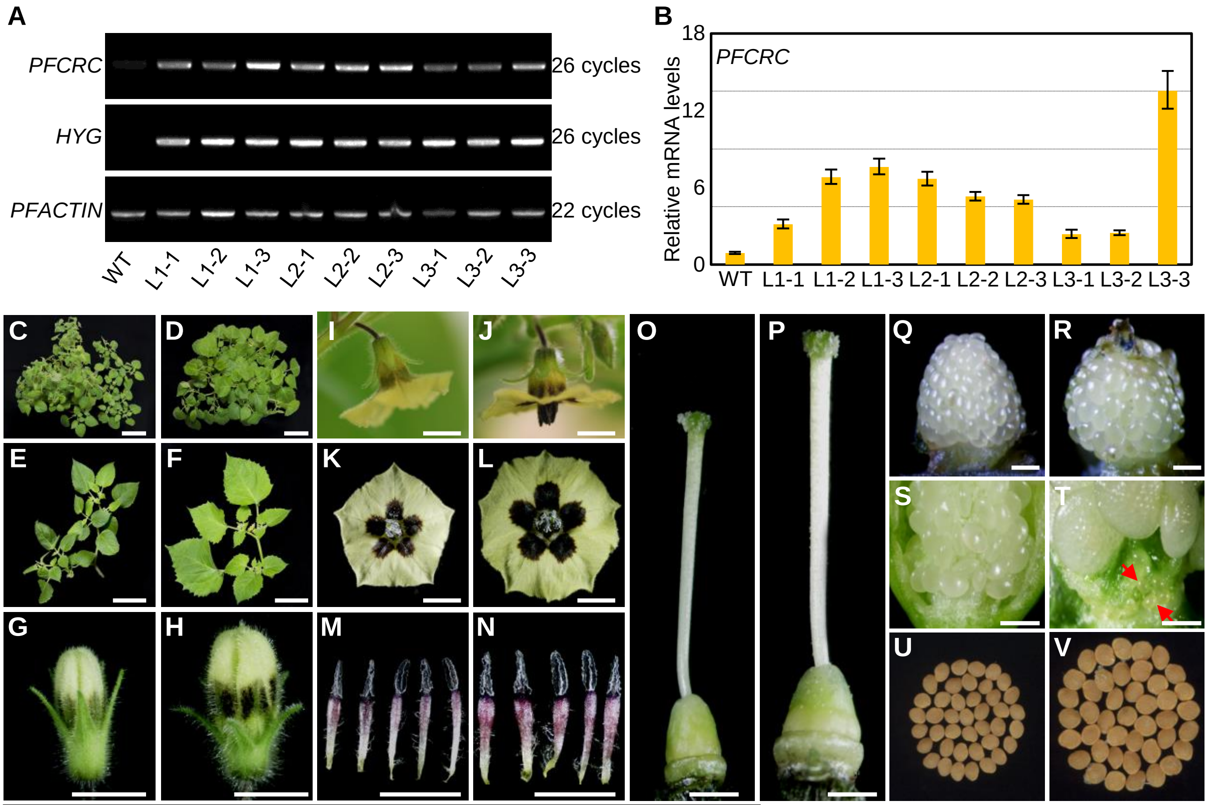

W

Genotype	n	ovule number in single carpel (ONSF)	seed number in single fruit (SNSF)	seed setting rate	single fruit weight (SFW, g)	hundred-grain weight (HGW, mg)
WT	30	152.28 ± 20.31	147.56 ± 51.28	96.9%	0.81 ± 0.28	44.18 ± 3.61
PFCRC-OE	30	131.63 ± 12.79	41.07 ± 12.13	31.2%	0.63 ± 0.17	91.31 ± 4.34

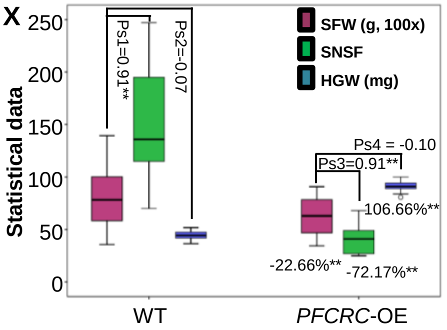

Fig. S20. Analyses of *PFCRC* overexpressing (OE) transgenic plants. (A) Genotypic analysis of *PFCRC*-OE transgenic plants using semi-quantitative RT-PCR. *PFACTIN* was used as an internal reference. The expression of the resistance to hygromycin gene (*HYG*) was used as a transgenic selecting marker. The amplification cycles are given. (B) The *PFCRC* expression in transgenic plants revealed by qRT-PCR. The *PFACTIN* was used as the internal reference gene. (C, D) Plant morphology of WT and *PFCRC*-OE plant. (E, F) Leaf size between WT and *PFCRC*-OE plants. (G, H) Floral buds between WT and *PFCRC*-OE plants. (I-L) Flowers between WT and *PFCRC*-OE plants. (M, N) Mature stamens between WT and *PFCRC*-OE plants. (O, P) Mature pistils between WT and *PFCRC*-OE plants. (Q, R) Ovule and placenta before fertilization between WT and *PFCRC*-OE plants. (S, T) Young seeds at around 10 days after fertilization between WT and *PFCRC*-OE plants. (U, V) Mature seeds between WT and *PFCRC*-OE plants. Bars = 10 cm in (C) and (D); 5 cm in (E) and (F); 5 mm in (G-N); 1 mm in (O-T). (W) Statistics of ovule number per carpel, seed number per berry and sell-fruit setting rate. ONSF, ovule number in a single carpel; SNSF, seed number in a single fruit; SFW, single fruit weight (g); HGW, hundred-grain weight (mg). (X) The correlations between seed number, seed size and berry size. Ps, Pearson correlation coefficient; Ps1 and Ps3, Pearson correlation coefficient of SFW and SNSF; Ps2 and Ps4, Pearson correlation coefficient of SFW and HGW. **, Student's *t*-test, *P* < 0.01.

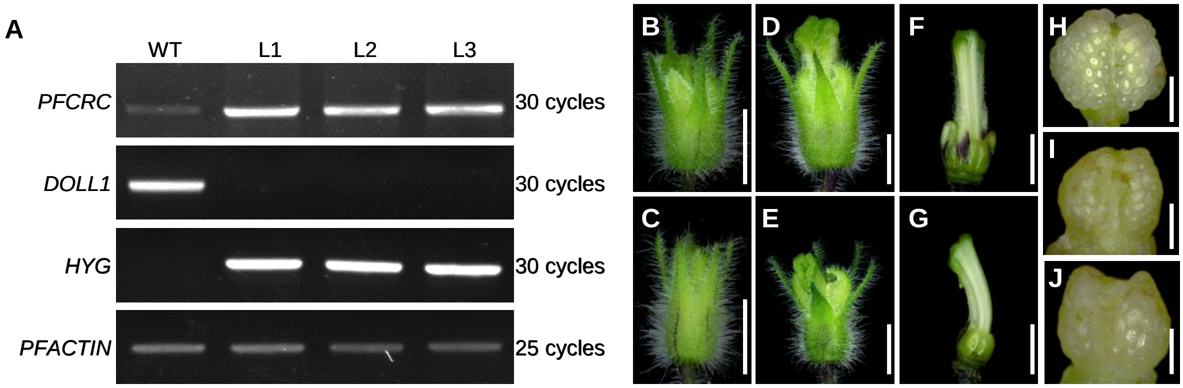

K

Genotype	n	Ovule number per carpel			Seed number		Seed setting rate (%)		Fruit setting rate by erected artificial pollination (%)
		Total	Native	Transformed	Native	Transformed	Native	Transformed	
PFCRC-OE-*doll1*	30 (Carpel) 7 (Fruit)	146.33 ± 26.31	87.80 ± 8.35**	56.40 ± 17.64	42.14 ± 5.37**	22.33 ± 6.87	47.80 ± 6.33**	39.34 ± 5.21	35** (7/20)

Fig. S21. Genotypic and phenotypic analyses of *PFCRC*-OE-*doll1* double mutant. (A) Genotypic analyses of *PFCRC*-OE-*doll1* double mutant. Gene expression in floral buds was detected by using a semi-quantitative RT-PCR assay. *PFACTIN* was used as an internal reference; the *HYG*, hygromycin gene was detected as a transgenic marker. The amplification cycles for each gene are given. (B) Floral bud morphology of *PFCRC*-OE-*doll1* double mutant five days before maturation. (C) Floral bud morphology of *doll1* mutant five days before maturation. (D) Mature floral morphology of *PFCRC*-OE-*doll1* double mutant. (E) Mature floral morphology of *doll1* mutant. The maturation of flowers in the mutants is relative to the blooming of WT. (F) Fused carpel morphology of *PFCRC*-OE-*doll1* double mutant. (G) Fused carpel morphology of *doll1* mutant. (H) Ovules of WT. (I) Native ovules in *doll1*. (J) Native ovules in *PFCRC*-OE-*doll1*. (K) Statistics of ovule/seed number per flower/fruit of *PFCRC*-OE-*doll1*. **, Student's *t*-test between *PFCRC*-OE-*doll1* and *doll1* mutant, $p < 0.01$. Bars = 2 mm in (B) to (G); 400 µm in (H) to (J).

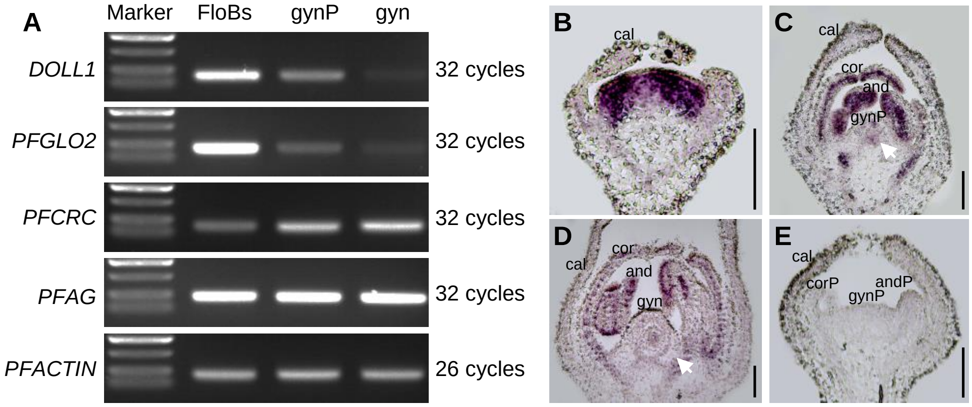

Fig. S22. Floral expression of *DOLL1*, *PFGLO2*, and *PFCRC* in *P. floridana.* (A) Semi-quantitative RT-PCR assays. Total RNAs were isolated from the indicated organs and subjected to RT-PCR assays. The amplification cycles of each gene are given. Marker, DNA ladder 2000; FloBs, floral buds of 20 days before flowering; gynP, gynoecium premordia tissues; gyn, mature gynoecium of 2 days before flowering. *PFAG* and *PFACTIN* were used as internal references. (B-E) Floral expression of *DOLL1* revealed by *in situ* hybridization. cal, calyx; cor, corolla; corP, corolla primordia; and, androecium; andP, androecium primordia; gyn, gynoecium; gynP, gynoecium primordia. Antisense nucleotide probe (B–D); sense nucleotide probe (E). Bars = 100 µm.

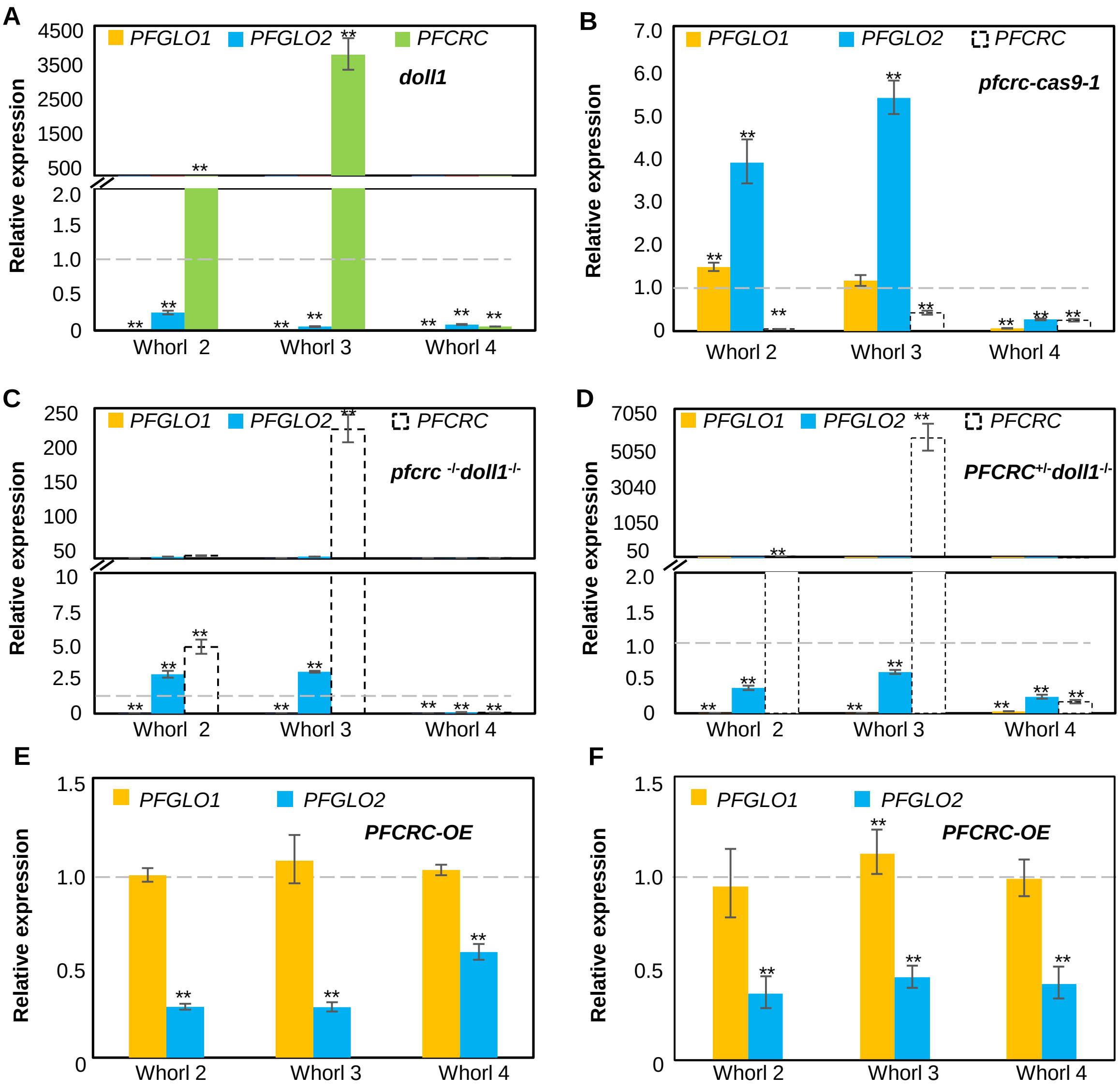

Fig. S23. Floral expression of *DOLL1 (PFGLO1)*, *PFGLO2* and *PFCRC* in different genetic backgrounds of *P. floridana*. (A) Gene expression in the *doll1* mutant. (B) Gene expression in *pfcrc-cas9-1* plants. (C) Gene expression in the $pfcrc^{-/-}doll1^{-/-}$ double mutant. (D) Gene expression in the $PFCRC^{+/-}doll1^{-/-}$ double mutant. (E) and (F) Gene expression in the *PFCRC-OE* plants. Relative expression is indicated by fold changes relative to the WT petal, stamen, and carpel organs. White dotted columns in (B-D), the edited *pfcrc* mRNA; dotted lines in (A-F), relative expression of these three genes in WT organs were set as 1.0. *PFTUBULIN* (A-E) and *PFACTIN* (F) were used as the internal reference genes. **, Student's *t*-test, $P < 0.01$.

A	***PFCRC* promoter (-2016~-1)**		***PFCRC* promoter (-4549~-2017)**	
**All CArG-boxes**	CArG1 - CArG5		CArG6 - CArG10	
**Reporter gene**	activated		activated	
**Functional CArG-box**	CArG1 and CArG4		CArG6 and CArG10	
**Consensus sequence**	CHW$_2$AAW$_2$DG (CArG1, 4, 6, 10)			CArG1：CAAAAAATTG
**Site-directed mutation**	AHW$_2$AAW$_2$DT	CHW$_2$TTW$_2$DG	CHW$_2$TAW$_2$DG	mCArG1 (CArG2)：CAAAATATTG
**Reporter gene**	no activated	no activated	no activated	no activated

B

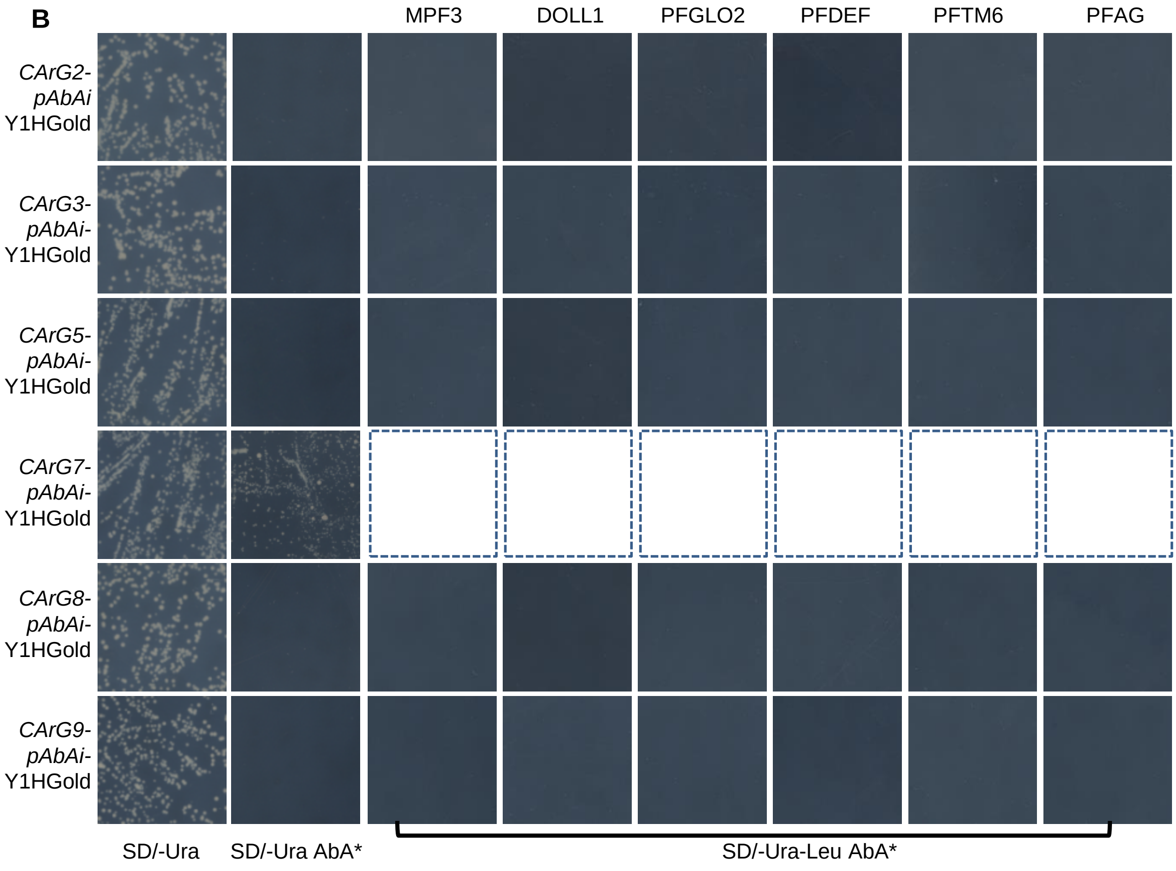

Fig. S24. Functional test of CArG-boxes in the *PFCRC* promoter by Y1H assays. (A) Binding activity of PFGLO proteins to the *PFCRC* promoter and each CArG-box. The distribution of CArG-box on each tested putative *PFCRC* promoter is given and named. The first nucleotide of ATG is defined as position 1. The red highlights the sites that have undergone site-directed mutagenesis. CArG1, 4, 6, 10 shared the consensus of CHW$_2$AAW$_2$DG type, which was bound by PFGLOs. D, G/A/T; W, A/T; H, A/T/C. However, the remaining CArG-box shared the consensus of C(A/T)$_8$G type. The sequence and distribution of the CArG-box on the *PFCRC* promoter is shown in Fig. 8 and Supplementary Table S1. (B) *P. floridana* A-, B-, and C-function MADS-domain proteins did not bind to the CArG motif of C(A/T)$_8$G type. *, AbA concentration used in Y1H of CArG-2, -3, -5, -7, -8, and -9 motifs was 700 ng/ml, 200 ng/ml, 200 ng/ml, 1000 ng/ml, 100 ng/ml, and 700 ng/ml, respectively. However, the AbA concentration was more than 1000 ng/ml in the case of CArG7, indicating a strong self-activation activity of this motif in yeast. Thus, it was not further investigated.

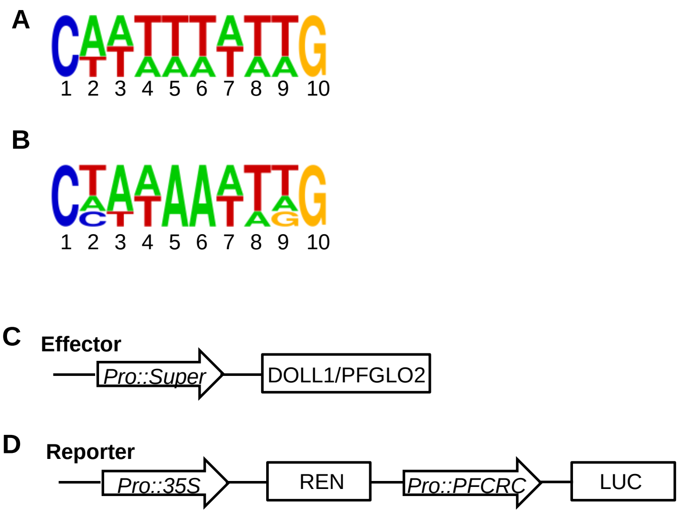

Fig. S25. The CArG-box motifs in the PFCRC promoter and constructs to detect DOLL1-*PFCRC* regulation. (A) Sequence logo of CArG2/3/5/7/8/9 motifs. (B) Sequence logo of CArG1, CArG4, CArG6 and CArG10. (C, D) Schematic diagram shows the effector and reporter constructs used in the transient transcriptional activity assays. Effector, DOLL1 or PFGLO2 driven by the *Super* promoter; reporter, firefly luciferase (LUC) driven by the defined *PFCRC* promoter (the CArG-box could be mutated); REN, the renilla luciferase gene was driven by a 35S promoter and used as an internal reference.

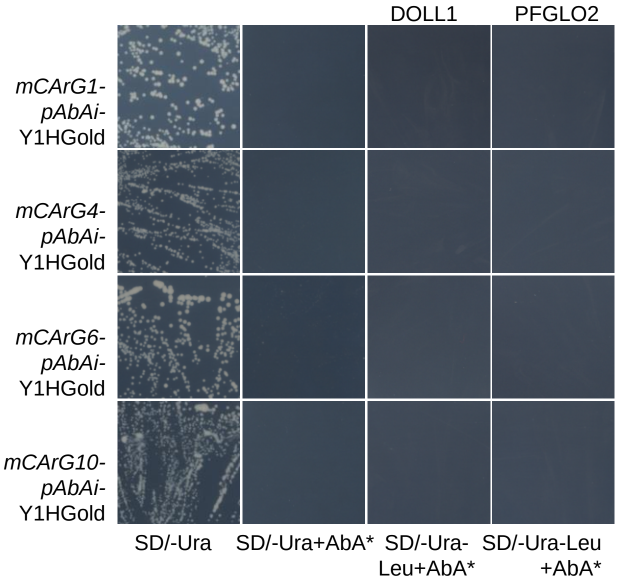

Fig. S26. Site-directed mutagenesis of the CHW_2AAW_2DG CArG-boxes in yeast. Interactions between DOLL1 (or PFGLO2) and the functional CArG-boxes were disturbed if the fifth site adenine (A) mutated to thymine (T) in Y1H analyses. *, AbA concentration used in Y1H; mCArG1, 200 ng/ml; mCArG4, 600 ng/ml; mCArG6, 900 ng/ml; mCArG10, 300 ng/ml.

A

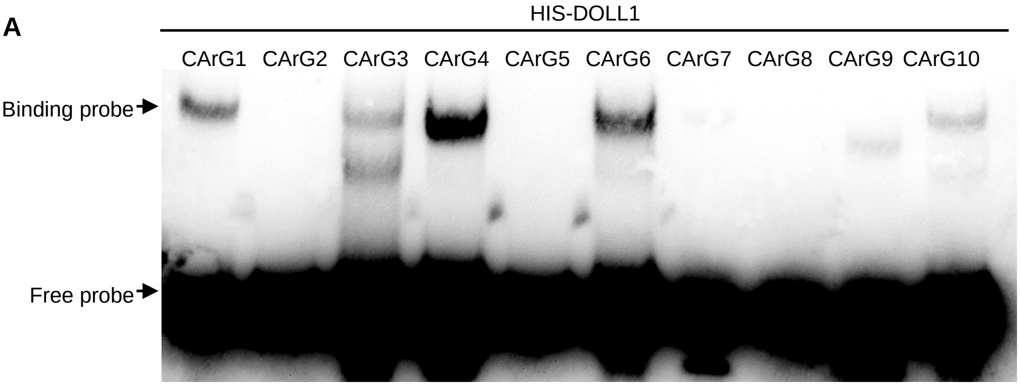

B

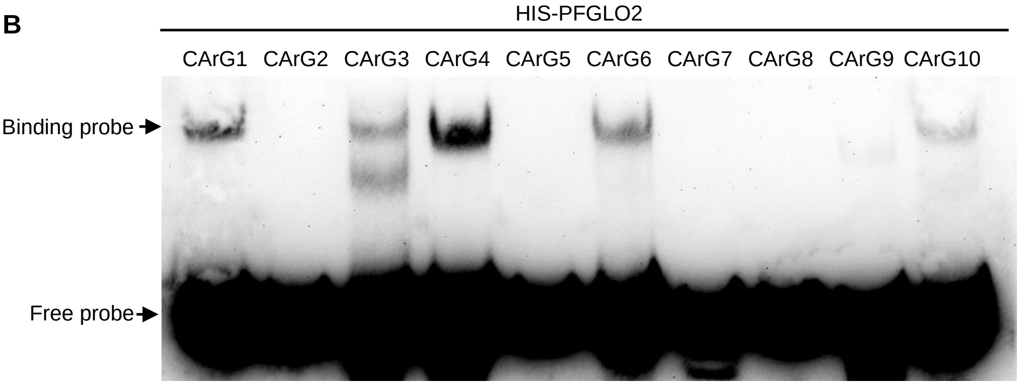

C

proPFCRC-CArG1-WT --- TGAAAAGAAT CAAAAAATTG TACTTCAAGA ---
proPFCRC-CArG1-m --- TGAAAAGAAT CAAATAATTG TACTTCAAGA ---

proPFCRC-CArG4-WT --- TGGGGGTAAT CTATAAAAAG AAATTGTAAG ---
proPFCRC-CArG4-m --- TGGGGGTAAT CTATTAAAAG AAATTGTAAG ---

proPFCRC-CArG6-WT --- TAGCTAATAC CTTTAATTTG TCTCAACTCT ---
proPFCRC-CArG6-m --- TAGCTAATAC CTTTTATTTG TCTCAACTCT ---

proPFCRC-CArG10-WT --- TCAAACTAGG CCAAAATTGG CCCGCTGAAT ---
proPFCRC-CArG10-m --- TCAAACTAGG CCAATATTGG CCCGCTGAAT ---

Fig. S27. Binding detection of HIS-DOLL1 and HIS-PFGLO2 fusion proteins to all CArG-box motifs of the *PFCRC* promoter in EMSA assays. (A) HIS-DOLL1 fusion protein binds to CArG-box motifs in EMSA assay. (B) HIS-PFGLO2 fusion protein binds to CArG-box motifs in EMSA assay. (C) The design of the wild type (WT) probe containing the CArG-box (CArG1-, CArG4-, CArG6-, and CArG10-WT) and the mutated probe (CArG1-, CArG4-, CArG6-, and CArG10-m) in the *PFCRC* promoter. Mutated bases are highlighted in red.

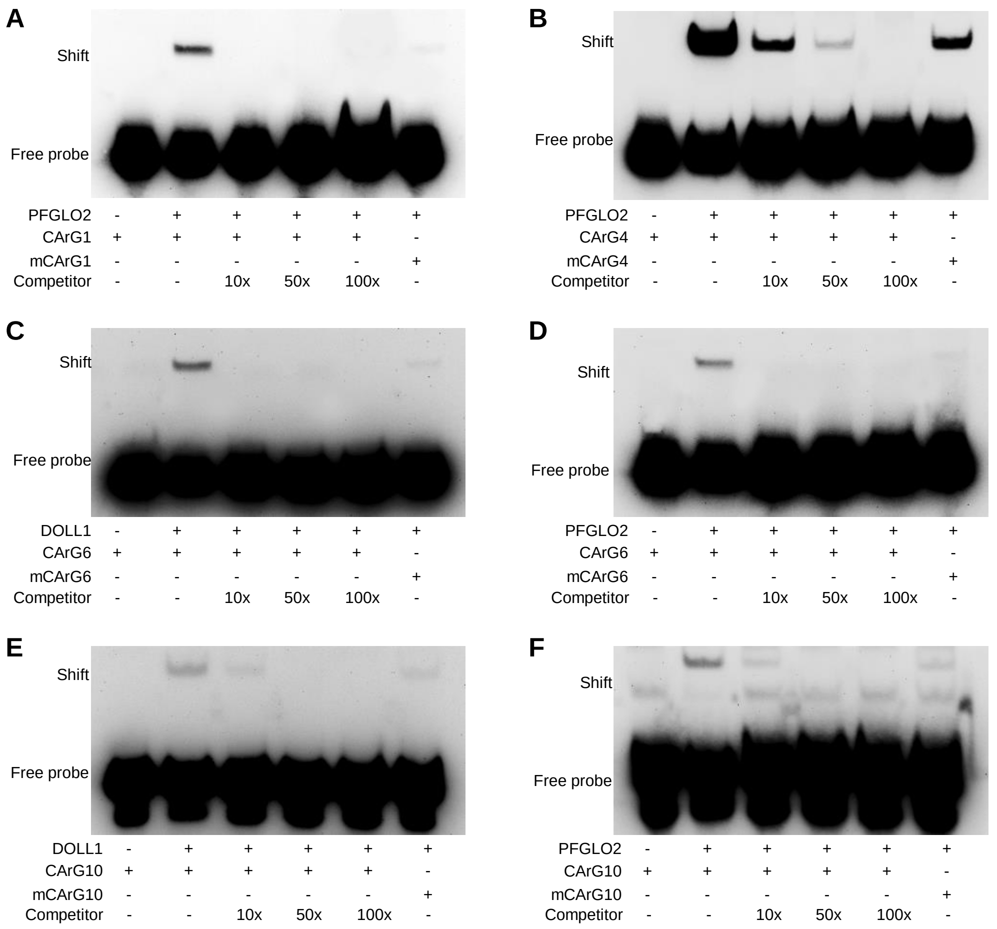

Fig. S28. Competition and site-directed mutagenesis of HIS-DOLL1 and HIS-PFGLO2 fusion proteins binding to the functional CArG-box motifs in the *PFCRC* promoter. (A, B) HIS-PFGLO2 fusion proteins bind to CArG1 and CArG4 motif by the EMSA assay. (C) HIS-DOLL1 fusion proteins bind to CArG6 motif by the EMSA assay. (D) HIS-PFGLO2 fusion proteins bind to CArG6 motif by the EMSA assay. (E) HIS-DOLL1 fusion proteins bind to CArG10 motif by the EMSA assay. (F) HIS-PFGLO2 fusion proteins bind to CArG10 motif by the EMSA assay. +, indicates the presence; –, the absence of corresponding components as indicated; mCArG1, mCArG4, mCArG6, and mCArG10, mutated probes; competitor, unlabeled of WT CArG-box motif probes.

A

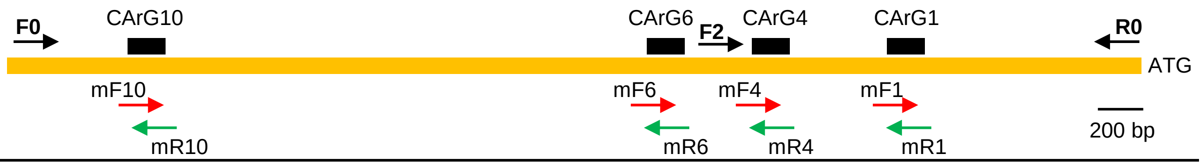

B

**Designed *PFCRC* promoter**	**1st round PCR**	**2st round PCR**
CArG4-CArG1	F2+R0	-
CArG4-mCArG1	F2+mR1; mF1+R0	F2+R0
mCArG4-CArG1	F2+mR4; mF4+R0	
mCArG10-mCArG6-mCArG4-mCArG1	F0+mR10; mF10+mR6; mF6+mR4; mF4+mR1; mF1+R0	F0+R0
mCArG10-mCArG6-mCArG4-CArG1	F0+mR10; mF10+mR6; mF6+mR4; mF4+R0	
mCArG10-mCArG6-CArG4-mCArG1	F0+mR10; mF10+mR6; mF6+mR1; mF1+R0	
mCArG10-CArG6-mCArG4-mCArG1	F0+mR10; mF10+mR4; mF4+mR1; mF1+R0	
CArG10-mCArG6-mCArG4-mCArG1	F0+mR6; mF6+mR4; mF4+mR1; mF1+R0	

C

**Primer name**	**primer sequence 5' - 3'**
F0	CGGTATCGATAAGCTTTATAAGGACAGTCAAACTAGGCC (*Hind* III)
F2	CGGTATCGATAAGCTTGTGATTCAGTTCGACATATATTAG (*Hind* III)
R0	TAGAACTAGTGGATCCGGAGTTCTTTTCAGCTGAAGGAG (*BamH* I)
mF1	CTATTGAAAAGAATCAAATAATTGTACTTC
mR1	ATTTGATTCTTTTCAATAGATCTCCTCG
mF4	TTGGGGGTAATCTATTAAAAGAAATTG
mR4	AATAGATTACCCCCAATTGATATTAA
mF6	ATAGCTAATACCTTTTATTTGTCTCA
mR6	AAAAGGTATTAGCTATATAGGGTTAATG
mF10	GTCAAACTAGGCCAATATTGGCCCCGC
mR10	ATTGGCCTAGTTTGACTGTCCTTATA

Fig. S29. Schematic diagram of *PFCRC* promoter pGreenII 0800-LUC related plasmid construction. (A) Location of CArG-box and the designed primers on the *PFCRC* promoter. (B) Primer combinations for the designated mutational fragments for the constructs. (C) Primer sequence information in this assay. The restriction enzyme sites and the mutated sites are highlighted in red.

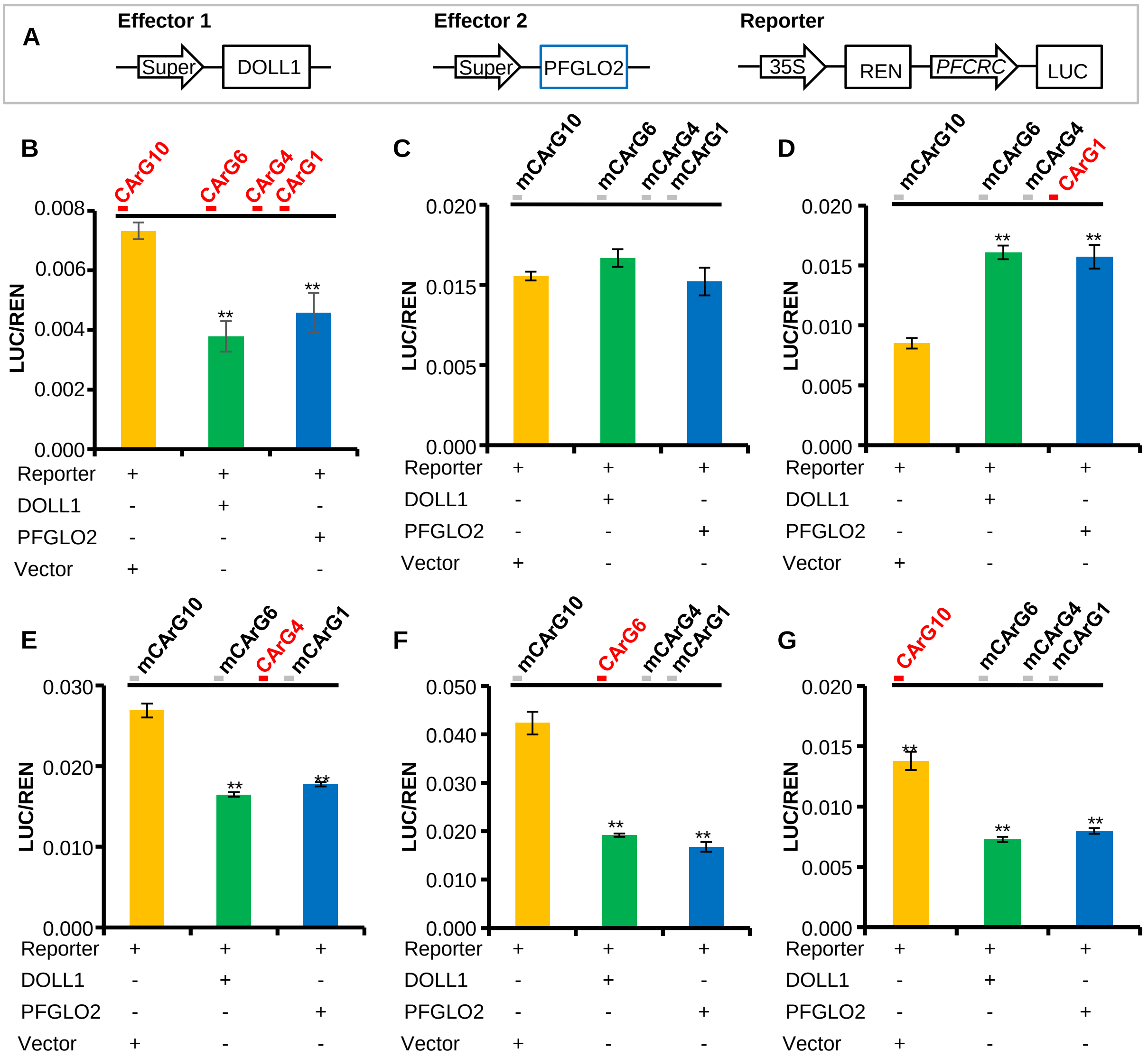

Fig. S30. PFGLOs regulate *PFCRC* expression in a dual-luciferase assay. (A) Schematic diagram shows the constructs used in the transient transcriptional activity assays. Effector, DOLL1 or PFGLO2 driven by the *Super* promoter; Reporter, firefly luciferase (LUC) driven by the *PFCRC* promoter region containing the four functional CArG-box motifs, and its mutated versions. REN, the renilla luciferase gene was driven by a 35S promoter and used as an internal reference. (B) DOLL1 or PFGLO2 protein had a repressing role on the *PFCRC*–LUC expression. (C) DOLL1 or PFGLO2 protein did not have a regulatory role on the *PFCRC*–LUC expression when all four CArG-box motifs were mutated. (D) DOLL1 or PFGLO2 proteins activate the *PFCRC*-LUC expression via binding to the CArG1-box motif. (E) DOLL1 or PFGLO2 proteins repress the PFCRC–LUC expression via binding to the CArG4-box motif. (F) DOLL1 or PFGLO2 proteins repress the *PFCRC*-LUC expression via binding to the CArG6-box motif. (G) DOLL1 or PFGLO2 proteins repress the *PFCRC*-LUC expression via binding to the CArG10-box motif. In (B-F), values on the Y-axis are means of LUC/REN $\pm$ SD (n = 3); the vector is the empty *Superp1300-GFP* plasmid used as a control; +, indicates the presence; –, the absence of corresponding components as indicated; black lines represent the 4.5 kb putative *PFCRC* promoter sequence upstream of the start codon; double stars ** represent significant differences ($P < 0.05$) by Student's t-test.

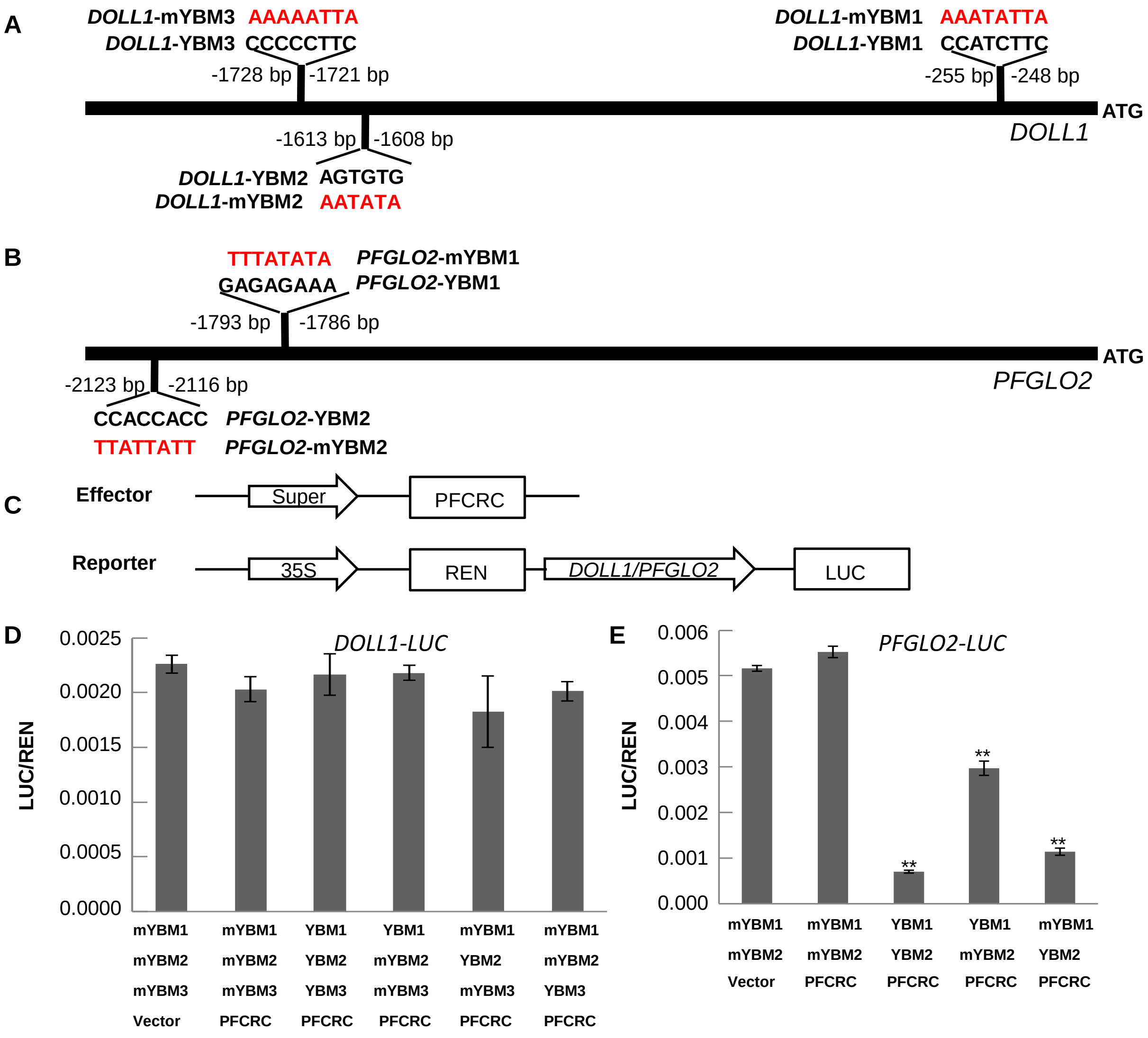

Fig. S31. PFCRC represses *PFGLO2* expression. (A) Schematic diagram shows three putative YABBY binding motifs (*DOLL1*-YBM1, -YBM2 and -YBM3) in an 1838 bp *DOLL1* promoter sequence upstream from the start codon (ATG). Numbers represent the position of each YBM motif located. m, each mutated YMB sequence; mutated bases are highlighted in red. (B) Schematic diagram shows three putative YABBY binding motifs (*PFGLO2*-YBM1, -YBM2 and -YBM3) in a 2194 bp *PFGLO2* promoter sequence upstream from the start codon (ATG). Numbers represent the position of each YBM motif located. m, each mutated YMB sequence; mutated bases are highlighted in red. (C) Schematic diagram shows the constructs used in the transient transcriptional activity assays. Effector, PFCRC driven by the *Super* promoter, Reporter, firefly luciferase (LUC) driven by the *DOLL1* or *PFGLO2* promoters containing the putative YBMs and their variants; REN, the renilla luciferase gene was driven by a 35S promoter and used as an internal reference. (D) Altering the putative YMBs in the *DOLL1* promoter did not alter the DOLL1-LUC expression, suggesting that PFCRC did not regulate the *DOLL1* expression. (E) Altering the putative YMBs in the *PFGLO2* promoter did alter the PFGLO2-LUC expression, suggesting that PFCRC repressed *PFGLO2* via binding to these YABBY-motifs. The vector is the empty *Superp1300-GFP* plasmid used as a control. Values on the Y-axis are means of LUC/REN ± SD (n = 3). ** represents significant differences $P < 0.05$ by Student's t-test.

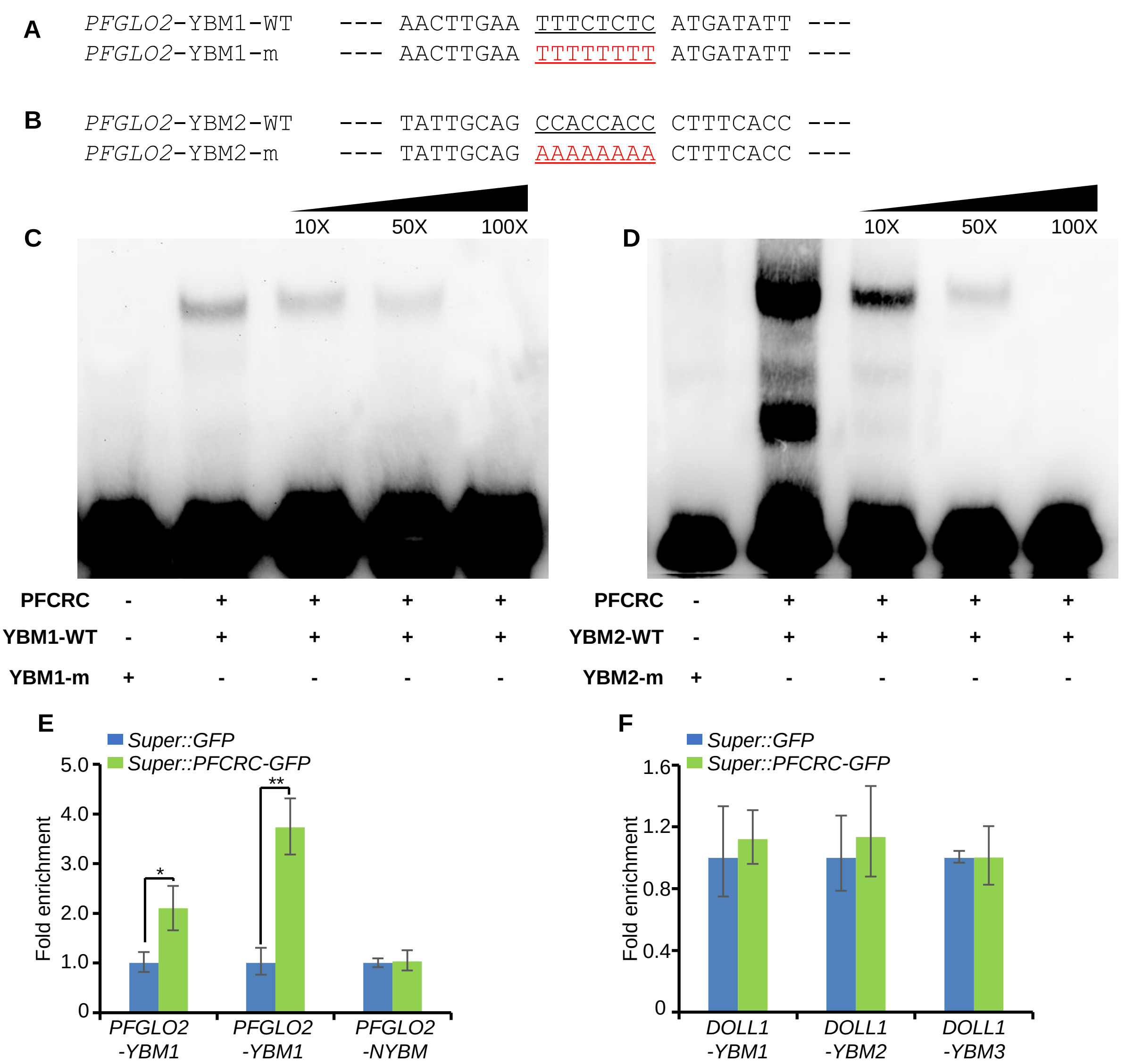

Fig. S32. Binding ability of PFCRC to the YBM motifs in the *PFGLO2* promoter. (A-D) Binding ability of HIS-PFCRC fusion protein to the YBM motifs in the *PFGLO2* promoter by the EMSA assay. (A) The design of the wild type (WT) probe containing the YBM motif (*PFGLO2*-YBM1-WT) and the mutated probe (*PFGLO2*-YBM1-m) in the *PFGLO2* promoter. (B) The design of the WT probe containing the YBM motif (*PFGLO2*-YBM2-WT) and the mutated probe (*PFGLO2*-YBM2-m) in the *PFGLO2* promoter. Mutated bases are highlighted in red. (C) HIS-PFCRC fusion protein binds the YBM1 motif of PFGLO2 by the EMSA assay. (D) HIS-PFCRC fusion protein binds the YBM2 motif of PFGLO2 by the EMSA assay. +, indicates the presence; –, the absence of the corresponding components as indicated. (E, F) Chromatin immunoprecipitation (ChIP) analysis of PFCRC associated with the YBMs of *PFGLO2* and *DOLL1* promoters. (E) CHIP analysis of PFCRC with putative YBMs of the *PFGLO2* promoter. NYBM, random fragments harboring non-YBM motif in the promoter, used as a negative control. (**F**) CHIP analysis of PFCRC with putative YBMs of the *DOLL1* promoter. Bars. SD from the three biological replicates. *, *Student's t*-test, $p < 0.05$, **, $p < 0.01$.

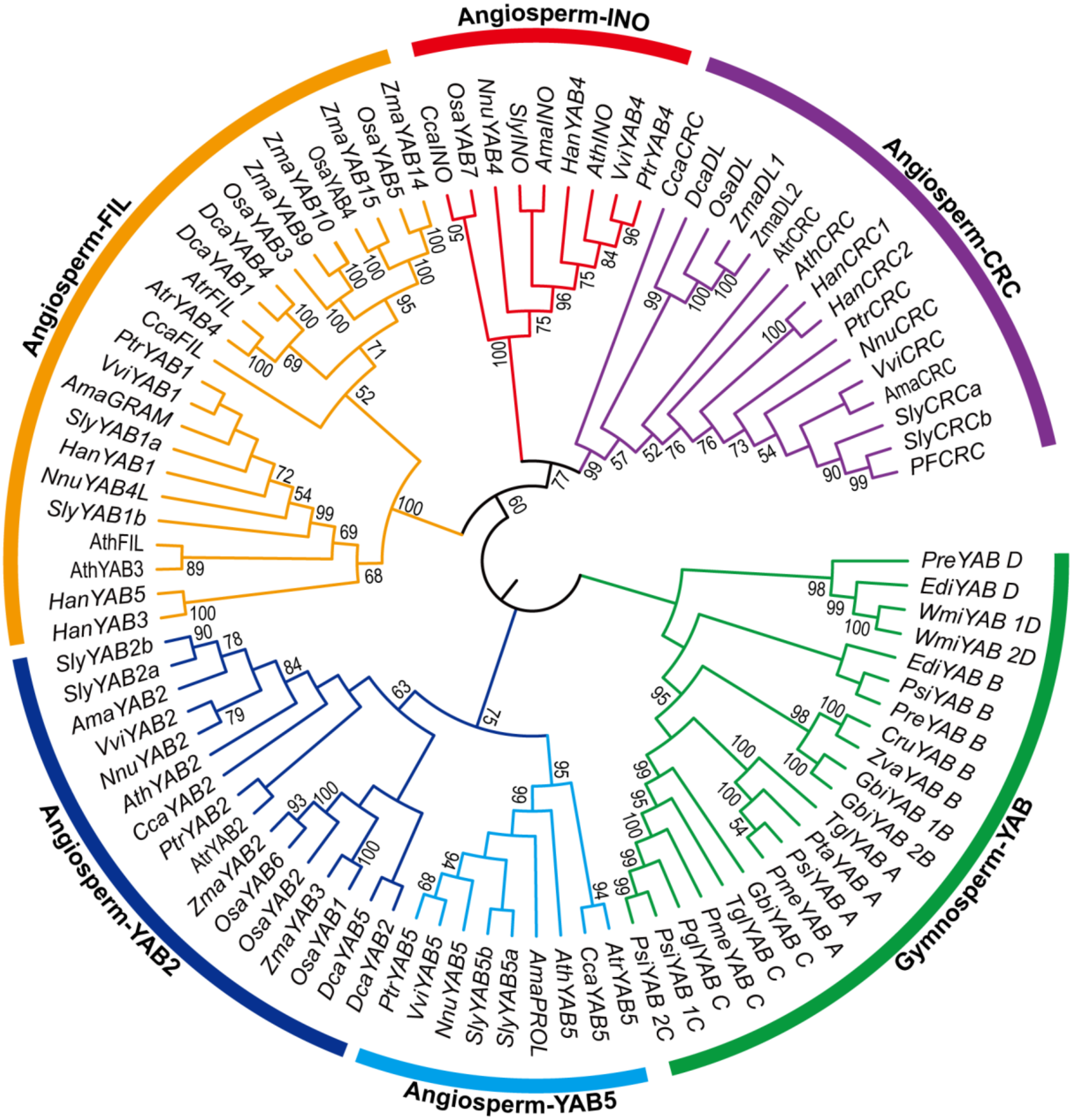

Fig. S33. Phylogeny of *YABBY* genes in seed plants. The ML phylogenetic tree was constructed using MEGA. Gymnosperm (green arc) and angiosperm *YABBY* genes form a monophyletic clade, suggesting a single *YABBY* gene in the ancestor of extant seed plants. *YABBY* genes in angiosperms were clustered into five groups. The outer arcs are marked in light blue, blue, orange, red, and purple, which respectively represent the *YABBY5*-, *YABBY2*-, *FIL*-, *INO*-, and *CRC*-like groups. Species abbreviations, gene accession numbers and other information are listed in Supplementary Table S6. Bootstrapping was used to test the tree, and only values > 50 are displayed near the nodes.

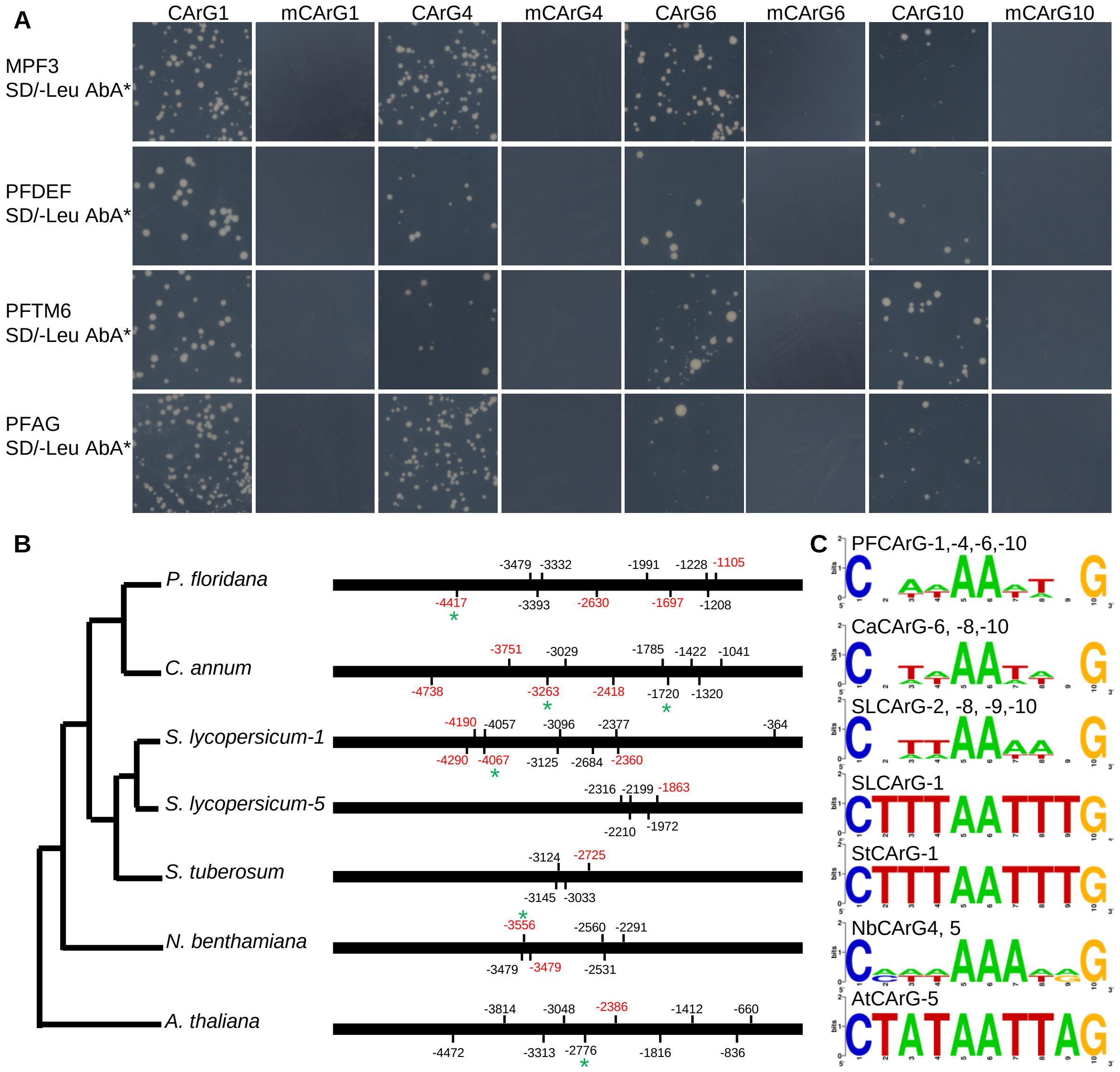

Fig. S34. Conservation of CArG-box motifs in Solanaceous species. (A) Floral MADS-domain proteins did bind to the functional CArG-box motifs in the *PFCRC* promoter. (B) CArG-box motifs were distributed along the promoters of *CRC* orthologs. The putative functional CArG-boxes are highlighted in red, and they were predicted according to the knowledge obtained from *Physalis floridana* in this work. (C) The sequences of the putative functional CArG-boxes in the indicated species.
